# *Clostridium difficile* Biofilm: Remodeling Metabolism and Cell Surface to Build a Sparse and Heterogeneously Aggregated Architecture

**DOI:** 10.3389/fmicb.2018.02084

**Published:** 2018-09-12

**Authors:** Isabelle Poquet, Laure Saujet, Alexis Canette, Marc Monot, Jovanna Mihajlovic, Jean-Marc Ghigo, Olga Soutourina, Romain Briandet, Isabelle Martin-Verstraete, Bruno Dupuy

**Affiliations:** ^1^Micalis Institute, Institut National de la Recherche Agronomique, AgroParisTech, Université Paris-Saclay, Jouy-en-Josas, France; ^2^Laboratoire Pathogenèse des Bacteries Anaerobies, Institut Pasteur, Paris, France; ^3^Sorbonne Paris Cité, Université Paris Diderot, Paris, France; ^4^Unité de Génétique des Biofilms, Institut Pasteur, Paris, France

**Keywords:** *Clostridium difficile*, biofilm formation, gene expression profiling, biofilm architecture, aggregates

## Abstract

*Clostridium difficile* is an opportunistic entero-pathogen causing post-antibiotic and nosocomial diarrhea upon microbiota dysbiosis. Although biofilms could contribute to colonization, little is known about their development and physiology. Strain 630Δ*erm* is able to form, in continuous-flow micro-fermentors, macro-colonies and submersed biofilms loosely adhesive to glass. According to gene expression data, in biofilm/planktonic cells, central metabolism is active and fuels fatty acid biosynthesis rather than fermentations. Consistently, succinate is consumed and butyrate production is reduced. Toxin A expression, which is coordinated to metabolism, is down-regulated, while surface proteins, like adhesins and the primary Type IV pili subunits, are over-expressed. C-di-GMP level is probably tightly controlled through the expression of both diguanylate cyclase-encoding genes, like *dccA*, and phosphodiesterase-encoding genes. The coordinated expression of genes controlled by c-di-GMP and encoding the putative surface adhesin CD2831 and the major Type IV pilin PilA_1_, suggests that c-di-GMP could be high in biofilm cells. A *Bacillus subtilis* SinR-like regulator, CD2214, and/or CD2215, another regulator co-encoded in the same operon as CD2214, control many genes differentially expressed in biofilm, and in particular *dccA*, *CD2831* and *pilA_1_* in a positive way. After growth in micro-titer plates and disruption, the biofilm is composed of robust aggregated structures where cells are embedded into a polymorphic material. The intact biofilm observed *in situ* displays a sparse, heterogeneous and high 3D architecture made of rods and micro-aggregates. The biofilm is denser in a mutant of both *CD2214* and *CD2215* genes, but it is not affected by the inactivation of neither *CD2831* nor *pilA_1_*. *dccA*, when over-expressed, not only increases the biofilm but also triggers its architecture to become homogeneous and highly aggregated, in a way independent of *CD2831* and barely dependent of *pilA_1_*. Cell micro-aggregation is shown to play a major role in biofilm formation and architecture. This thorough analysis of gene expression reprogramming and architecture remodeling in biofilm lays the foundation for a deeper understanding of this lifestyle and could lead to novel strategies to limit *C. difficile* spread.

## Introduction

*Clostridium difficile* is a Gram-positive, spore-forming obligate anaerobe and an opportunistic entero-pathogen (Leffler and Lamont, 2015; Abt et al., 2016; Smits et al., 2016). A healthy gut microbiota plays a major role in resistance to colonization and control of *C. difficile* infections (Theriot and Young, 2015; Abt et al., 2016). After an antibiotic treatment leading to a dysbiotic microbiota, *C. difficile* expansion is favored and can lead to severe and potentially fatal colitis and diarrhea including nosocomial diarrhea. Infection recurrence is an alarming problem because relapses are difficult to treat. *C. difficile* represents both a public health problem and an economic burden in industrialized countries (Leffler and Lamont, 2015; Abt et al., 2016; Smits et al., 2016).

*C. difficile* life cycle is complex (Smits et al., 2016) and begins when a host is orally contaminated by environmental or hospital spores. In a dysbiotic microbiota, metabolism is modified and favors spore germination and vegetative cell expansion (Theriot and Young, 2015; Abt et al., 2016). Adhesion and stress-adaptation factors contribute to colonization efficiency (Abt et al., 2016; Janoir, 2016). Infection culminates with the tightly controlled production of Tcd toxins (Martin-Verstraete et al., 2016). These virulence factors are glycosyl-transferases inactivating Rho/Ras/Rac GTP-binding proteins, thus ultimately leading to colonic epithelium damage and pain (Abt et al., 2016). Cell sporulation concludes the cycle, allowing dissemination and spread of highly resistant infectious forms (Smits et al., 2016).

*C. difficile* relapsing infections ressemble chronic infections, which are often associated to pathogen biofilms (Hall-Stoodley and Stoodley, 2009). In these communities embedded into a matrix (Hobley et al., 2015), cells are protected against environmental fluctuations and stresses, including antibiotics and host defenses (Hall-Stoodley and Stoodley, 2009). *C. difficile* has first been shown to form biofilms *in vitro*, on different abiotic surfaces and in different growth systems (Dapa and Unnikrishnan, 2013; Pantaleon et al., 2014; Janoir, 2016). These biofilms contribute to *C. difficile* resistance to oxygen and to its tolerance to antibiotics, including metronidazole and vancomycin used to treat infected patients (Dawson et al., 2012; Dapa et al., 2013; Semenyuk et al., 2014; Mathur et al., 2016; James et al., 2017). *C. difficile* can form part of a multi-species biofilm in a chemostat mimicking the human gut (Crowther et al., 2014) and to multi-species gut communities *in vivo* (Semenyuk et al., 2015). Importantly, mono-species biofilm-like structures have recently been identified *in vivo*, in gnotobiotic mice (Soavelomandroso et al., 2017).

Most matrix components and factors required for biofilm formation in other species are not conserved in *C. difficile*. Biofilm development involves Type IV pili in several species (Melville and Craig, 2013), and in one *C. difficile* strain, R20291, the major Type IV pilin PilA_1_ slightly increases early biofilm formation by contributing to cell clumping (Purcell et al., 2015; Maldarelli et al., 2016). PSII, an atypical teichoic acid (Ganeshapillai et al., 2008; Reid et al., 2012) anchored at the cell surface by the ligase LcpB, might promote biofilm formation when shedded into the medium, as biofilm biomass increases in a *lcpB* mutant (Chu et al., 2016). Finally, the glycosylation of flagellin, which is required for motility, impairs auto-aggregation and biofilm formation (Valiente et al., 2016), suggesting that biofilm cells are mainly sessile or at least impaired in flagella-driven motility.

Biofilm formation in *C. difficile* is regulated by Spo0A, the master regulator of sporulation initiation (Dawson et al., 2012; Dapa et al., 2013). A positive regulation by a quorum sensing mechanism has also been proposed (Dapa et al., 2013). The secondary messenger c-di-GMP, whose level can be increased by over-producing the c-di-GMP synthase DccA, promotes biofilm formation and culture aggregation, while inhibiting motility (Purcell et al., 2012, 2015; Soutourina et al., 2013; Bordeleau et al., 2015). The inactivation of the phospho-di-esterase PdcA, which degrades c-di-GMP, also increases biofilm formation (Purcell et al., 2017). C-di-GMP modulates gene expression through riboswitches: it represses the flagellar operon and induces a few genes encoding surface proteins, like PilA_1_ (Purcell et al., 2012; Soutourina et al., 2013; Peltier et al., 2015). In a *dccA* over-expression context, PilA_1_ slightly contributes to biofilm formation and culture aggregation (Bordeleau et al., 2015; Purcell et al., 2015).

Here, continuous-flow micro-fermentors were set up under anaerobiosis to grow a model toxinogenic strain as a pure biofilm in the absence of planktonic cells. Global gene expression was compared between biofilm and planktonic bacteria and the role of CD2214, a SinR-like regulator (Saujet et al., 2011), and CD2215, a regulator co-encoded in the same operon as CD2214, was determined. CD2214–CD2215 regulators, and two surface proteins whose genes are up-regulated in biofilm were studied for their role in biofilm formation in micro-fermentors and in microtiter plates. Biofilm architecture was observed by confocal laser scanning microscopy (CLSM). Finally, *C. difficile* was found to form, at both the macroscopic and microscopic levels, a biofilm of original properties and architecture.

## Materials and Methods

### Strains, Growth Conditions and Plasmids

*Escherichia coli* strains (**Supplementary Table S1A**) were grown in Luria-Bertani (LB) medium and ampicillin (100 μg/ml) or chloramphenicol (15 μg/ml) were used for plasmid selection. *C. difficile* model toxinogenic strain 630Δ*erm* and its mutants (**Supplementary Table S1A**) were grown anaerobically (90% N_2_, 5% CO_2_ and 5% H_2_) at 37°C in a Freter cabinet. Routine growth was in BHI medium. Thiamphenicol (15 μg/mL) together with Cefoxitin (25 μg/ml) or BCC (Oxoid) were used to select for transconjugants and erythromycin (2.5 μg/mL) to select for mutants. For routine growth of strains bearing plasmids, thiamphenicol (15 μg/mL) was always added to maintain plasmids whereas for Clostron mutants, erythromycin (2.5 μg/mL) was added on plates and omitted in liquid cultures. Biofilms (see below) and planktonic cultures (‘batch’ cultures in Falcon tubes) were grown in Tryptone Yeast Extract (TY) supplemented or not by 0.1% sodium thioglycolate (TYt). Finally, to induce the *P_tet_* promoter from pDIA6103 (p) and from pDIA5987 (p*dccA*), where *P_tet_* controls a cloned ectopic *dccA* copy (**Supplementary Table S1B**), anhydro-tetracycline was added at 100 ng/mL during ON pre-cultures used for biofilm growth (Soutourina et al., 2013). Plasmids (**Supplementary Table S1B**) were constructed using standard procedures.

### Biofilm Growth

Biofilms were grown at 37°C in continuous-flow glass micro-fermentors as described^1^ (Ghigo, 2001) except under anaerobiosis. 1 mL of an ON culture grown in TYt medium and diluted to an OD_600_ of 1 was inoculated into TYt medium (60 mL). Medium and anaerobic gas flows were immediately applied without bubbling. The medium speed was first set to 2 for 3–4 h and then increased to 6. After 72 h, most medium (∼45 mL) was gently removed and all biomass was recovered after vortexing in the remaining medium.

Biofilms were also grown in polystyrene micro-titer plates (Greiner BioOne). Biofilms were grown in 24-well micro-titer plates, either from initially adhesive cells or from cells of an ON pre-culture diluted at 1/100, and in 96-well micro-titer plates exclusively from initially adhesive cells. For this initial adhesion step in either 24- or 96-well micro-titer plates, an aliquote (500 and 125 μL, respectively) of an ON pre-culture grown in TYt medium was diluted twice in the same medium and incubated for 2 h 30. After carefully removing all the liquid, fresh TYt medium (1 mL or 250 μL, respectively) was added onto adhesive cells. Plates carefully placed into anaerobic bags (BD Difco^TM^ GasPack EZ Gas Generating Systems) were incubated at 37°C for 1 to 2 day(s). Biofilms grown in 24-well micro-titer plates were recovered by pipetting, fixed in the presence of paraformaldehyde at 4%, washed and resuspended in PBS 1X before being observed by Transmitted Light Microscopy using a Axio Observer Z1 Zeiss microscope. Intact biofilms were grown in 96-well micro-titer plates for either 24 h or 48 h, and in the case of strains over-expressing *dccA*, in the presence of anhydro-tetracycline, at 300 ng/mL during adhesion, and at 500 ng/mL during biofilm growth as previously described (Soutourina et al., 2013). Intact biofilms were finally observed *in situ*, without any pre-treatment, by a non-invasive method, Confocal Laser Scanning Microscopy.

### RNA Extraction and RT-qPCR Analysis

Cells washed in PBS buffer at 4°C were treated by the Fast RNA Pro Blue kit in a FastPrep apparatus (MP Biomedicals) as recommended. RNAs could efficiently be extracted from the important recovered biofilm biomass. After treatment by TURBO DNase (Ambion), total RNAs (1 μg) were mixed to pdN6 (1 μg; Roche), heated for 10 min at 70°C and cooled on ice. Reverse transcription using Avian Myeloblastosis Virus Reverse Transcriptase (Promega), dNTP (2 mM final) and RNasin^®^ (40 units) was performed at 37°C for 2 h and stopped at 85°C for 5 min. RT-qPCR was performed using cDNAs (20 ng), specific primers (200 nM, **Supplementary Table S1C**) and the FastStart Universal SYBR Green Master Mix (ROX, Roche). Amplification and detection were as previously described (Saujet et al., 2011). cDNA quantities were normalized by comparison to *dnaF-CD1305* gene as a reference (Saujet et al., 2013). The relative change in gene expression was the ratio of normalized target concentrations (threshold cycle [ΔΔ*C_T_*] method) (Livak and Schmittgen, 2001).

### Microarrays

The microarray of strain 630 genome (GEO database accession number GPL10556) was used as previously described (Saujet et al., 2011). RNA quality was checked using Agilent RNA 6000 Nano kit and 2100 Bioanalyser apparatus as recommended. For each condition, four independent RNA samples (10 μg) were used. First-strand cDNA synthesis and labeling were performed using a SuperScript Indirect cDNA labeling kit (Invitrogen) and Cy3 and Cy5 fluorescent dyes (GE Healthcare). Cy3- and Cy5-labeled cDNAs (200 pmol each) were mixed, and hybridized to microarrays for 17 h at 65°C. After array scanning, the data were analyzed using R and limma software (Linear Model for Microarray Data) from the Bioconductor project^2^. For each slide, background correction was performed using the normexp method, leading to strictly positive values and to reduced variability in the log ratios for genes with low levels of hybridization signal. Each slide was then normalized by the loess method. The significance of a variation in gene expression was evaluated using the Bayesian adjusted *t* statistics and a Benjamini–Hochberg multiple testing correction based on the false discovery rate. A gene was considered to be differentially expressed when the *p*-value was <0.05. The complete data sets are available from the GEO database with the following accession numbers: (i) GSE85980^3^ and (ii) GSE100946 (Token: wxitckyehfgnjyz). For result analysis, gene organization as operons was inferred from genome-wide mapping of Transcriptional Start Sites in strain 630Δ*erm* (Soutourina et al., 2013).

### Gas Phase Chromatography (GPC)

Biofilms and planktonic cultures were grown in parallel in TYt medium for 72 h, in microfermentors (60 mL) and Falcon tubes (15 mL), respectively. Most of the medium (∼45 mL for biofilms, ∼10 mL for planktonic cultures) was recovered 1 h 30 after stopping the medium flow in micro-fermentors. After centrifugation at low speed at 4°C, supernatants were sterilized on 0.22 μm filters and kept at -80°C before use. Volatile and non-volatile fatty acids were identified and quantified using a Gas Chromatograph (Model CP3380, Varian Inc., United States) as previously described (Carlier and Sellier, 1989). For each fatty acid, the concentration initially present in TYt medium was substracted from the concentration in the medium after growth. The resulting concentration was standardized by comparison to the total (all fatty acids). The mean of four independent samples is shown.

### ClosTron Mutants

ClosTron mutagenesis was performed as previously described (Saujet et al., 2011). Briefly, to retarget the group II intron for specific insertion into a gene of interest, primers (**Supplementary Table S1C**) were designed with Targetron design software (Sigma-Aldrich). Each specific overlap PCR product was sequenced and cloned into either pMTL007 or pMTL007C-E5 (**Supplementary Table S1B**). After intron sequencing, each resulting plasmid was transformed into *E. coli* HB101(RP4). The resulting donor strain and strain 630Δ*erm* were mated, and transconjugants were submitted to erythromycin selection to obtain mutant candidates. After chromosomal DNA extraction, the correct insertion of the retargeted group II intron into the gene of interest leading to its inactivation was verified by PCR as soon as candidate mutants were obtained. This experiment was subsequently repeated for all mutants together after growth in micro-fermentors and confirmed that even in the absence of erythromycin selection for 72 h, the mutation (Intron insertion) remained stable without any detectable reversion to a wild-type gene (**Supplementary Figure S1**).

### Confocal Laser Scanning Microscopy (CLSM)

After growth in micro-titer plates, biofilms were anaerobically stained for 2 h by the Filmtracer^TM^ LIVE/DEAD^®^ Biofilm Viability Kit (Thermo Fisher Scientific) at a 1/350 dilution (DNA-dyes SYTO 9 at 9.5 μM and propidium iodide at 57.1 μM, both labeling DNA, but only in damaged-membrane cells for the latter). The plate was then placed on the motorized stage of an inverted confocal microscope (TCS SP8 AOBS, Leica Microsystems) at INRA-MIMA2 platform^4^. Observations were performed using a 63×/1.2 N.A. water immersion objective lens (300 μm working distance). Fluorescent excitation was by a 488 nm argon laser line set at 30% intensity with argon potentiometer and 10% with corresponding AOTF. Emitted fluorescence was recorded with AOBS system by 2 simultaneous PMT, within a range of 497–570 nm to visualize green fluorescence corresponding to Syto 9, and between 637 and 715 nm to record red fluorescence emitted by propidium iodide. Acquisitions were performed with the LAS X High Content Screening A Matrix Screener module. Single 2D xy sections were acquired at a scan speed of 600 Hz in bidirectional mode, with a definition of 512 pixels × 512 pixels (corresponding to a field size of 184.52 μm × 184.52 μm) and a z-step of 1 μm between xy sections leading to a z-stack. Images were obtained using IMARIS 7.7.2 software (Bitplane, Switzerland): three-dimensional projections were reconstructed and orthogonal views were extracted using, respectively, the blend mode of Easy 3D function and the section mode. Several parameters describing the architecture of each biofilm: biovolume (× 1000 μm^3^), mean thickness (μm), maximum coverage (%) and biovolume/surface ratio (μm) were extracted using a home-made ICY routine software (Bridier et al., 2010). After quantification of these parameters for eight to eleven independent biofilms *per* strain (all grown from independent clones in two to three different experiments, and observed in duplicates), the results were statistically analyzed.

### Statistics

The significance of GPC and CLSM data (for microarray data, see above) was evaluated by ANOVA variance analyses using Statgraphics software (Manugistic^TM^, Rockville, MD, United States). Differences were considered as statistically significant when *p*-values associated with the Fischer test were <0.05.

## Results and Discussion

### Biofilm of Strain 630Δ*erm* Grown in Continuous-Flow Micro-Fermentors

**FIGURE 1:**
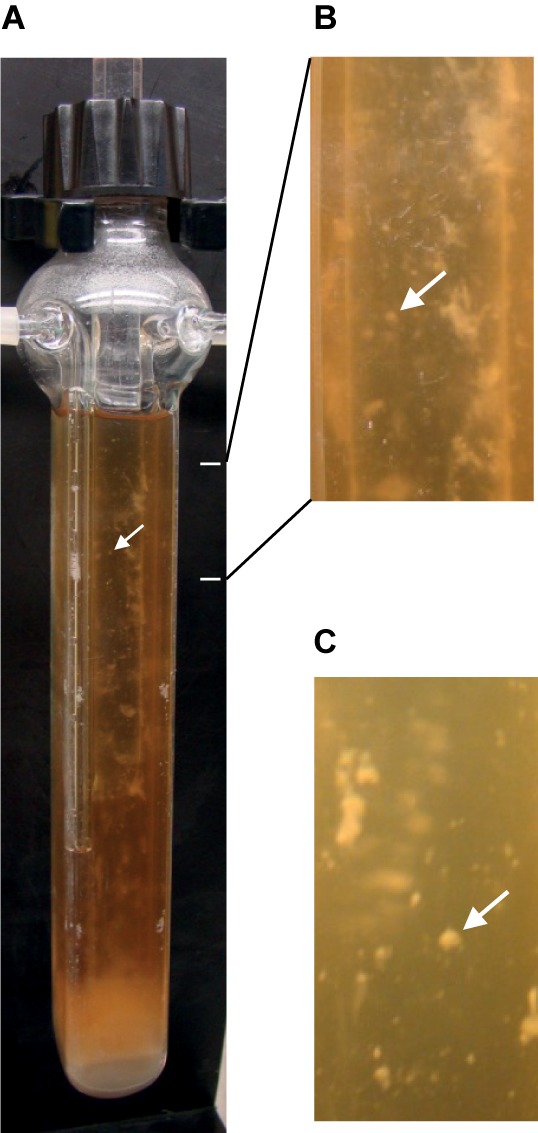
Biofilm of strain 630Δ*erm* after growth in a continuous-flow micro-fermentor. After anaerobic growth for 72 h in TYt medium, macro-colonies and biofilms can be observed on micro-fermentor walls. The medium is clear, as expected in the absence of planktonic growth. A representative picture of independent experiments is shown **(A)**. Magnifications **(B,C)** allow observing macro-colonies (arrows).

An efficient system for biofilm development was adapted here to anaerobiosis for *C. difficile*. Continuous-flow glass micro-fermentors have previously been used, under aerobic conditions, for biofilm growth of *E. coli* and other microbial species (Ghigo, 2001; Valle et al., 2003; Beloin et al., 2004). The medium flow renews nutrients and removes planktonic cells, and it could mimic conditions in the gut lumen. Strain 630Δ*erm* was anaerobically grown in TYt medium whose flow was first set at a low rate to allow cell adhesion. Within the first 24–48 h, there was almost no visible growth except a few macro-colonies on vertical walls and a thin submersed biofilm at the fermentor bottom (**Supplementary Figure S2**). We could sometimes observe macro-aggregates slowly moving forward and backward in the liquid, indicating that their adhesion to glass was challenged by the medium flow. After 68–72 h, there were macro-colonies and submersed biofilms on walls and at the bottom of the fermentor (**Figure 1**). At 72 h, as biomass seemed not to have further increased, medium and gas flows were stopped. As soon as the biofilm biomass was recovered, most of it easily slided along vertical surfaces and fell down, even though macro-colonies could remain attached. *C. difficile* biofilm was nevertheless held together as a whole without dissociating even after having been vortexed. All these results indicate that, under these conditions, *C. difficile* is able to efficiently form macro-colonies and submersed biofilms characterized by inward cohesion despite loose adhesion to the surface. The micro-fermentor system thus allowed observing the development and the morphology of *C. difficile* biofilm at the macroscopic level (**Figure 1**). In previous studies using microtiter plates, only the biofilm biomass was quantified at the end of growth (Dapa et al., 2013; Soutourina et al., 2013; Pantaleon et al., 2015; Purcell et al., 2015; Chu et al., 2016). In one study using plastic culture flasks, macrocolonies and a submersed biofilm were formed at the flask bottom and the biofilm could easily be detached at the end of growth by gentle agitation and floated in the medium without dissociating (Dawson et al., 2012). In this study and ours, submersed *C. difficile* biofilms, albeit having grown in completely different systems and media *ex vivo*, share very similar development and properties, which could thus represent the hallmark of the same physiological biofilm.

### Genome-Wide Comparison of Gene Expression Between Biofilm and Planktonic Growth

In order to get insights into the genetic bases of biofilm formation by strain 630Δ*erm*, gene expression was compared between micro-fermenter biofilms and batch planktonic cultures, as previously described (Lazazzera, 2005; An and Parsek, 2007), including by our group (Beloin et al., 2004), as it is difficult to get rid of biofilm contamination in micro-fermenter cultures. Biofilms were grown as described above in continuously renewed TYt medium for 72 h (**Figure 1**), till an apparently intermediate, rather than very late, stage (**Supplementary Figure S2** and see above). For the planktonic reference, cells were grown in parallel in Falcon tubes in the same medium for 24 h. Considering that there are no ideal conditions for planktonic growth in such studies (Lazazzera, 2005; An and Parsek, 2007), and taking into account previous studies in *E. coli* (Beloin et al., 2004) and *B. subtilis* (Ren et al., 2004), planktonic cultures were grown for 24 h rather than 72 h to limit cell lysis and sporulation.

751 genes (20% of *C. difficile* genome) are differently expressed between biofilm and planktonic cells (**Supplementary Table S2**) and almost half of them are up-regulated (338 genes). Only 3 genes, *dccA*, *pilA_1_*, and *lcpB*, have previously been described to influence biofilm formation (Dapa et al., 2013; Soutourina et al., 2013; Purcell et al., 2015; Chu et al., 2016; Maldarelli et al., 2016). Many up-regulated genes are involved in translation and in metabolic pathways, including central metabolism while few down-regulated genes are involved in sporulation (**Supplementary Table S2**). All these results suggest that biofilm cells are metabolically active, in agreement with nutrients being supplied in abundance by continuously renewed medium in the micro-fermentors.

#### Transport and Metabolism

##### Sugar transport

**FIGURE 2:**
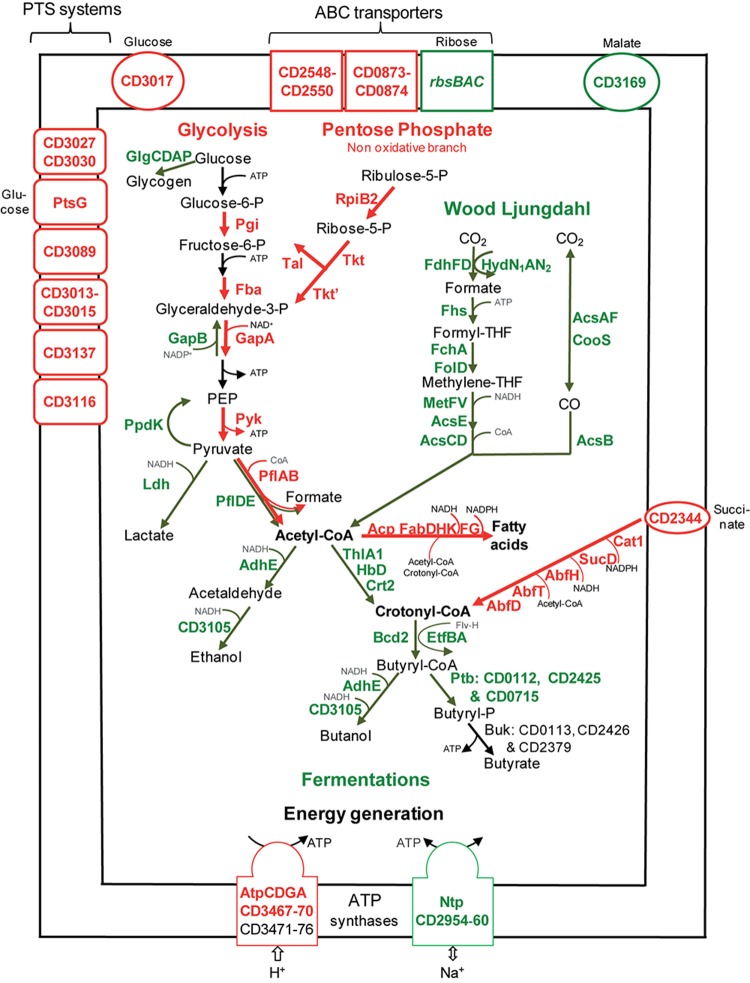
Sugar transport and metabolism in biofilms compared to planktonic cultures. A model for sugar transport and utilization pathways in biofilm compared to planktonic cells is proposed. It is based on the expression of genes involved in sugar transport and metabolism, and their variation between micro-fermentor biofilms (**Figure 1**) and planktonic cultures (**Supplementary Table S2**). The name/short identification number of proteins is indicated in green or red when their gene is down- or up-regulated during biofilm/planktonic growth, respectively (see **Supplementary Table S2** for protein activity/function). For simplicity, the number of molecules is not indicated in metabolic reactions (e.g., for glycolysis, each molecule of glucose leads to two molecules of glyceraldehyde 3-phosphate and all subsequent products). PEP, Phospho-Enol-Pyruvate; THF, TetraHydroFolate; CoA, Co-enzyme A; P, phosphate.

Sugar uptake seems active in biofilms (**Figure 2** and **Supplementary Table S2**). The most up-regulated genes (up to 17-fold) in biofilm compared to planktonic cells encode PTS systems: a putative glucose-maltose PTS (CD3027 and CD3030), the glucose specific PtsG, a putative mannose PTS (CD3013–CD3015) and the two β-glucoside specific BglF systems. PTS systems have previously been shown to be required for biofilm growth in a few species, e.g., *Streptococcus gordonii* (Loo et al., 2003). In *C. difficile* biofilms, genes encoding uptake systems of other families [ABC: CD2548–CD2550 and CD0873–CD0874 (Kovacs-Simon et al., 2014), and another family: CD3017], are also up-regulated.

##### Central carbon metabolism

Sugars enter the glycolytic pathway to produce energy and precursors of organic molecules (Richardson et al., 2015). Four genes (*pgi*, *fba*, *gapA*, and *pyk*) encoding essential glycolytic enzymes are up-regulated in biofilms, suggesting that glycolysis is active (**Figure 2**). Furthermore, it is presumably actively fuelled by the Pentose Phosphate Pathway non-oxidative branch, whose genes, *rpiB_2_*, *tkt*, *tkt,’* and *tal*, are up-regulated. On the contrary, genes involved in glycogen synthesis (*glgCDAP*) are less expressed in biofilm (**Figure 2**). Finally, pyruvate and ATP production seems to be prefered to storage in biofilm cells, suggesting that these cells could need metabolites and energy for biomass increase and/or matrix biosynthesis.

After glycolysis, pyruvate can be converted into acetyl-CoA by Pyruvate Formate Lyase (PFL). Among the three operons encoding a PFL complex, *pflBA* (*CD0759–CD078*) is up-regulated (up to fivefold) during biofilm growth, while *pflED* (*CD3283–CD3282*) is down-regulated (up to fivefold, **Figure 2**). This is also the case of all genes (*fdhDF-hydN_1_AN_2_* operon, *acsA*-*acsF*-*fhs*-*fchA-folD*-*metV*-*metF*-*lpdA-cooC*-*acsDCEB-acsV* operon and *cooS* gene, up to eightfold) of the Wood Ljungdahl pathway (WLP) involved in acetyl-CoA production from CO_2_ (Köpke et al., 2013; Schuchmann and Müller, 2014) (**Figure 2**). Therefore, to produce acetyl-CoA in micro-fermentor biofilms, under nutrient repletion conditions, sugar utilization is likely preferred to CO_2_ utilization (**Figure 2**). This could be due to the high energetic cost of WLP, which consumes ATP but allows autotrophic growth probably under depletion conditions (Schuchmann and Müller, 2014).

##### Sugar fermentations

**FIGURE 3:**
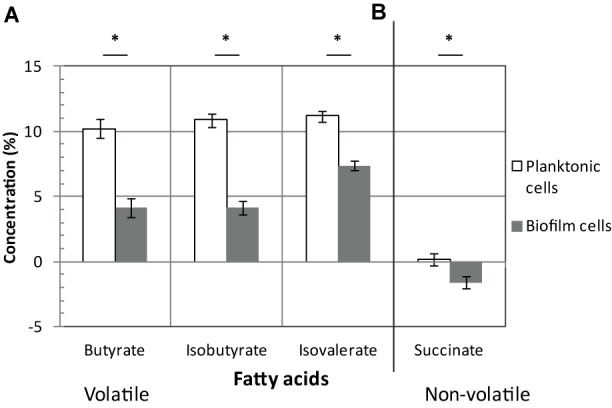
Fatty acids in biofilms/planktonic cultures. Strain 630Δ*erm* was grown either as biofilms in microfermentors (like in **Figure 1**) or as planktonic cultures in Falcon tubes. Supernatants of biofilms (in gray) and planktonic cultures (in white) were recovered and their composition in volatile **(A)** and non-volatile fatty acids **(B)** was determined. Relevant fatty acids, as inferred from our transcriptomic study, are shown. ^∗^ indicates a statistically significant difference (*p*-value < 0.05). Pyruvate, lactate and isocaproate concentrations were not statistically different between biofilms and planktonic cultures.

Once produced, pyruvate and acetyl-CoA are probably not fueling sugar fermentation pathways whose genes are strongly down-regulated (up to 10-fold) in biofilms (**Figure 2**), but rather alternative pathways (see below). Down-regulated genes encode: (i) Ldh, responsible for lactate production from pyruvate, (ii) AdhE and CD3105, both responsible for ethanol and butanol production from acetyl-CoA and butyryl-CoA, respectively, and (iii) enzymes needed for most steps of butyrate production from acetyl-CoA, ThlA1-HbD-Crt2-Bcd2-EtfAB (CD1054–CD1059) and Ptb (**Figure 2**). These data suggest that fermentation-end products could be reduced in biofilm. The supernatants of biofilms and planktonic cultures were therefore compared by gas phase chromatography. Although lactate and pyruvate levels did not significantly vary (data not shown), butyrate level decreased more than twofold in biofilms than in planktonic cultures (**Figure 3A**), in good agreement with the down-regulation of genes involved in its production (*CD1054–CD1059* operon and *ptb* genes, **Figure 2** and **Supplementary Table S2**). These data confirm a reallocation of carbon resources in biofilm compared to planktonic cells under our conditions.

##### Succinate utilization

In biofilm cells, the reductive utilization of succinate is active. Indeed, succinate, that is present in TYt medium, is consumed during biofilm growth (**Figure 3B**) and *CD2344–CD2338* operon, involved in its uptake (*CD2344*) and conversion into crotonyl-CoA (*cat1*, *sucD*, *abfH*, *abfT*, and *abfD*), is consistently up-regulated (up to fivefold; **Figure 2**). AbfH is an NADH-dependent enzyme that is over-expressed in biofilm cells, while Ldh, AdhE and putatively CD3105, are poorly expressed ones. AbfH therefore appears to be well suited to fulfill the high need for NAD^+^ regeneration due to active glycolysis during biofilm growth (**Figure 2**). A similar model has previously been proposed *in vivo*. Succinate, a metabolite produced by a commensal species, is able to promote *C. difficile* expansion in bi-colonized mice fed with a sugar-rich diet (Ferreyra et al., 2014). As *CD2344–CD2338* operon is up-regulated, succinate reduction has been proposed to promote the efficient use of dietary sugars *via* glycolysis (Ferreyra et al., 2014). Succinate reduction could therefore be necessary for redox balance during both *in vivo* and biofilm growth.

It is worth noting that, even though succinate reduction is able to fuel butyrate fermentation, as elegantly shown in planktonic cells *ex vivo* (Ferreyra et al., 2014), this is not the case in biofilm cells (**Figures 3A,B**). Consistently, *CD1054–CD1059* and *CD2344–CD2338* operons are, respectively, down and up-regulated in biofilm cells (**Figure 2**), and this is also the case under other growth conditions, *ex vivo* (Saujet et al., 2011; Pettit et al., 2014) and *in vivo* (Ferreyra et al., 2014). Moreover, *CD2344–CD2338* up-regulation can be observed either when the butyrate level is high (Ferreyra et al., 2014) or low (Pettit et al., 2014) (**Figures 2**, **3A**). The simplest model accounting for all these results is that depending on conditions, succinate can fuel butyrate fermentation or an alternative pathway, as it seems to be the case in biofilm (see below).

##### Energy generation

For energy generation, *C. difficile* is among the few bacterial species, including *Enterococcus hirae* (Murata et al., 2001), to have two bi-functional ATP synthase/ATPase complexes, the classical F-type named Atp and the V-type or Ntp. In *C. difficile* biofilm cells, the whole *ntp* operon (*CD2960–CD2954*) is strongly down-regulated (10- to 25-fold), whereas several essential *atp* genes are up-regulated. This suggests that biofilm and planktonic cells preferentially use Atp or Ntp, respectively. One possibility is that Ntp could mainly serve as an ATPase driving Na^+^ excretion as in *E. hirae* (Murata et al., 2001) and that its repression in biofilms could save ATP. Alternatively, Ntp could be an ATP synthase coupled to an ATP-consuming function like WLP, whose genes and *ntp* genes are co-expressed in the stationary phase of planktonic growth (Saujet et al., 2011) and negatively controlled by the same regulators (CD2214–CD2215, see below).

##### Nitrogen source transport and metabolism

**FIGURE 4:**
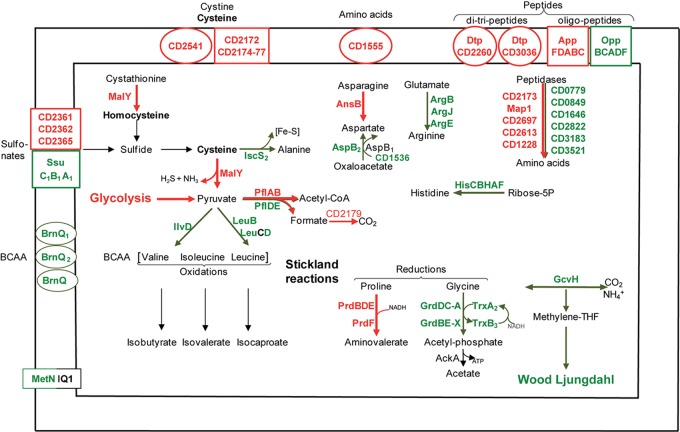
Amino acid transport and metabolism in biofilms compared to planktonic cultures. A model for amino acid transport and utilization pathways in biofilm compared to planktonic cells is proposed. It is based on the expression of genes involved in amino acid transport and metabolism, and drawn as in **Figure 2**. BCAA, Branched Chain Amino Acids; Fe-S, Iron-Sulfur cluster.

A biofilm grown in a micro-fermentor probably imports and requires nitrogen sources (**Figure 4**), as inferred from the up-regulation of several genes involved in their uptake and metabolism. *CD3036* and *CD2260* genes, which encode Dtp systems for di and tri-peptide uptake, and *app* genes (6- to 14-fold), which encodes one ABC system for oligopeptide uptake (Edwards et al., 2014) are up-regulated, while the homologous *opp* operon is down-regulated. Several peptidases (Map1-CD0092, CD1228, CD2173, CD2613, and CD2697) and one transporter (CD1555) are presumably overproduced (**Figure 4**), likely providing biofilm cells with amino acids. On the contrary, genes involved in the biosynthesis of histidine (*hisCBHAF*), branched chain amino acids (leucine: *leuBD*, isoleucine and valine: *ilvD*) and aspartate (*aspB-CD1339*), are down-regulated during biofilm growth (**Figure 4**). Thus, peptide uptake and degradation are likely to be favored over amino acid synthesis in biofilm, and this could rely on the peptide richness of the growth medium, TYt, which contains tryptone.

##### Cysteine and sulfur source transport and metabolism

Cysteine and other sulfur-containing compounds seem to be required in biofilms as genes involved in their uptake are up-regulated: *CD2172–CD2177* genes (10-fold) and *CD2541* gene, respectively, encodes an ABC system for cysteine or cystine and a symporter of cystine and sulfur compounds. Cysteine could serve as an important source of reducing agents, like H_2_S, and/or of low molecular weight thiols, like homocysteine (**Figure 4**). The most up-regulated gene during biofilm growth, *malY-CD3029* (20-fold), is an essential gene belonging to a PTS-encoding operon (*CD3027–CD3030*) (Dembek et al., 2015; Dubois et al., 2016). MalY, as a putative cystathionine β-lyase, could degrade cystathionine into homocysteine. As a demonstrated cysteine desulfhydrase *in vitro*, MalY could degrade cysteine while increasing the pyruvate pool and forming H_2_S (Dubois et al., 2016) (**Figure 4**). In *Lactobacillus reuteri*, one of the most up-regulated genes in an *in vivo* biofilm encodes a putative cystathionine γ-lyase displaying, like MalY, cysteine desulfhydrase activity *in vitro* (Frese et al., 2013). All these data suggest that in two gut inhabitant species, cysteine metabolism could contribute to biofilm cell fitness.

##### Stickland reactions

*C. difficile* can grow on amino acids as sole sources of carbon and nitrogen, using the coupled oxidation and reduction of amino acids called Stickland reactions (Bouillaut et al., 2013; Richardson et al., 2015). Both proline and glycine are efficient electron acceptors (Bouillaut et al., 2013) whose reduction pathways are inversely regulated in biofilm. Almost all *prd* genes encoding the proline reductase are induced, whereas all *grd* genes encoding the glycine reductase are down-regulated (up to 10-fold, **Figure 4**). Such an inverse regulation has previously been observed under several other growth conditions, *ex vivo* (Antunes et al., 2012; Bouillaut et al., 2013) and *in vivo* (Janoir et al., 2013). Therefore, proline seems to be often preferred over glycine as a Stickland acceptor, although only glycine reduction leads to ATP generation (**Figure 4**). Proline reduction has previously been proposed to regenerate NAD^+^ for active glycolysis in planktonic cells grown in the presence of glucose (Bouillaut et al., 2015), and this could also hold true in continuous-flow microfermentor biofilms under nutrient repletion conditions. Finally, all our results suggest that both proline and succinate reduction pathways could allow biofilm cells maintaining proper NAD^+^/NADH ratios.

The branched chain amino acids Isoleucine, Valine, Leucine are efficient Stickland electron donors (Bouillaut et al., 2013). However, as genes involved in their biosynthesis, *ilvD* and *leuBD*, are down-regulated during biofilm growth (**Figure 4**), they could be in limiting amounts for Stickland oxidation. In support to this hypothesis, the levels of isovalerate and isobutyrate, the main volatile fatty acids produced during the Stickland oxidation of isoleucine and valine (Elsden and Hilton, 1978) (**Figure 4**), decrease in the culture medium after biofilm compared to planktonic growth (**Figure 3A**). Proline reduction might thus be coupled to the oxidation of another amino acid or, alternatively, formate (Andreesen et al., 1999). Interestingly, *CD2179* gene encoding a putative formate dehydrogenase (Köpke et al., 2013) is up-regulated in biofilm (**Figure 4**).

Many genes differentially expressed in biofilm/planktonic cells appear to be involved in metabolism and transport, as previously observed in similar studies (Lazazzera, 2005). This raises the possibility that some of the variations in gene expression observed here, between biofilms grown under a medium continuous-flow and planktonic cultures in the late stationary phase, could be correlated to differences in their growth conditions rather than in their lifestyles (Lazazzera, 2005; An and Parsek, 2007). To assess the impact of growth phase on the biofilm transcriptome, we took advantage of our previous study of gene expression according to the (exponential or stationary) phase of planktonic growth in TY medium, close to TYt (Saujet et al., 2011).

#### Assessment of Growth Phase Impact on the Transcriptome

The biofilm/planktonic and growth phase transcriptomes (Saujet et al., 2011) were compared (**Supplementary Figure S3A**). This revealed an overlap, with most common genes (around one third of the total in biofilm/planktonic cells) displaying the same variation of expression (up- or down-regulation) in both transcriptomes (Spearman correlation of 0.69 and *p*-value < 0.05). Most of them are involved in three main functional categories: (i) metabolism, especially sugar (pyruvate conversion, butyrate fermentation, WLP, glycogenesis, succinate utilization) and amino acid metabolism, (ii) transport and (iii) translation (genes encoding ribosomal proteins; **Supplementary Figure S3A**). They represent 70% of ribosomal genes and 50% of transport and (sugar and amino acid) metabolism genes varying in biofilm/planktonic cells (**Supplementary Figure S3A**). Interestingly, many of these metabolism and transport genes are also regulated in the same direction in the *spo0A* mutant/630Δ*erm* strain (Pettit et al., 2014) (**Supplementary Figure S3B**), so that Spo0A could account for the control of their expression in stationary phase planktonic cells. In total, in our biofilm/planktonic experiment, growth phase differences seem to have an intermediate impact on gene expression, especially in the case of metabolism, transport and translation genes. Noteworthy, the magnitude of gene expression variations can be very different between the transcriptomes (e.g., for *malY* and *ntp* genes, >20-fold in biofilm/planktonic cells against 2- or 3-fold according to the phase), further suggesting that differences in growth phase do not account for all expression differences between biofilm and planktonic cells. Finally, in marked contrast to metabolism, transport and translation genes, the expression of the wide majority (almost 80%) of genes involved in cell surface biogenesis is independent of the growth phase (**Supplementary Figure S3A**). These genes are therefore good candidates of lifestyle specific genes, as previously noticed in similar studies (Lazazzera, 2005; An and Parsek, 2007).

#### Toxin Production

Toxins (TcdA and TcdB) are the main disease-causing factors of *C. difficile*. *tcdA* is the most down-regulated gene (25-fold) during biofilm growth. The specific down-regulation of *tcdA* and not of *tcdB*, which has already been observed in previous transcriptomes, has been proposed to rely on the low expression level of *tcdB* (Saujet et al., 2011; Pettit et al., 2014). *tcdA* repression in biofilm is consistent with the well-known coordinated expression of toxin and metabolism genes (Bouillaut et al., 2015; Richardson et al., 2015). Indeed, in exponentially growing planktonic cells like in biofilm cells, toxin synthesis is co-repressed with butyrate and lactate fermentations, glycogen formation, WLP, Ntp synthesis and amino acid biosynthesis (Karlsson et al., 2008; Dineen et al., 2010; Saujet et al., 2011). And toxin synthesis and glycine reduction are co-repressed, while proline reduction is induced, both in biofilm cells and in planktonic cells grown in the presence of either proline (Bouillaut et al., 2013, 2015) or glucose (Antunes et al., 2012). In biofilm cells, the coordinated expression pattern of toxin and metabolism genes (**Supplementary Table S2**) could result from nutrient repletion conditions in a micro-fermentor where the medium is continuously renewed (Dineen et al., 2010). In addition, the decrease of endogenously produced butyrate (**Figure 3A**) is also expected to decrease toxin gene expression (Karlsson et al., 2000). These data suggest that the primary function of the biofilm characterized here could be colonization rather than toxin production. *C. difficile* biofilms producing little toxins could be physiological *in vivo* in the gut under certain conditions, e.g., during an early asymptomatic colonization and/or carriage step, possibly before a later toxin production step (Abt et al., 2016; Smits et al., 2016).

#### Cell Surface Biogenesis

##### Membrane biogenesis

*De novo* membrane biogenesis seems to be active in biofilm. Two essential genes involved in phospholipid metabolism, *pslX* and *cdsA*, are up-regulated. This is also the case of the two *acpP* copies encoding Acyl Carrier Proteins (ACP) and almost all essential *fab* genes (*fabHKDG* and *fabF*) involved in fatty acid synthesis (**Figure 2**). This synthesis requires precursors and co-factors (Zhang and Rock, 2008) presumably efficiently produced in biofilm cells (**Figure 2**). Acetyl-CoA and ATP required at the first step are produced during active glycolysis and by the Atp complex (**Figure 2**). Acetyl-CoA and other acyl-CoA, like crotonyl-CoA (Zhang and Rock, 2008) are required for fatty acid elongation. This suggests a possible fate for the product of succinate reduction pathway (**Figure 2**), also involved, *via* its AbfH and SucD enzymes, in regenerating NADH and NADPH co-factors necessary for FabK and FabG activity, respectively (**Figure 2**). It has previously been shown that fatty acid biosynthesis is able to promote biofilm formation in a few species, e.g., in *B. subtilis* (Pedrido et al., 2013).

##### Peptidoglycan biogenesis

Some early steps of peptidoglycan biogenesis seem to be active and favored over late steps in biofilm. First, several genes involved in the biosynthesis of amino-sugars are up-regulated. This is the case of *nan* genes (four–sixfold), required for the uptake and conversion of sialic acid, a mucus component, into *N*-acetyl-glucosamine-6-phosphate. The ability to use sialic acid represent an advantage for *in vivo* growth and colonization (Ng et al., 2013). Indeed, *C. difficile* has recently been found to be present in and over the mucus layer in infected mice: either within a multi-species community in the mucus outer layer of conventional mice (Semenyuk et al., 2015), or as a 3D biofilm-like structure at the mucus surface in mono-associated mice (Soavelomandroso et al., 2017). Other up-regulated genes required for amino-sugar biosynthesis include the essential *glmSM* genes, involved in the production of D-glucosamine-1-phosphate, and *glmM* is required for biofilm formation in a few species, e.g., in *S. gordonii* (Shimazu et al., 2008). Second, *C. difficile* genes involved in the use of amino-sugars and their conversion into cytoplasmic peptidoglycan precursors are also up-regulated: these are three essential *mur* genes (*murA*, *murC*, and *murE*), involved in the biosynthesis of UDP-N-acetyl-muramic acid-pentapeptide, and the essential *ddl*-*CD1408* gene encoding a D-alanine-D-alanine ligase. The Mur machinery might interact with the cell shape control protein MreB as in other species and one essential *mreB* gene (*CD1145*) is up-regulated in biofilm.

In contrast to these genes, several genes involved in late steps of peptidoglycan biogenesis and encoding peptidoglycan hydrolases are down-regulated. They include *acd* encoding a *N*-acetyl-glucosaminidase (Dhalluin et al., 2005), *CD0784* and *cwp17* encoding putative *N*-acetyl-muramoyl-L-alanine amidases, *cwp20* encoding a protein with a β-lactamase domain, and one *dacF* gene copy (*CD1291*) encoding a carboxypeptidase, while the other copy (*CD2498*) is up-regulated. Finally, all our expression data suggest that the envelope and wall of biofilm cells is probably remodeled compared to that of planktonic cells.

##### Wall glycopolymers

The biosynthesis of wall polysaccharides, the atypical teichoic acid PSII and the lipoteichoic acid PSIII (Ganeshapillai et al., 2008; Reid et al., 2012; Willing et al., 2015; Chu et al., 2016) seems only marginally affected in biofilm cells as only four genes of the glycopolymer synthesis locus are differentially expressed (**Supplementary Table S2**). Up-regulated genes include an essential gene encoding a putative glucosyl transferase (*CD2778*) and *lcpB* involved in PSII deposition at the cell surface (Chu et al., 2016), suggesting that PSII might be efficiently anchored at the cell surface in microfermentor biofilms. Interestingly, *dltABCD* genes that are required for the incorporation of ester-linked D-alanine into cell wall extracts (McBride and Sonenshein, 2011), are up-regulated during biofilm growth. PSII and PSIII teichoic acids, which contain anionic phosphodiester linkages, have been proposed to be suitable substrates for Dlt enzymes (Reid et al., 2012). *dlt* genes have previously been shown to promote biofilm formation *ex vivo* in *Staphylococcus aureus* (Gross et al., 2001) and to play a role *in vivo* in both biofilm formation and colonization in the gut resident *Lb. reuteri* (Walter et al., 2007). Finally, as *C. difficile* *dlt* genes are involved in the resistance of planktonic cells to cationic antimicrobial peptides (McBride and Sonenshein, 2011), it is tempting to speculate that the D-alanylation of wall polysaccharides might protect biofilm cells from the gut cationic anti-microbial peptides *in vivo*.

##### Protein translocation out of the cytoplasm

**FIGURE 5:**
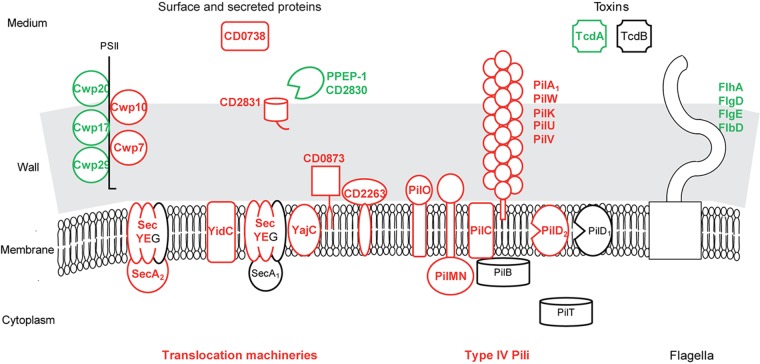
Protein export in biofilms compared to planktonic cultures. A model for protein export pathways and exported protein production in biofilm compared to planktonic cells is proposed. It is based on the expression of genes involved in protein export or encoding exported proteins, and drawn as in **Figure 2**. PSII, surface polysaccharide II and atypical teichoic acid.

Protein translocation seems to be active in biofilms (**Figure 5** and **Supplementary Table S3**). Indeed, several transmembrane channel subunits of the general Sec translocon, the essential SecY-PrlA and SecE proteins and the accessory YajC-CD2801 protein are presumably over-produced. This is also the case of (i) YidC-OxaA_1_-SpoIIIJ-CD3678, cooperating with Sec translocon to integrate proteins into the cytoplasmic membrane, (ii) CD2263-PrsA, an extra-cellular folding factor, and (iii) SecA_2_, the essential motor ATPase dedicated to a subset of proteins bearing or not an export-signal (Fagan and Fairweather, 2011) (**Figure 5**). Several genes involved in protein translocation have previously been found to be up-regulated during biofilm growth and/or required for it. In *S. aureus*, *secE* gene is up-regulated in an early biofilm compared to a planktonic culture (Resch et al., 2005). In *Streptococcus mutans*, YidC protein could promote biofilm formation (Palmer et al., 2012). *secA_2_* gene is known to promote biofilm formation in two species, in *Listeria monocytogenes* during *ex vivo* growth at 37°C (Renier et al., 2014), and in *Lb. reuteri* during *in vivo* colonization of the forestomach epithelium, which also leads to *secA_2_* gene up-regulation (Frese et al., 2013). Finally, our results suggest that the general and dedicated (Sec, Sec-YidC or Sec-SecA_2_) machineries could actively translocate proteins bearing or not an export signal out of the cytoplasm of biofilm cells.

Among the numerous proteins probably over-produced in biofilm cells, only the ones predicted to be exported out of the cytoplasmic membrane (bearing an export-signal different from a transmembrane domain) are considered in the following (**Figure 5** and **Supplementary Table S3**). CD0873, a sugar-binding lipoprotein, is involved in adhesion to Caco-2 cells (Kovacs-Simon et al., 2014) whereas other lipoproteins are thought to be exclusively involved in transport (**Supplementary Table S3**). CD2831, a LPXTG protein covalently anchored to the wall by SrtB sortase, is a collagen-binding protein *in vitro* and a possible adhesin (Hensbergen et al., 2015). It should remain anchored to the surface of biofilm cells, as PPEP-1/CD2830, the extra-cellular metallo-protease responsible for its release into the medium (Cafardi et al., 2013; Hensbergen et al., 2014, 2015; Peltier et al., 2015) is presumably less produced in biofilms (**Figure 5**). Two cell wall proteins, which are probably exported by SecA_2_-Sec and non-covalently anchored to PSII (Fagan and Fairweather, 2011; Willing et al., 2015), Cwp10 and the essential Cwp7 protein, are of unknown function, as a putative secreted protein, CD0738 (**Figure 5**). These export-signal bearing proteins represent candidates for extra-cellular biofilm formation factors and/or matrix components, which might also comprise proteins devoid of an export-signal and translocated by the dedicated SecA_2_ pathway.

##### Organelles

Type IV pili, which are known to mediate gliding and twitching motility, host cell adherence and biofilm development (Melville and Craig, 2013), seem to be produced at the biofilm cell surface in contrast to flagella (**Supplementary Table S3** and **Figure 5**). Most primary *pil* genes (*CD3513–CD3503*) (Bordeleau et al., 2015) and *pilW*-*CD2305* gene (Melville and Craig, 2013) are up-regulated in biofilms. They encode an assembly machinery (core membrane protein PilC, accessory membrane proteins PilMN and PilO and the pre-pilin peptidase PilD) for both minor (PilVUK) and major pilins (PilA_1_*-*CD3513 and PilW-CD2305) (Melville and Craig, 2013). In strain R20291, *pilA_1_* gene, alone or together with other *pil* genes, has previously been found to be up-regulated in biofilms and to contribute to cell clumping in a peculiar, early biofilm grown on glass coverslips (Purcell et al., 2015; Maldarelli et al., 2016). In *Pseudomonas aeruginosa*, Type IV pili are involved, possibly *via* twitching mobility allowing cell migration, in the formation of either microcolonies adhesive to the surface at an early step of biofilm formation, or caps in mushroom-like structures at a late step (van Gestel et al., 2015).

Finally, many genes differentially expressed in biofilm/planktonic cells are involved in the biogenesis of the cell surface and the expression of most of them is independent from the growth phase. They therefore represent good candidates to be involved in biofilm formation (Lazazzera, 2005).

#### Regulation and Signaling

Several genes encoding transcriptional regulators are differentially expressed during biofilm/planktonic growth, in agreement with the wide reprogramming of gene expression, but little is known about their function (**Supplementary Table S2**). A two component system regulator that is presumably over-produced in biofilms, CD3267, is encoded by a c-di-GMP-controlled gene (Soutourina et al., 2013). Several genes differentially expressed in biofilms encode putative signaling proteins, di-guanylate cyclases (DGCs) bearing a GGDEF domain, and phosphodiesterases (PDEs) bearing both EAL and GGDEF domains (Bordeleau et al., 2011). Genes encoding three putative DGCs, CD2384, CD2385, and DccA-CD1420, and two putative PDEs, CD2134 and CD1421, are up-regulated, while genes encoding a DGC, CD1185, and a PDE, CD1840, are down-regulated (**Supplementary Table S2**). Evidences supporting the activity of DccA, CD1185, CD1421, CD2134, and CD1840, could be obtained in a heterologous host (Bordeleau et al., 2011). DccA, an active DGC *in vitro*, increases biofilm formation and cell aggregation and decreases motility when over-produced in *C. difficile* (Purcell et al., 2012, 2015; Soutourina et al., 2013; Peltier et al., 2015). The expression of DGC- and PDE-encoding genes suggests a tight and complex regulation of the c-di-GMP level in biofilm cells. The coordinated expression pattern of several c-di-GMP-dependent genes, the down-regulation of *CD2830* together with the up-regulation of *CD2831*, *pilA_1_* and *CD3267*, suggests that c-di-GMP level could be high in biofilm cells, although not all c-di-GMP-dependent genes are differentially expressed (Purcell et al., 2012, 2015; Soutourina et al., 2013; Peltier et al., 2015). Finally, our comparison of whole gene expression between biofilm and planktonic cells suggest that the biofilm lifestyle could involve a wide reprogramming of cell metabolism and surface properties.

### CD2214, a SinR-Like Regulator, and CD2215 Control Many Genes Differentially Expressed in Biofilms

#### CD2214–CD2215 Regulon in Strain 630Δ*erm*

We previously identified *C. difficile* putative DNA-binding protein CD2214 as a SinR-like protein (Saujet et al., 2011): it is the best homolog of SinR, the *B. subtilis* repressor of biofilm formation, sporulation, motility and autolysis (Cairns et al., 2014), even though their similarity is low and essentially restricted to the HTH domain. We decided to study CD2214 for its putative role on gene expression and biofilm formation. During the course of this study, *CD2214* and *CD2215* genes, organized as an operon (Soutourina et al., 2013; Girinathan et al., 2018), have independently been studied. CD2215 protein is, like CD2214 but to a lesser extent, homologous to *B. subtilis* SinR. CD2214 and CD2215 are, in an even weaker way, related to *B. subtilis* SinI, the SinR anti-repressor (Edwards et al., 2014; Nawrocki et al., 2016; Girinathan et al., 2018). In strain 630Δ*erm*, *CD2214* and *CD2215* have been named *sinR* and *sinI* and their expression has been studied, notably in response to the growth phase (Edwards et al., 2014; Nawrocki et al., 2016). In strains R20291 and JIR8094, *CD2214* and *CD2215* genes have been named *sinR* and *sinR’* and investigated for their role in diverse phenotypes except biofilm formation (Girinathan et al., 2018). Both *CD2214* and *CD2215* have a pleiotropic effect on gene expression and are needed to fully inhibit autolysis. The two genes independently control both sporulation and motility in opposite ways: *CD2215* gene alone, like *B. subtilis sinR*, inhibits them, while *CD2214* gene positively controls them. Finally, *in vitro*, CD2215 interacts with CD2214 and impedes its binding to their *codY* target promoter, thus to some extent recalling *B. subtilis* SlrR, the SinR co-repressor of motility and autolysis genes, which is able to inhibit SinR binding to matrix genes (Girinathan et al., 2018).

Here, *CD2214* was inactivated in strain 630Δ*erm* by Clostron insertion, leading to a polar effect on *CD2215* expression, and the resulting mutant is hereafter referred to as *CD2214–CD2215* mutant. To study CD2214–CD2215 role on gene regulation, total gene expression was compared between the parental and mutant strains after planktonic growth for 7 h in TY medium. The CD2214–CD2215 regulon in strain 630Δ*erm* represents 3% of the genome (**Supplementary Table S4**). It shows a relatively limited overlap with those recently determined in strains R20291 and JIR8094, after growth to the stationary phase (12 h) (Girinathan et al., 2018). In our study in particular, in contrast to the previous one (Girinathan et al., 2018), very few sporulation genes form part of the CD2214–CD2215 regulon, consistent with growth to the exponential phase (7 h). Moreover, many genes are controlled by CD2214–CD2215 in opposite ways in strain 630Δ*erm* (our study) and in the two other strains (Girinathan et al., 2018) (**Supplementary Table S5**), probably reflecting differences in strains (Collery et al., 2017) and growth conditions between the two studies.

#### Overlap Between CD2214–CD2215 Regulon and the Biofilm/Planktonic Transcriptome

**FIGURE 6:**
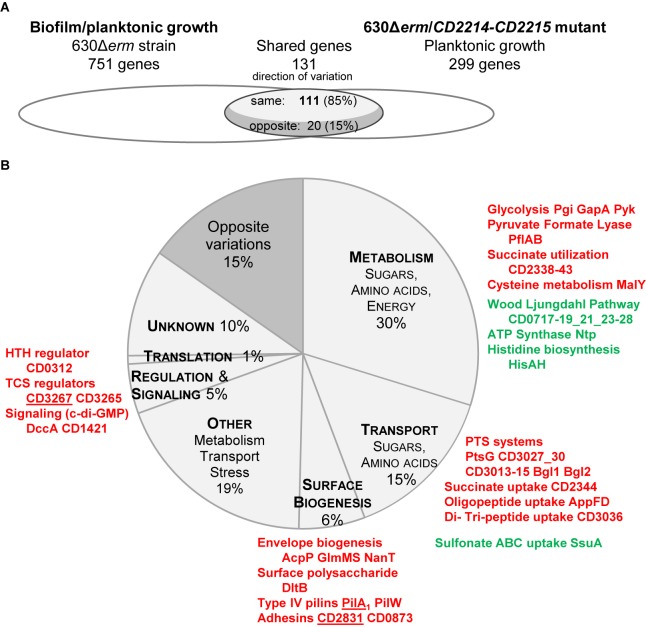
Comparison between CD2214–CD2215 regulon and the set of genes differentially expressed in biofilm/planktonic growth. **(A)** Overlap. Transcriptomes are drawn as elipses: the set of genes differentially expressed during biofilm/planktonic growth (**Supplementary Table S2**) is on the left and the set of genes differentially expressed in strain 630Δ*erm*/*CD2214–CD2215* mutant (**Supplementary Table S4**) is on the right. The number of shared genes (whose expression varies in both transcriptomes) is indicated above the overlap region. As shared genes can vary in the same direction in the two transcriptomes or in opposite directions, the overlap region is divided into two parts. The number of shared genes whose expression varies in opposite directions is indicated in the dark gray part, while the number of genes whose expression varies in the same direction is in the light gray part. **(B)** Functions. Genes common to the two transcriptomes are shown as a pie chart. The main functional categories of genes regulated in the same direction in the two transcriptomes appear in capital letters in light gray slices. A unique dark gray slice is shown for all genes regulated in opposite directions in the two transcriptomes, whatever the functional category they belong to. The function and the product of main shared genes (designated by their name or short identification number) are indicated beside the pie chart. Protein names/short identification numbers are in green or red depending on whether their genes are, respectively, down- or up-regulated in both transcriptomes. Proteins whose genes are controlled by a c-di-GMP riboswitch (Soutourina et al., 2013) are underlined.

In strain 630Δ*erm*, we noticed that CD2214–CD2215 regulon significantly overlaps the set of genes differentially expressed during biofilm/planktonic growth (**Figure 6**). The two transcriptomes share 131 genes, representing 44% of the CD2214–CD2215 regulon and 17% of genes differentially expressed in biofilm. Importantly, the regulation by CD2214–CD2215 and the variation of expression in biofilm are correlated (Spearman correlation factor of 0.67, *p*-value < 0.05). Among shared genes, most genes up- (down-)regulated in biofilms are positively (negatively) controlled by CD2214–CD2215 (85%, **Figure 6A**), suggesting that CD2214–CD2215 regulators could, directly or not, regulate almost 15% of all genes differentially expressed in biofilms.

Shared genes are assigned to several functional categories, with a high proportion involved in sugar uptake and carbon metabolism (**Figure 6B** and **Supplementary Table S4**). The operons/genes positively controlled by CD2214–CD2215 and up-regulated in biofilm encode: five PTS systems (PtsG, CD3027 and CD3030, CD3013–CD3015, the two Bgl), three glycolytic enzymes (Pgi, GapA, and Pyk), PflAB and all proteins necessary for succinate uptake and utilization (CD2344–CD2338). Most WLP genes and all *ntp* genes are negatively controlled by CD2214–CD2215 and down-regulated in biofilm (**Figure 6B** and **Supplementary Table S4**). CD2214–CD2215 proteins could also be responsible for the regulation of a few genes involved in cysteine metabolism (*malY* gene), histidine biosynthesis (*hisAH*), oligo-peptide, di- and tri-peptide and sulfonate uptake (*appFD*, *CD3036*, and *ssuA* genes) and cell envelope biogenesis (*acpP, glmMS*, *nanT*, and *dltB*, **Figure 6B** and **Supplementary Table S4**). In conclusion, in contrast to *B. subtilis* SinR (Cairns et al., 2014), CD2214–CD2215 proteins play a rather pleiotropic role in controlling the expression of metabolism and transport genes in strain 630Δ*erm*. Noteworthy, the control of many of these genes (*CD3027* and *CD3030*, *CD3013–CD3015*, *pgi*, *pflAB*, *CD2344–CD2338*, WLP genes, *ntp*, *malY*, *CD3036*, *ssuA*, *acpP, glmMS*, and *dltB* genes) by CD2214–CD2215 proteins could account for their regulation in early exponential/stationary phase planktonic cultures (**Supplementary Figure S3A**) (Saujet et al., 2011). Several of these genes (*pflAB*, *CD2344–CD2338*, *ntpAI*, *CD3036, ssuA*, and *dltB* genes) have also been shown to be expressed in the opposite direction under the control of Spo0A (**Supplementary Figure S3B**) (Pettit et al., 2014). All these data are consistent, as in strain 630Δ*erm*, *CD2214* and *CD2215* genes have been shown to be negatively controlled by Spo0A (Pettit et al., 2014). All our and previous data indicate that CD2214–CD2215 proteins could contribute to regulate cell metabolism and transport during the transition between the exponential and stationary phase of growth.

Yet, CD2214–CD2215 proteins also positively control other genes up-regulated in biofilms that are not regulated in response to the planktonic growth phase. They belong to two functional categories. (i) Several encode surface proteins: CD2831 and CD0873 adhesins, and PilA_1_ and PilW major Type IV pilins (**Figure 6B** and **Supplementary Table S4**). Two of them, CD2831 and PilA_1_, have been proposed to be involved in biofilm formation upon DccA over-production (Soutourina et al., 2013). (ii) Other encode regulatory and signaling proteins: CD3265 and CD3267 are two response regulators, and DccA and CD1421, respectively, are a DGC involved in c-di-GMP synthesis and a putative PDE possibly involved in c-di-GMP degradation (**Figure 6B** and **Supplementary Table S4**). Noteworthy, three of these genes: *CD2831*, *pilA_1_* and *CD3267*, are induced by c-di-GMP, through its binding to a specific type II riboswitch (Soutourina et al., 2013). They might therefore be indirectly controlled by CD2214–CD2215 *via* up-regulated *dccA* and a consecutive increase of c-di-GMP (Bordeleau et al., 2011; Soutourina et al., 2013) (**Figure 6B**). Finally, all our data suggest that, in strain 630Δ*erm* grown under our conditions, as planktonic cultures or biofilms, CD2214–CD2215 could control regulatory and c-di-GMP signaling pathways, together with the production of surface proteins, including two c-di-GMP-dependent ones previously proposed to be involved in biofilm formation.

### Genetic Analysis of Biofilm Formation

#### Ability of Mutants to Form Biofilms in Micro-Fermentors

To get insights about the mechanisms underlying biofilm formation, we decided to study a few genes identified by our convergent transcriptomic approaches for their ability to form a biofilm in a micro-fermentor. *pilA_1_* and *CD2831* genes are up-regulated in biofilm/planktonic cells and positively controlled by CD2214–CD2215 (**Figure 6**) and by c-di-GMP, and the surface proteins they encode have been proposed to mediate the c-di-GMP-dependent formation of biofilms (Soutourina et al., 2013). *pilA_1_* and *CD2831* genes were inactivated by Clostron mutagenesis. The *CD2214–CD2215* mutant was included in our study as it affects the expression of genes apparently specifically expressed in biofilms, including *pilA_1_* and *CD2831* (**Figure 6**). Two other genes up-regulated in biofilm/planktonic cells and positively controlled by CD2214–CD2215 failed to be inactivated: *malY*, the most up-regulated gene in biofilm cells, which has independently been shown to be essential during the course of this study (Dembek et al., 2015; Dubois et al., 2016), and, for an unknown reason, *pilW*, which encodes another major Type IV pilin.

*CD2214–CD2215*, *pilA_1_* and *CD2831* mutants were inoculated in micro-fermentors to test their ability to grow as a biofilm. All three were, like the parental strain, able to form macro-colonies and submersed biofilms (**Supplementary Figure S4**), showing that CD2214–CD2215 regulators, PilA_1_ pilin and CD2831 adhesin are dispensable for biofilm formation in micro-fermentors. Consistent with our results, the *pilA_1_* mutants of strain 630Δ*erm* and strain R20291 have previously been found to be dispensable for biofilm formation, respectively, in tissue culture plates (Purcell et al., 2015) and on glass coverslips although cell clumping was slightly affected in the latter, early biofilm (Maldarelli et al., 2016). As our mutants of strain 630Δ*erm* might affect some biofilm properties that are not detectable at the macroscopic level, we decided to study their phenotype at the microscopic level.

#### Microscopic Study of the Parental Strain Biofilm

##### Biofilm structure after disruption

**FIGURE 7:**
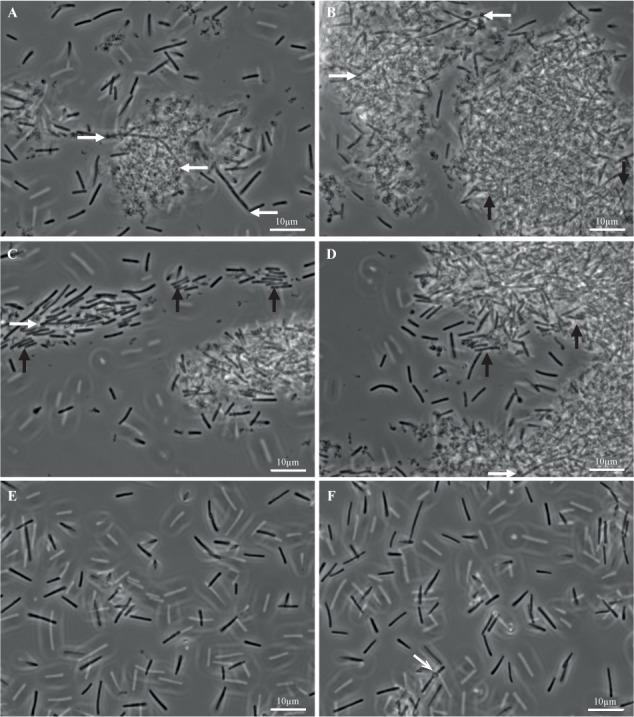
Biofilm and planktonic cells of strain 630Δ*erm*. Biofilms **(A–D)** and planktonic cultures **(E,F)** of strain 630Δ*erm* were grown in parallel in TYt medium, respectively, for 48 h in 24-well polystyrene micro-titer plates and for 24 h in Falcon tubes. After having been recovered, fixed and washed, biofilm and planktonic cells were observed by Transmitted Light Microscopy. Representative images are shown, with a white bar indicating the scale (10 μm). In biofilm **(A–D)**, (i) elongated rods (∼20–30 μm) are shown by white horizontal arrows, and (ii) cells aligned side by side along the width and tightly packed into micro-aggregates are indicated by black vertical arrows. In a planktonic culture **(F)**, a refracting spore is indicated by a gray oblique arrow.

In order to get insights into the microscopic structure of the biofilm, we compared biofilm and planktonic cells grown in the same TYt medium. Strain 630Δ*erm* was grown in 24-well polystyrene micro-titer plates for 48 h. After growth, biofilms were found to be loosely attached to the surface, as the ones grown in microfermentors. Planktonic cultures, like planktonic controls for transcriptomics, were grown for 24 h in Falcon tubes. After growth, both biofilm and planktonic cultures were recovered, fixed, washed and observed by Transmission Light Microscopy (**Figure 7**).

Planktonic cultures (**Figures 7E,F**) contain, as expected, individual vegetative cells (∼3–10 μm), with very few refracting spores (**Figure 7F**). Biofilms appear completely different (**Figures 7A–D**). Most of the biomass is present in dense and apparently aggregated structures of variable forms and sizes (e.g., ∼10 μm and ∼25–30 μm long in **Figures 7A,B**, respectively, but also smaller and larger ones, including ones covering the entire field, i.e., >∼100 μm long; data not shown). They are made of rod-shaped cells embedded into a polymorphic material. This material, which could contain cell debris, is reminiscent of a matrix as defined in pioneering biofilm studies, i.e., sticking cells together into a multi-cellular community. This biofilm highly ressembles the *E. coli* biofilm after growth in micro-fermentor and disruption (Ghigo, 2001). Isolated cells, which could have been released during biofilm treatment, are easier to observe than cells embedded into the polymorphic material. Some of them, sometimes protruding from large structures (**Figures 7A–D**, white arrows), are elongated (up to ∼20–25 μm). Other cells are tightly packed and aligned side by side along the width, apparently forming micro-aggregates (**Figures 7A–D**, black arrows). Barely no refracting spore could be observed (data not shown), suggesting that the 48 h-old micro-titer plate biofilm is at a mid, rather than late, stage of growth.

Finally, *C. difficile* cells grown as biofilms in TYt medium in micro-titer plates are shown to be embedded into a polymorphic material, thus forming large aggregated communities. Noteworthy, in *C. difficile* grown *in vivo*, bacterial aggregated structures of similar sizes have been observed at the epithelium surface of infected animals. In infected conventional hamsters and mice, rod-shaped bacteria are present as large mats/micro-colonies (∼20–30 μm long) at the surface of damaged tissue (Buckley et al., 2011; Lawley et al., 2012). In mono-associated mice, numerous 3D aggregated biofilm-like structures of comparable sizes (up to ∼50 μm long) can be observed inside and outside the mucus in the caecum (Soavelomandroso et al., 2017). These observations suggest that the biofilm structures evidenced here after *ex vivo* growth in TYt medium could be relevant *in vivo*, in the gut.

##### Architecture of the intact biofilm

In order to get insights into the biofilm microscopic architecture, the intact biofilm of strain 630Δ*erm* grown in TYt medium was observed *in situ* in 96 well polystyrene micro-titer plates using a non-invasive preparation and observation method. In order to mimic, in these micro-titer plates, the conditions prevailing in micro-fermentors notably at an early step, adhesive cells were used as starters for biofilm growth in freshly added TYt medium. After 48 h, biofilms were confirmed to be loosely attached to the surface, as the ones grown in microfermentors or in 24 well micro-titer plates. Intact biofilms were directly stained using a Live Dead kit under anaerobiosis and observed by Confocal Laser Scanning Microscopy (Bridier et al., 2010).

**FIGURE 8:**
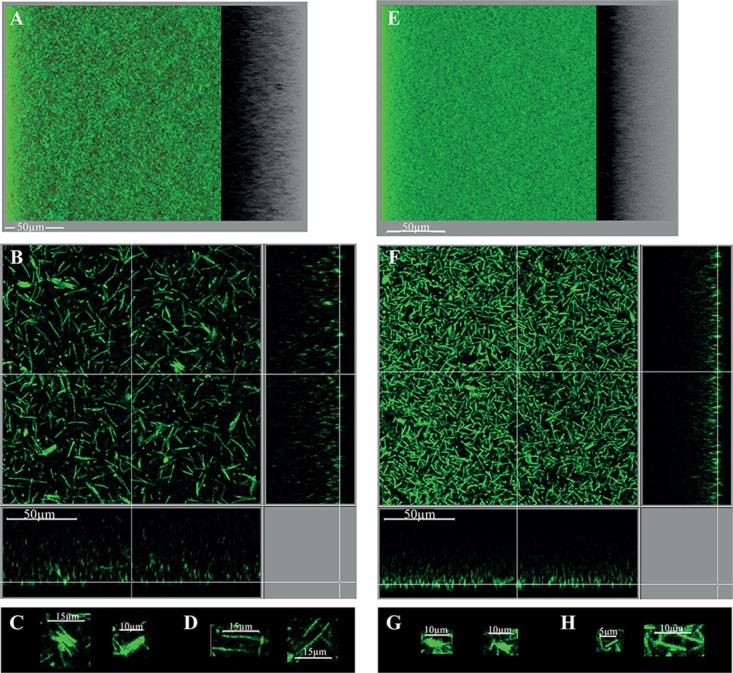
Intact biofilm architecture of the parental strain and *CD2214–CD2215* mutant. The biofilms of the parental strain 630Δ*erm* **(A–D)** and its *CD2214–CD2215* mutant **(E–H)** are shown. They were grown for 48h in 96-well polystyrene micro-titer plates, in TYt medium freshly added onto adhesive starter cells (**Supplementary Figure S5**). After live dead staining of intact biofilms directly in the micro-titer plates, their microscopic architecture was observed *in situ* by CLSM. Images representative of three independent experiments (each using three clones) are shown. Raw confocal z-stacks were treated using IMARIS software. This allowed obtaining both a 3D projection upside view, with its shadow on the right **(A,E)**, and a section view close to the surface **(B,F)**, in which the white bar indicates the scale (50 μm). For the section view of each strain, magnifications of micro-aggregated forms **(C,G)** and rods **(D,H)** are also provided, with the corresponding scale bar in white (5, 10, or 15 μm as indicated). After data recovery, biovolume, maximum coverage, mean thickness and biovolume/surface ratio were quantified and a statistical analysis was performed (see **Supplementary Figure S6**).

Starting from a discrete number of adhesive cells in each field (**Supplementary Figure S5**), a structured three-dimension biofilm is present at the end of growth (**Figures 8A,B**). Almost all staining is due to the green DNA-dye, and almost none to the red dye specific for cells with a damaged membrane, indicating that the wide majority of cells are alive. The biofilm is high, around 50 μm thick, and sparse: stained cells do not cover the polystyrene surface and are far from each other in the biofilm height (**Figure 8B**). The biofilm sparseness and height could conceivably contribute to its loose adhesion to the surface and fragile attachment in either micro-titer plates or micro-fermentors. In agreement with the cell arrangement in disrupted biofilm structures (**Figure 7**), the intact biofilm is made of: (i) large DNA-containing forms (∼3–7 μm of width and ∼8–12 μm of length), resembling micro-aggregates made of a few cells tighly packed side by side along the width (**Figure 8C**), and (ii) rod-shaped forms, sometimes rather long (up to ∼15–20 μm long, **Figure 8D**). The *C. difficile* biofilm grown under our conditions therefore displays an irregular, heterogeneous and sparse architecture made of both micro-aggregates and cells. This could represent an example of phenotypic heterogeneity, a well-documented biofilm property in several other species (van Gestel et al., 2015).

Finally, the intact biofilm of *C. difficile* strain 630Δ*erm* grown in TYt medium in a micro-titer plate is shown to display a very original architecture compared to most other biofilms described by CLMS to date (Dawson et al., 2012; Dapa et al., 2013; Semenyuk et al., 2014; Pantaleon et al., 2015). These biofilms of the same or other strains grown in different media and systems are less high (20–30 μm or less) and display a regular and dense architecture (Dapa et al., 2013; Semenyuk et al., 2014; Pantaleon et al., 2015; Maldarelli et al., 2016). A disordered clumping of cells into micro-colonies has previously been observed twice, in strains grown in different systems, either as an early, mono-layered biofilm (Maldarelli et al., 2016) or as a three-dimension biofilm (Semenyuk et al., 2014). Micro-aggregates therefore seem to make part of a few *C. difficile* biofilms under different *in vitro* conditions. Whether the micro-aggregates observed here, apparently made of cells tightly aligned side by side along the width, are the same as the previously described ones that are made of clumped cells, will remain to be established.

#### Microscopic Architecture of Mutant Biofilms

##### *CD2214–CD2215* inactivation mutant

The biofilm phenotype of *CD2214–CD2215* mutant was assessed at the microscopic level. When grown for 48 h in a micro-titer plate system, *CD2214–CD2215* mutant is able to form a biofilm, confirming that CD2214–CD2215 proteins are dispensable for biofilm formation not only in micro-fermentors (**Supplementary Figure S4**) but also in micro-titer plates (**Figure 8**). Yet, the biofilm formed by the *CD2214–CD2215* mutant is different from that formed by the parental strain: it is denser (**Figures 8E,F**), with a wide majority of short rods (**Figure 8H**) and smaller micro-aggregates (**Figure 8G**). The mutant biofilm has a slightly lower biovolume than the biofilm of the parental strain (by a less than 1.2 factor; **Supplementary Figure S6A**). CD2214–CD2215 proteins therefore play a (slight) positive role on biofilm formation under these conditions, in good agreement with their positive control of most genes expressed in micro-fermentor biofilms (**Figure 6**). The mutant biofilm also displays a decreased mean thickness, an increased maximum coverage and a slightly decreased biovolume/surface ratio (**Supplementary Figures S6B–D**), and the latter is consistent with a decreased micro-aggregation, as previously discussed (Maldarelli et al., 2016).

A complementation test was performed by providing ectopic copies of either *CD2214–CD2215* operon or *CD2214* gene alone to the mutant, and using the parental and mutant strains carrying the empty vector as controls (**Supplementary Figure S7**). Differences in biofilm parameters of all these strains were not statistically significant, which might be related to the presence of the vector and/or to the addition of antibiotics. Nevertheless, one biofilm characteristics seems to be partially complemented by *CD2214* and *CD2215* genes together. The biofilm seems to be slightly denser in the mutant strain bearing the vector (negative control, **Supplementary Figure S7B**) than in the corresponding parental positive control (**Supplementary Figure S7A**), as it is (more clearly) the case in the mutant (**Figure 8F**) compared to the parental strain (**Figure 8B**). The fact that the difference between the two control strains bearing the vector is weak (**Supplementary Figures S7A,B**) probably contributes to the difficulty to evidence any complementation. In contrast to *CD2214* alone (**Supplementary Figure S7D**), *CD2214* and *CD2215* genes together (**Supplementary Figure S7C**) seem to restore the sparse architecture of the positive control biofilm (**Supplementary Figure S7A**). In conclusion, there is a trend toward a higher biofilm density in *CD2214–CD2215* mutant bearing the vector, and this weak phenotype seems to depend on CD2214 and CD2215 regulators together or possibly on CD2215 regulator alone (but not on CD2214 alone; **Supplementary Figure S7**).

##### *pilA_1_* and *CD2831* inactivation mutants

The effect of *pilA_1_* or *CD2831* inactivation on biofilm architecture was also examined, after biofilm growth for 24 h in micro-titer plates. Both *pilA_1_* (**Supplementary Figure S8B**) and *CD2831* mutants (**Supplementary Figure S8D**) are able to form a biofilm that could not be distinguished from that of the parental strain (**Supplementary Figures S8A,C**). The *pilA_1_* mutant of strain 630Δ*erm* has therefore no phenotype under these conditions, whereas the *pilA_1_* mutant of strain R20291 is slightly affected in cell clumping in the mono-layered biofilm grown on glass coverslips (Maldarelli et al., 2016), possibly reflecting differences in strains and growth conditions. In conclusion, our results indicate that in strain 630Δ*erm*, *pilA_1_* and *CD2831* genes are dispensable for the formation of biofilms in micro-titer plates as in micro-fermentors.

##### *dccA* over-expression mutant

Given the relatively weak effect of *CD2214–CD2215* genes on biofilm formation and the dispensability of *pilA_1_* and *CD2831* genes, we tested the role of *dccA*, a gene up-regulated in biofilms/planktonic cultures and able, when over-expressed, to increase a macroscopic biofilm (Soutourina et al., 2013; Purcell et al., 2015). We analyzed the effect of its over-expression on the formation of an intact biofilm under our conditions.

Strain 630Δ*erm* p*dccA*, where an ectopic *dccA* gene is under the control of an anhydro-tetracycline inducible promoter on a plasmid, and its control (630Δ*erm* p) were grown in TYt medium in 96 well micro-titer polystyrene plates, and artificially induced during growth as previously described (Soutourina et al., 2013). The resulting intact biofilms were then observed for the first time by CLSM. Strain 630Δ*erm* p*dccA*, compared to strain 630Δ*erm* p, forms more biofilm, with a biovolume increase of 1.6-fold (**Supplementary Figure S9A**), consistent with the biomass increase of its previously studied macroscopic biofilm (Soutourina et al., 2013; Purcell et al., 2015). DccA over-production is therefore confirmed to increase biofilm yield, even under our conditions.

**FIGURE 9:**
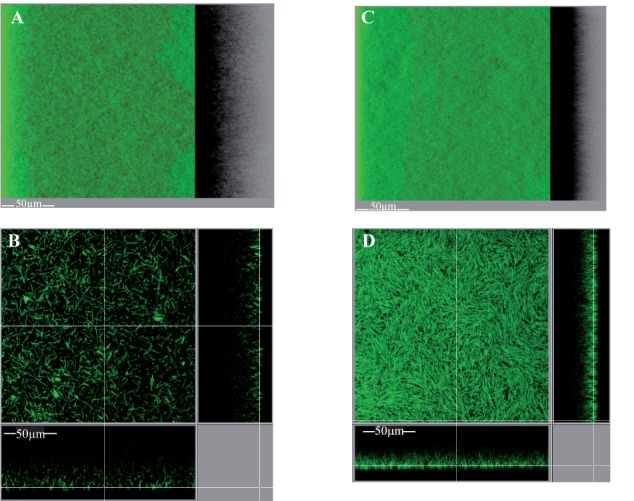
Intact biofilm architecture of the parental strain over-expressing or not *dccA*. Biofilms were grown in TYt medium freshly added onto adhesive starter cells in 96-well polystyrene micro-titer plates. The procedure was essentially as described in **Figure 8**, except that growth was for only 24 h and that anhydro-tetracycline was added to induce *P_tet_* promoter and *dccA* expression. At the end of growth, intact biofilms were stained and observed by CLMS as described in **Figure 8**. Representative images are shown. For each strain, a 3D projection upside view, with its shadow on the right **(A,C)**, and a section view close to the surface **(B,D)** are shown, with the white bar indicating the scale (50 μm). The biofilm of the parental strain over-expressing *dccA* (630Δ*erm* p*dccA* in **C,D**) and that of the control strain (630Δ*erm* p in **A,B**) are shown. After data recovery, the same four parameters as in **Figure 8** were quantified and a statistical analysis was performed (see **Supplementary Figure S9**).

Unexpectedly, however, considering previous, strictly quantitative results (Soutourina et al., 2013; Purcell et al., 2015), the intact biofilm of strain 630Δ*erm* p*dccA* is quite different from that of strain 630Δ*erm* p (**Figure 9**). It displays a new, carpet-like architecture: it is highly homogeneous, dense and largely covering the polystyrene surface in the bottom section (**Figure 9D**). Its maximum coverage is indeed increased by a 2.2-fold factor, and its mean thickness simultaneously decreased by a 1.5-fold factor (**Supplementary Figures S9B,C**). *C. difficile* is thus revealed here for the first time to be able to form biofilms of different architectures depending on DccA levels. *dccA* gene in multi-copy is therefore shown not only to increase biofilm formation, but also to promote a new, homogeneous and dense architecture.

The homogeneous and dense, carpet-like biofilm is composed of tightly packed and aligned DNA-containing forms, arranged as almost contiguous micro-aggregates (**Figure 9D**), and the biovolume/surface ratio of the biofilm is consistently increased (**Supplementary Figure S9D**) (Maldarelli et al., 2016). DccA over-production is thus shown to promote the microscopic packing and aggregation of cells in biofilms, reminiscent of its aggregation effect on planktonic cultures (Purcell et al., 2012; Bordeleau et al., 2015).

All our results, in strains over-expressing or not *dccA*, point to microscopic aggregation playing an important role in biofilm architecture. *C. difficile* is able to form biofilms containing micro-aggregates, which can be arranged either in a highly homogeneous and dense way or in a heterogeneous and sparse way. It will remain to be established whether the few, sparse and small micro-aggregates of the parental strain are formed by the same mechanism as the almost contiguous micro-aggregates of *dccA* over-producing strain. One interesting possibility is that they could both depend on c-di-GMP, but at different concentrations. The homogeneous micro-aggregation could result from a very high c-di-GMP level due to artificial DccA over-production (Purcell et al., 2012; Peltier et al., 2015). The heterogeneous micro-aggregation might be triggered by a lower, intermediate c-di-GMP level, resulting from the concomitant expression of antagonistic DGC- and PDE-encoding genes, as observed in micro-fermentor biofilms. In particular, *dccA* and *CD1421* genes could contribute to heterogeneous micro-aggregation and their control by CD2214–CD2215 (see above) could explain the decreased size and number of micro-aggregates observed in *CD2214–CD2215* mutant biofilm.

##### *pilA_1_* and *CD2831* inactivation mutants over-expressing *dccA*

To get insights on the molecular mechanisms underlying the effet of *dccA* over-expression on biofilm formation under our conditions, we tested whether this effect was mediated by *pilA_1_* or *CD2831*, as these genes are up-regulated when DccA is over-expressed during planktonic growth (Soutourina et al., 2013). The effect of *pilA_1_* or *CD2831* inactivation was therefore reassessed in the context of *dccA* over-expression, using 630Δ*erm* p*dccA* as the control strain.

*pilA_1_* p*dccA* is, like the control strain, able to form a dense and homogeneous, carpet-like biofilm (**Supplementary Figures S10A,B**), but with a slightly affected efficiency. When the biofilms formed by *pilA_1_* p*dccA* and 630Δ*erm* p*dccA* are compared, the biovolume of the former is slightly decreased (by a 0.85-fold factor; not shown). Its maximum coverage is also decreased, indicating that cells are slightly less tightly packed. These results are in agreement with previous ones that have showed that, after growth under different conditions and macroscopic staining, a *pilA_1_* mutant over-expressing *dccA* forms a biofilm of slightly decreased biomass compared to that of the parental control strain (Purcell et al., 2015). All these results show that *pilA_1_* slightly contributes to the DccA-dependent biofilm increase independently of the biofilm growth conditions.

*CD2831* p*dccA* is also able, like the control strain, to form a dense and homogeneous, carpet-like biofilm (**Supplementary Figures S10C,D**), without any significant difference at a neither qualitative nor quantitative level. Furthermore, *CD2831* p*dccA* is able to form such a biofilm, irrespective of whether *dccA* is induced since the beginning of biofilm growth (not shown) or earlier, since pre-culture growth (**Supplementary Figures S10C,D**), in order to early shut off the production of PPEP-1/CD2830 protease and prevent early CD2831 degradation (Peltier et al., 2015). These results show that CD2831 is not required for the DccA-dependent formation of the highly aggregated, homogeneous and dense, carpet-like biofilm.

As neither PilA_1_ nor CD2831 is absolutely required for the DccA-dependent, carpet-like biofilm, the factor mediating this architecture remains unknown. Although it cannot be excluded that both CD2831 and PilA_1_ could be required together in a synergistic manner, the DccA-dependent phenotype could alternatively be mediated by other factors controlled by c-di-GMP. These could be: (i) the response regulator CD3267 and its targets, (ii) the cell-wall anchored protein CD3246, even though its gene does not vary in micro-fermentor biofilm/planktonic cells, or finally (iii) the other proteins controlled by c-di-GMP (Soutourina et al., 2013). Finally, all our results indicate that biofilm formation is more complicated than initially anticipated. They nevertheless reveal that cell micro-aggregation plays an important role in biofilm formation and architecture.

## Conclusion

We showed here that *C. difficile* is able to grow, in TYt medium, as original biofilms compared to most previously described biofilms grown under different conditions. First, at the macroscopic level, in glass micro-fermentors, under a medium flow possibly mimicking the fluid flow in the gut lumen, *C. difficile* form macro-colonies and submersed biofilms loosely adhesive to the surfaces. In these biofilms, gene expression is widely reprogrammed notably with respect to cell surface properties and metabolism, and CD2214 and CD2215 regulators probably contribute to this reprogramming. Carbon metabolism is consistently remodeled in biofilms. Second, at the microscopic level, *C. difficile* cells are embedded into a polymorphic material forming aggregated structures up to 100 μm long. The intact biofilm has an irregular, heterogeneous, sparse and high 3D architecture containing micro-aggregates. *C. difficile* is revealed here to be able to form biofilms of different architectures, more or less micro-aggregated, depending on DccA levels, and thus probably on c-di-GMP concentration in the cells. CD2214-15 participates in the positive control of biofilm yield and architecture, and of cell micro-aggregation. Nevertheless, the mechanisms leading to cell micro-aggregation remain to be established.

## Author Contributions

IP performed most of the experiments, and wrote and edited the manuscript. LS inactivated *CD2214–CD2215* genes and studied their role in the regulation of gene expression, and was deeply involved in the transcriptomics experiments. AC was deeply involved in all observations using CLMS and RB helped in these experiments and their interpretation. MM did the statistical analysis of raw transcriptomic data. JM and J-MG helped in microfermentor experiments. OS helped in *C. difficile* genetics and in preliminary biofilm experiments and provided a strain before to publication. IM-V and BD helped in *C. difficile* genetics and in transcriptomic data interpretation. J-MG, RB, IM-V, and BD helped in editing the manuscript.

## Conflict of Interest Statement

The authors declare that the research was conducted in the absence of any commercial or financial relationships that could be construed as a potential conflict of interest.

## Footnotes

1 https://research.pasteur.fr/en/tool/biofilm-microfermenters/

2 http://www.bioconductor.org

3 http://www.ncbi.nlm.nih.gov/geo/query/acc.cgi?token=crajgeyijrajzqn&acc=GSE85980

4 www6.jouy.inra.fr/mima2

## References

[B1] Abt M. C. Mckenney P. T. Pamer E. G. (2016). *Clostridium difficile* colitis: pathogenesis and host defence. *Nat. Rev. Microbiol.* 14 609–620. 10.1038/nrmicro.2016.108 27573580 PMC5109054

[B2] An D. Parsek M. R. (2007). The promise and peril of transcriptional profiling in biofilm communities. *Curr. Opin. Microbiol.* 10 292–296. 10.1016/j.mib.2007.05.011 17573234

[B3] Andreesen J. R. Wagner M. Sonntag D. Kohlstock M. Harms C. Gursinsky T. (1999). Various functions of selenols and thiols in anaerobic gram-positive, amino acids-utilizing bacteria. *Biofactors* 10 263–270. 10.1002/biof.5520100226 10609892

[B4] Antunes A. Camiade E. Monot M. Courtois E. Barbut F. Sernova N. V. (2012). Global transcriptional control by glucose and carbon regulator CcpA in *Clostridium difficile*. *Nucleic Acids Res.* 40 10701–10718. 10.1093/nar/gks864 22989714 PMC3510511

[B5] Beloin C. Valle J. Latour-Lambert P. Faure P. Kzreminski M. Balestrino D. (2004). Global impact of mature biofilm lifestyle on *Escherichia coli* K-12 gene expression. *Mol. Microbiol.* 51 659–674. 10.1046/j.1365-2958.2003.03865.x 14731270

[B6] Bordeleau E. Fortier L. C. Malouin F. Burrus V. (2011). c-di-GMP turn-over in *Clostridium difficile* is controlled by a plethora of diguanylate cyclases and phosphodiesterases. *PLoS Genet.* 7:e1002039. 10.1371/journal.pgen.1002039 21483756 PMC3069119

[B7] Bordeleau E. Purcell E. B. Lafontaine D. A. Fortier L. C. Tamayo R. Burrus V. (2015). Cyclic di-GMP riboswitch-regulated type IV pili contribute to aggregation of *Clostridium difficile*. *J. Bacteriol.* 197 819–832. 10.1128/JB.02340-14 25512308 PMC4325102

[B8] Bouillaut L. Dubois T. Sonenshein A. L. Dupuy B. (2015). Integration of metabolism and virulence in *Clostridium difficile*. *Res. Microbiol.* 166 375–383. 10.1016/j.resmic.2014.10.002 25445566 PMC4398617

[B9] Bouillaut L. Self W. T. Sonenshein A. L. (2013). Proline-dependent regulation of *Clostridium difficile* stickland metabolism. *J. Bacteriol.* 195 844–854. 10.1128/JB.01492-12 23222730 PMC3562115

[B10] Bridier A. Dubois-Brissonnet F. Boubetra A. Thomas V. Briandet R. (2010). The biofilm architecture of sixty opportunistic pathogens deciphered using a high throughput CLSM method. *J. Microbiol. Methods* 82 64–70. 10.1016/j.mimet.2010.04.006 20433880

[B11] Buckley A. M. Spencer J. Candlish D. Irvine J. J. Douce G. R. (2011). Infection of hamsters with the UK *Clostridium difficile* ribotype 027 outbreak strain R20291. *J. Med. Microbiol.* 60 1174–1180. 10.1099/jmm.0.028514-0 21330415 PMC3167879

[B12] Cafardi V. Biagini M. Martinelli M. Leuzzi R. Rubino J. T. Cantini F. (2013). Identification of a novel zinc metalloprotease through a global analysis of *Clostridium difficile* extracellular proteins. *PLoS One* 8:e81306. 10.1371/journal.pone.0081306 24303041 PMC3841139

[B13] Cairns L. S. Hobley L. Stanley-Wall N. R. (2014). Biofilm formation by *Bacillus subtilis*: new insights into regulatory strategies and assembly mechanisms. *Mol. Microbiol.* 93 587–598. 10.1111/mmi.12697 24988880 PMC4238804

[B14] Carlier J. P. Sellier N. (1989). Gas chromatographic-mass spectral studies after methylation of metabolites produced by some anaerobic bacteria in spent media. *J. Chromatogr.* 493 257–273. 10.1016/S0378-4347(00)82733-4 2584294

[B15] Chu M. Mallozzi M. J. Roxas B. P. Bertolo L. Monteiro M. A. Agellon A. (2016). A *Clostridium difficile* cell wall glycopolymer locus influences bacterial shape. Polysaccharide production and virulence. *PLoS Pathog.* 12:e1005946. 10.1371/journal.ppat.1005946 27741317 PMC5065235

[B16] Collery M. M. Kuehne S. A. Mcbride S. M. Kelly M. L. Monot M. Cockayne A. (2017). What’s a SNP between friends: the influence of single nucleotide polymorphisms on virulence and phenotypes of *Clostridium difficile* strain 630 and derivatives. *Virulence* 8 767–781. 10.1080/21505594.2016.1237333 27652799 PMC5626343

[B17] Crowther G. S. Chilton C. H. Todhunter S. L. Nicholson S. Freeman J. Baines S. D. (2014). Development and validation of a chemostat gut model to study both planktonic and biofilm modes of growth of *Clostridium difficile* and human microbiota. *PLoS One* 9:e88396. 10.1371/journal.pone.0088396 24516647 PMC3916432

[B18] Dapa T. Leuzzi R. Ng Y. K. Baban S. T. Adamo R. Kuehne S. A. (2013). Multiple factors modulate biofilm formation by the anaerobic pathogen *Clostridium difficile*. *J. Bacteriol.* 195 545–555. 10.1128/JB.01980-12 23175653 PMC3554014

[B19] Dapa T. Unnikrishnan M. (2013). Biofilm formation by *Clostridium difficile*. *Gut Microbes* 4 397–402. 10.4161/gmic.25862 23892245 PMC3839985

[B20] Dawson L. F. Valiente E. Faulds-Pain A. Donahue E. H. Wren B. W. (2012). Characterisation of *Clostridium difficile* biofilm formation, a role for Spo0A. *PLoS One* 7:e50527. 10.1371/journal.pone.0050527 23236376 PMC3517584

[B21] Dembek M. Barquist L. Boinett C. J. Cain A. K. Mayho M. Lawley T. D. (2015). High-throughput analysis of gene essentiality and sporulation in *Clostridium difficile*. *mBio* 6:e02383. 10.1128/mBio.02383-14 25714712 PMC4358009

[B22] Dhalluin A. Bourgeois I. Pestel-Caron M. Camiade E. Raux G. Courtin P. (2005). Acd, a peptidoglycan hydrolase of *Clostridium difficile* with N-acetylglucosaminidase activity. *Microbiology* 151 2343–2351. 10.1099/mic.0.27878-0 16000724

[B23] Dineen S. S. Mcbride S. M. Sonenshein A. L. (2010). Integration of metabolism and virulence by *Clostridium difficile* Cody. *J. Bacteriol.* 192 5350–5362. 10.1128/JB.00341-10 20709897 PMC2950512

[B24] Dubois T. Dancer-Thibonnier M. Monot M. Hamiot A. Bouillaut L. Soutourina O. (2016). Control of *Clostridium difficile* physiopathology in response to cysteine availability. *Infect. Immun.* 84 2389–2405. 10.1128/IAI.00121-16 27297391 PMC4962627

[B25] Edwards A. N. Nawrocki K. L. Mcbride S. M. (2014). Conserved oligopeptide permeases modulate sporulation initiation in *Clostridium difficile*. *Infect. Immun.* 82 4276–4291. 10.1128/IAI.02323-14 25069979 PMC4187847

[B26] Elsden S. R. Hilton M. G. (1978). Volatile acid production from threonine, valine, leucine and isoleucine by clostridia. *Arch. Microbiol.* 117 165–172. 10.1007/BF00402304 678022

[B27] Fagan R. P. Fairweather N. F. (2011). *Clostridium difficile* has two parallel and essential Sec secretion systems. *J. Biol. Chem.* 286 27483–27493. 10.1074/jbc.M111.263889 21659510 PMC3149341

[B28] Ferreyra J. A. Wu K. J. Hryckowian A. J. Bouley D. M. Weimer B. C. Sonnenburg J. L. (2014). Gut microbiota-produced succinate promotes *C. difficile* infection after antibiotic treatment or motility disturbance. *Cell Host Microbe* 16 770–777. 10.1016/j.chom.2014.11.003 25498344 PMC4859344

[B29] Frese S. A. Mackenzie D. A. Peterson D. A. Schmaltz R. Fangman T. Zhou Y. (2013). Molecular characterization of host-specific biofilm formation in a vertebrate gut symbiont. *PLoS Genet.* 9:e1004057. 10.1371/journal.pgen.1004057 24385934 PMC3873254

[B30] Ganeshapillai J. Vinogradov E. Rousseau J. Weese J. S. Monteiro M. A. (2008). *Clostridium difficile* cell-surface polysaccharides composed of pentaglycosyl and hexaglycosyl phosphate repeating units. *Carbohydr. Res.* 343 703–710. 10.1016/j.carres.2008.01.002 18237724

[B31] Ghigo J. M. (2001). Natural conjugative plasmids induce bacterial biofilm development. *Nature* 412 442–445. 10.1038/35086581 11473319

[B32] Girinathan B. P. Ou J. Dupuy B. Govind R. (2018). Pleiotropic roles of *Clostridium difficile* sin locus. *PLoS Pathog.* 14:e1006940. 10.1371/journal.ppat.1006940 29529083 PMC5864091

[B33] Gross M. Cramton S. E. Gotz F. Peschel A. (2001). Key role of teichoic acid net charge in *Staphylococcus aureus* colonization of artificial surfaces. *Infect. Immun.* 69 3423–3426. 10.1128/IAI.69.5.3423-3426.2001 11292767 PMC98303

[B34] Hall-Stoodley L. Stoodley P. (2009). Evolving concepts in biofilm infections. *Cell. Microbiol.* 11 1034–1043. 10.1111/j.1462-5822.2009.01323.x 19374653

[B35] Hensbergen P. J. Klychnikov O. I. Bakker D. Dragan I. Kelly M. L. Minton N. P. (2015). *Clostridium difficile* secreted Pro-Pro endopeptidase PPEP-1 (ZMP1/CD2830) modulates adhesion through cleavage of the collagen binding protein CD2831. *FEBS Lett.* 589 3952–3958. 10.1016/j.febslet.2015.10.027 26522134

[B36] Hensbergen P. J. Klychnikov O. I. Bakker D. Van Winden V. J. Ras N. Kemp A. C. (2014). A novel secreted metalloprotease (CD2830) from *Clostridium difficile* cleaves specific proline sequences in LPXTG cell surface proteins. *Mol. Cell. Proteomics* 13 1231–1244. 10.1074/mcp.M113.034728 24623589 PMC4014281

[B37] Hobley L. Harkins C. Macphee C. E. Stanley-Wall N. R. (2015). Giving structure to the biofilm matrix: an overview of individual strategies and emerging common themes. *FEMS Microbiol. Rev.* 39 649–669. 10.1093/femsre/fuv015 25907113 PMC4551309

[B38] James G. A. Chesnel L. Boegli L. Delancey Pulcini E. Fisher S. Stewart P. S. (2017). Analysis of *Clostridium difficile* biofilms: imaging and antimicrobial treatment. *J. Antimicrob. Chemother.* 73 102–108. 10.1093/jac/dkx353 29029221

[B39] Janoir C. (2016). Virulence factors of *Clostridium difficile* and their role during infection. *Anaerobe* 37 13–24. 10.1016/j.anaerobe.2015.10.009 26596863

[B40] Janoir C. Deneve C. Bouttier S. Barbut F. Hoys S. Caleechum L. (2013). Adaptive strategies and pathogenesis of *Clostridium difficile* from in vivo transcriptomics. *Infect. Immun.* 81 3757–3769. 10.1128/IAI.00515-13 23897605 PMC3811758

[B41] Karlsson S. Burman L. G. Akerlund T. (2008). Induction of toxins in *Clostridium difficile* is associated with dramatic changes of its metabolism. *Microbiology* 154 3430–3436. 10.1099/mic.0.2008/019778-0 18957596

[B42] Karlsson S. Lindberg A. Norin E. Burman L. G. Akerlund T. (2000). Toxins, butyric acid, and other short-chain fatty acids are coordinately expressed and down-regulated by cysteine in *Clostridium difficile*. *Infect. Immun.* 68 5881–5888. 10.1128/IAI.68.10.5881-5888.2000 10992498 PMC101550

[B43] Köpke M. Straub M. Durre P. (2013). *Clostridium difficile* is an autotrophic bacterial pathogen. *PLoS One* 8:e62157. 10.1371/journal.pone.0062157 23626782 PMC3633928

[B44] Kovacs-Simon A. Leuzzi R. Kasendra M. Minton N. Titball R. W. Michell S. L. (2014). Lipoprotein CD0873 is a novel adhesin of *Clostridium difficile*. *J. Infect. Dis.* 210 274–284. 10.1093/infdis/jiu070 24482399

[B45] Lawley T. D. Clare S. Walker A. W. Stares M. D. Connor T. R. Raisen C. (2012). Targeted restoration of the intestinal microbiota with a simple, defined bacteriotherapy resolves relapsing *Clostridium difficile* disease in mice. *PLoS Pathog.* 8:e1002995. 10.1371/journal.ppat.1002995 23133377 PMC3486913

[B46] Lazazzera B. A. (2005). Lessons from DNA microarray analysis: the gene expression profile of biofilms. *Curr. Opin. Microbiol.* 8 222–227. 10.1016/j.mib.2005.02.015 15802256

[B47] Leffler D. A. Lamont J. T. (2015). *Clostridium difficile* Infection. *N. Engl. J. Med.* 373 287–288. 10.1056/NEJMra1403772 26176396

[B48] Livak K. J. Schmittgen T. D. (2001). Analysis of relative gene expression data using real-time quantitative PCR and the 2^-ΔΔC_T_^ method. *Methods* 25 402–408. 10.1006/meth.2001.1262 11846609

[B49] Loo C. Y. Mitrakul K. Voss I. B. Hughes C. V. Ganeshkumar N. (2003). Involvement of an inducible fructose phosphotransferase operon in *Streptococcus gordonii* biofilm formation. *J. Bacteriol.* 185 6241–6254. 10.1128/JB.185.21.6241-6254.2003 14563858 PMC219402

[B50] Maldarelli G. A. Piepenbrink K. H. Scott A. J. Freiberg J. A. Song Y. Achermann Y. (2016). Type IV pili promote early biofilm formation by *Clostridium difficile*. *Pathog. Dis.* 74:ftw061. 10.1093/femspd/ftw061 27369898 PMC5985507

[B51] Martin-Verstraete I. Peltier J. Dupuy B. (2016). The regulatory networks that control *Clostridium difficile* toxin synthesis. *Toxins* 8:E153. 10.3390/toxins8050153 27187475 PMC4885068

[B52] Mathur H. Rea M. C. Cotter P. D. Hill C. Ross R. P. (2016). The efficacy of thuricin CD, tigecycline, vancomycin, teicoplanin, rifampicin and nitazoxanide, independently and in paired combinations against *Clostridium difficile* biofilms and planktonic cells. *Gut Pathog.* 8:20. 10.1186/s13099-016-0102-8 27257437 PMC4890490

[B53] McBride S. M. Sonenshein A. L. (2011). The dlt operon confers resistance to cationic antimicrobial peptides in *Clostridium difficile*. *Microbiology* 157 1457–1465. 10.1099/mic.0.045997-0 21330441 PMC3140582

[B54] Melville S. Craig L. (2013). Type IV pili in Gram-positive bacteria. *Microbiol. Mol. Biol. Rev.* 77 323–341. 10.1128/MMBR.00063-12 24006467 PMC3811610

[B55] Murata T. Kawano M. Igarashi K. Yamato I. Kakinuma Y. (2001). Catalytic properties of Na(+)-translocating V-ATPase in *Enterococcus hirae*. *Biochim. Biophys. Acta* 1505 75–81. 10.1016/S0005-2728(00)00278-4 11248190

[B56] Nawrocki K. L. Edwards A. N. Daou N. Bouillaut L. Mcbride S. M. (2016). CodY-dependent regulation of sporulation in *Clostridium difficile*. *J. Bacteriol.* 198 2113–2130. 10.1128/JB.00220-16 27246573 PMC4944225

[B57] Ng K. M. Ferreyra J. A. Higginbottom S. K. Lynch J. B. Kashyap P. C. Gopinath S. (2013). Microbiota-liberated host sugars facilitate post-antibiotic expansion of enteric pathogens. *Nature* 502 96–99. 10.1038/nature12503 23995682 PMC3825626

[B58] Palmer S. R. Crowley P. J. Oli M. W. Ruelf M. A. Michalek S. M. Brady L. J. (2012). YidC1 and YidC2 are functionally distinct proteins involved in protein secretion, biofilm formation and cariogenicity of *Streptococcus mutans*. *Microbiology* 158 1702–1712. 10.1099/mic.0.059139-0 22504439 PMC3542144

[B59] Pantaleon V. Bouttier S. Soavelomandroso A. P. Janoir C. Candela T. (2014). Biofilms of *Clostridium* species. *Anaerobe* 30 193–198. 10.1016/j.anaerobe.2014.09.010 25242197

[B60] Pantaleon V. Soavelomandroso A. P. Bouttier S. Briandet R. Roxas B. Chu M. (2015). The *Clostridium difficile* protease Cwp84 modulates both biofilm formation and cell-surface properties. *PLoS One* 10:e0124971. 10.1371/journal.pone.0124971 25922949 PMC4414356

[B61] Pedrido M. E. De Ona P. Ramirez W. Lenini C. Goni A. Grau R. (2013). Spo0A links de novo fatty acid synthesis to sporulation and biofilm development in *Bacillus subtilis*. *Mol. Microbiol.* 87 348–367. 10.1111/mmi.12102 23170957

[B62] Peltier J. Shaw H. A. Couchman E. C. Dawson L. F. Yu L. Choudhary J. S. (2015). Cyclic diGMP regulates production of sortase substrates of *Clostridium difficile* and their surface exposure through ZmpI protease-mediated cleavage. *J. Biol. Chem.* 290 24453–24469. 10.1074/jbc.M115.665091 26283789 PMC4591827

[B63] Pettit L. J. Browne H. P. Yu L. Smits W. K. Fagan R. P. Barquist L. (2014). Functional genomics reveals that *Clostridium difficile* Spo0A coordinates sporulation, virulence and metabolism. *BMC Genomics* 15:160. 10.1186/1471-2164-15-160 24568651 PMC4028888

[B64] Purcell E. B. Mckee R. W. Bordeleau E. Burrus V. Tamayo R. (2015). Regulation of Type IV Pili contributes to surface behaviors of historical and epidemic strains of *Clostridium difficile*. *J. Bacteriol.* 198 565–577. 10.1128/JB.00816-15 26598364 PMC4719444

[B65] Purcell E. B. Mckee R. W. Courson D. S. Garrett E. M. Mcbride S. M. Cheney R. E. (2017). A nutrient-regulated cyclic diguanylate phosphodiesterase controls *Clostridium difficile* biofilm and toxin production during stationary phase. *Infect. Immun.* 85:e00347-e17. 10.1128/IAI.00347-17 28652311 PMC5563577

[B66] Purcell E. B. Mckee R. W. Mcbride S. M. Waters C. M. Tamayo R. (2012). Cyclic diguanylate inversely regulates motility and aggregation in *Clostridium difficile*. *J. Bacteriol.* 194 3307–3316. 10.1128/JB.00100-12 22522894 PMC3434733

[B67] Reid C. W. Vinogradov E. Li J. Jarrell H. C. Logan S. M. Brisson J. R. (2012). Structural characterization of surface glycans from *Clostridium difficile*. *Carbohydr. Res.* 354 65–73. 10.1016/j.carres.2012.02.002 22560631

[B68] Ren D. Bedzyk L. A. Setlow P. Thomas S. M. Ye R. W. Wood T. K. (2004). Gene expression in *Bacillus subtilis* surface biofilms with and without sporulation and the importance of yveR for biofilm maintenance. *Biotechnol. Bioeng.* 86 344–364. 10.1002/bit.20053 15083514

[B69] Renier S. Chagnot C. Deschamps J. Caccia N. Szlavik J. Joyce S. A. (2014). Inactivation of the SecA2 protein export pathway in *Listeria monocytogenes* promotes cell aggregation, impacts biofilm architecture and induces biofilm formation in environmental condition. *Environ. Microbiol.* 16 1176–1192. 10.1111/1462-2920.12257 24102749

[B70] Resch A. Rosenstein R. Nerz C. Gotz F. (2005). Differential gene expression profiling of *Staphylococcus aureus* cultivated under biofilm and planktonic conditions. *Appl. Environ. Microbiol.* 71 2663–2676. 10.1128/AEM.71.5.2663-2676.2005 15870358 PMC1087559

[B71] Richardson A. R. Somerville G. A. Sonenshein A. L. (2015). Regulating the intersection of metabolism and pathogenesis in Gram-positive bacteria. *Microbiol. Spectr.* 3:MBP-0004-2014. 10.1128/microbiolspec.MBP-0004-2014 26185086 PMC4540601

[B72] Saujet L. Monot M. Dupuy B. Soutourina O. Martin-Verstraete I. (2011). The key sigma factor of transition phase, SigH, controls sporulation, metabolism, and virulence factor expression in *Clostridium difficile*. *J. Bacteriol.* 193 3186–3196. 10.1128/JB.00272-11 21572003 PMC3133256

[B73] Saujet L. Pereira F. C. Serrano M. Soutourina O. Monot M. Shelyakin P. V. (2013). Genome-wide analysis of cell type-specific gene transcription during spore formation in *Clostridium difficile*. *PLoS Genet.* 9:e1003756. 10.1371/journal.pgen.1003756 24098137 PMC3789822

[B74] Schuchmann K. Müller V. (2014). Autotrophy at the thermodynamic limit of life: a model for energy conservation in acetogenic bacteria. *Nat. Rev. Microbiol.* 12 809–821. 10.1038/nrmicro3365 25383604

[B75] Semenyuk E. G. Laning M. L. Foley J. Johnston P. F. Knight K. L. Gerding D. N. (2014). Spore formation and toxin production in *Clostridium difficile* biofilms. *PLoS One* 9:e87757. 10.1371/journal.pone.0087757 24498186 PMC3907560

[B76] Semenyuk E. G. Poroyko V. A. Johnston P. F. Jones S. E. Knight K. L. Gerding D. N. (2015). Analysis of bacterial communities during *Clostridium difficile* infection in the mouse. *Infect. Immun.* 83 4383–4391. 10.1128/IAI.00145-15 26324536 PMC4598419

[B77] Shimazu K. Takahashi Y. Uchikawa Y. Shimazu Y. Yajima A. Takashima E. (2008). Identification of the *Streptococcus gordonii* glmM gene encoding phosphoglucosamine mutase and its role in bacterial cell morphology, biofilm formation, and sensitivity to antibiotics. *FEMS Immunol. Med. Microbiol.* 53 166–177. 10.1111/j.1574-695X.2008.00410.x 18462386

[B78] Smits W. K. Lyras D. Lacy D. B. Wilcox M. H. Kuijper E. J. (2016). *Clostridium difficile* infection. *Nat. Rev. Dis. Primers* 2:16020. 10.1038/nrdp.2016.20 27158839 PMC5453186

[B79] Soavelomandroso A. P. Gaudin F. Hoys S. Nicolas V. Vedantam G. Janoir C. (2017). Biofilm structures in a mono-associated mouse model of *Clostridium difficile* Infection. *Front. Microbiol.* 8:2086. 10.3389/fmicb.2017.02086 29118745 PMC5661025

[B80] Soutourina O. A. Monot M. Boudry P. Saujet L. Pichon C. Sismeiro O. (2013). Genome-wide identification of regulatory RNAs in the human pathogen *Clostridium difficile*. *PLoS Genet.* 9:e1003493. 10.1371/journal.pgen.1003493 23675309 PMC3649979

[B81] Theriot C. M. Young V. B. (2015). Interactions between the gastrointestinal microbiome and *Clostridium difficile*. *Annu. Rev. Microbiol.* 69 445–461. 10.1146/annurev-micro-091014-104115 26488281 PMC4892173

[B82] Valiente E. Bouche L. Hitchen P. Faulds-Pain A. Songane M. Dawson L. F. (2016). Role of glycosyltransferases modifying type B flagellin of emerging hypervirulent *Clostridium difficile* lineages and their impact on motility and biofilm formation. *J. Biol. Chem.* 291 25450–25461. 10.1074/jbc.M116.749523 27703012 PMC5207246

[B83] Valle J. Toledo-Arana A. Berasain C. Ghigo J. M. Amorena B. Penades J. R. (2003). SarA and not sigmaB is essential for biofilm development by *Staphylococcus aureus*. *Mol. Microbiol.* 48 1075–1087. 10.1046/j.1365-2958.2003.03493.x 12753197

[B84] van Gestel J. Vlamakis H. Kolter R. (2015). Division of labor in biofilms: the ecology of cell differentiation. *Microbiol. Spectr.* 3:MB-0002-2014. 10.1128/microbiolspec.MB-0002-2014 26104716

[B85] Walter J. Loach D. M. Alqumber M. Rockel C. Hermann C. Pfitzenmaier M. (2007). D-alanyl ester depletion of teichoic acids in *Lactobacillus reuteri* 100-23 results in impaired colonization of the mouse gastrointestinal tract. *Environ. Microbiol.* 9 1750–1760. 10.1111/j.1462-2920.2007.01292.x 17564608

[B86] Willing S. E. Candela T. Shaw H. A. Seager Z. Mesnage S. Fagan R. P. (2015). *Clostridium difficile* surface proteins are anchored to the cell wall using CWB2 motifs that recognise the anionic polymer PSII. *Mol. Microbiol.* 96 596–608. 10.1111/mmi.12958 25649385 PMC4973711

[B87] Zhang Y. M. Rock C. O. (2008). Membrane lipid homeostasis in bacteria. *Nat. Rev. Microbiol.* 6 222–233. 10.1038/nrmicro1839 18264115

