# Supplementary material for: Clostridium difficile Biofilm: Remodeling Metabolism and Cell Surface to Build a Sparse and Heterogeneously Aggregated Architecture
Source: Front Microbiol. 2018 Sep 12;9:2084. doi: 10.3389/fmicb.2018.02084 (PMC6143707; doi:10.3389/fmicb.2018.02084)
Supplement: Supplementary file 1 [file Table_1.pdf]

Table S1. Strains, plasmids and primers used in this study

A. Strains

**Name**	**Genotype / Construction**	**Reference / origin**
***E. coli* strains**		
NEB 10β	*Δ(ara-leu) 7697 araD139 fhuA ΔlacX74 galK16 galE15 e14- ϕ80*d*lacZΔM15 recA1 relA1 endA1 nupG rpsL* (Str^R) *rph spoT1 Δ(mrr-hsdRMS-mcrBC)*	New England Biolabs
HB101 (RP4)	*supE44 aa14 galK2 lacY1* Δ(*gpt-proA*) 62 *rpsL20* (Str^R) *xyl-5 mtl-1 recA13* Δ(*mcrC-mrr*) $hsdS_B$ (r_B-m_B-) RP4 (Tra^+ IncP Ap^R Km^R Tc^R)	Laboratory stock
***C. difficile* strains**		
630Δ*erm*	Model, toxinogenic strain (parental strain)	Laboratory stock (Hussain et al., 2005)
ClosTron inactivation mutants		
CD2214-CD2215 (or CDIP9)	630Δ*erm CD2214::*Intron(*erm*), Erm^R Tm^S obtained from 630Δerm (pMTL007-'*CD2214'*-142s), Tm^R	This work
CD2831	630Δ*erm CD2831::*Intron(*erm*), Erm^R Tm^S obtained from 630Δerm (pMTL007C-E5-'*CD2831'*-540), Tm^R	This work
$pilA_1$	630Δ*erm* $pilA_1$::Intron(*erm*), Erm^R Tm^S obtained from 630Δerm (pMTL007C-E5-'*CD3513'*-282s), Tm^R	This work
Plasmid containing strains		
630Δ*erm* p (or CDIP219)	630Δ*erm* (pRPF185 P_{tet} Δ*gusA*), Tm^R	(Soutourina et al., 2013)
CD2831 p	*CD2831* (pRPF185 P_{tet} Δ*gusA*), Erm^R Tm^R	This work
$pilA_1$ p	$pilA_1$ (pRPF185 P_{tet} Δ*gusA*), Erm^R Tm^R	This work
dccA over-expression strains		
630Δ*erm* p*dccA* (or CDIP96)	630Δ*erm* (pRPF185 P_{tet} *dccA*), Tm^R	(Soutourina et al., 2013)
CD2831 p*dccA*	*CD2831* (pRPF185 P_{tet} *dccA*), Erm^R Tm^R	This work
$pilA_1$ p*dccA*	$pilA_1$ (pRPF185 P_{tet} *dccA*), Erm^R Tm^R	This work

B. Plasmids

**Name**	**Genotype/Construction**	**Reference / Origin**
Clostron mutagenesis		
pGEM^{T} Easy	TA cloning vector	Promega
pGEM^{T} ::*2214*	pGEM^{T} ::'*CD2214'*-142s	This work
pGEM^{T} ::*2831*	pGEM^{T} ::'*CD2831'*-540s	This work
pGEM^{T} ::*3513*	pGEM^{T} ::'*CD3513'*-282s	This work
pMTL007	*E. coli/C. difficile* shuttle plasmid, *catP*, Cm^{R}/Tm^{R} ClosTron mutagenesis, Group II Intron, *ermB*-RAM	(Heap et al., 2007)
pMTL-*2214* (or pDIA5918)	pMTL007::'*CD2214'*-142s	This work
pMTL007C-E5	*E. coli*/*C. difficile* shuttle plasmid, *catP*, Cm^{R}/Tm^{R} ClosTron mutagenesis, Group II Intron, *ermB*-RAM	(Heap et al., 2010)
pMTL-C-*2831*	pMTL007C-E5::'*CD2831*'-540s	This work
pMTL-C-*3513*	pMTL007C-E5::'*CD3513*'-282s	This work
Expression vectors and plasmids		
pMTL84121	*E. coli*/*C. difficile* shuttle plasmid, *catP*, Cm^{R}/Tm^{R}	(Heap et al., 2009)
pDIA5945	pMTL84121::*CD2214*	This work
pDIA5946	pMTL84121::*CD2214-CD2215*	This work
p (or pDIA6103)	*E. coli*/*C. difficile* shuttle plasmid, *catP*, Cm^{R}/Tm^{R} cloning and over-expression vector pRPF185 P_{tet} Δ*gusA*	(Soutourina et al., 2013)
p*dccA* (or pDIA5987)	pRPF185 P_{tet} *dccA* inducible expression of *dccA-CD1420* under P_{tet} control	(Soutourina et al., 2013)

C. Primers

Name	Sequence
Clostron mutagenesis	
EBSu (universal)	CGAAATTAGAAACTTGCGTTCAGTAAAC
CD2214	
IMV373 142s-IBS	AAAAAAGCTTATAATTATCCTTAGAGACCAATACAGTGCGCCCAGATAGGGTG
IMV374 142s-EBS1d	CAGATTGTACAAATGTGGTGATAACAGATAAGTCAATACATCTAACTTACCTTTCTTTGT
IMV375 142s-EBS2	TGAACGCAAGTTTCTAATTTCGATTGTCTCTCGATAGAGGAAAGTGTCT
CD2831	
CD2831-540s-IBS	AAAAAAGCTTATAATTATCCTTAAATGACGACTATGTGCGCCCAGATAGGGTG
CD2831-540s-EBS1d	CAGATTGTACAAATGTGGTGATAACAGATAAGTCGACTATGGTAACTTACCTTTCTTTGT
CD2831-540s-EBS2	TGAACGCAAGTTTCTAATTTCGGTTTCATTCCGATAGAGGAAAGTGTCT
CD3513	
CD3513-282s-IBS	AAAAAAGCTTATAATTATCCTTAGATATCGGTGGAGTGCGCCCAGATAGGGTG
CD3513-282s-EBS1d	CAGATTGTACAAATGTGGTGATAACAGATAAGTCGGTGGAAATAACTTACCTTTCTTTGT
CD3513-282s-EBS2	TGAACGCAAGTTTCTAATTTCGATTATATCTCGATAGAGGAAAGTGTCT
Sequencing	
pMTL007 / pMTL007C-E5	
pMTL007-F	TTAAGGAGGTGTATTTCATATGACCATGATTACG
pMTL007-R	AGGGTATCCCCAGTTAGTGTTAAGTCTTGG
pMTL84121	
M13R	CAG GAA ACA GCT ATG AC
M13F	GTTTTCCCAGTCACGAC
pGEM^{T}-easy	
SP6	ATTTAGGTGACACTATAGAATAC
T7	GTAATACGACTCACTATAGGG
Verification of Clostron insertion	
ermB gene (Intron)	
RAM-F (ErmRAM-F)	ACGCGTTATATTGATAAAAATAATAATAGTGGG
RAM-R (ErmRAM-R)	ACGCGTGCGACTCATAGAATTATTTCCTCCCG

**Name**	**Sequence**
Verification of Clostron insertion	
CD2214 gene	
OS238	TTGGTCAGATTGAAAGAGCTGA
OS239	CAGAGTTATCAACGCCTTCTGTT
CD2831 gene	
IP1 (*2831*-5' Intron)	CAA GCT TTA GAA GAT GAA AAA CC
IMV582 (3' Intron)	GCATCTGGAACATCCGTTTT
CD3513 gene	
IP2 (*3513*-5' Intron)	TTA CAC CAG ATG GTC AAA CTG G
IP3 (*3513*-3' Intron)	GTG CTT CTG TTA AGG TAA CTC C
**qRT-PCR**	
rpoA-CD0098	
QRTBD005	TAA AGG TAG AGG TTA TGT TTC TGC T
QRTBD006	TTT GAC CAA CTC TTG TGT TTT CC
flgB-CD0245	
OS513	TGATGCTATGCCTAAAATAGAAGAAA
OS514	CCATTTGCAAAACTTATCAAAGC
CD0581	
IMV593	AAACGGATACCGCTCTTGTG
IMV594	CCAATGGTCGTGACGTGTAG
serA-CD0995	
IMV619	GGCGGTTTAGTTGATGAAAAA
IMV620	GCCCCAATATGAGGTGTAGG
dccA-CD1420	
OS528	TGACCCATTAACTGGAGCAT
OS529	CTTGTCTCCTACATTATGACCTTCA
CD1581	
LS17	CAGCTAAATGGTTCCAAAATGA
LS18	TCCTGAATCTGAACATCCACA
CD1616	
IMV617	TGGCAAAAGAGTTGGGTATG
IMV618	CTTCAATTGGCATTGGCTTT

Name	Sequence
**qRT-PCR**	
CD2214	
LS09	AAGGCAGGTTTACATCCAACA
LS10	TTTGCAAATAACAATTCAAGTGG
CD2215	
OS398	3'TCATTTTCTAAATATGTTTTTCCAGTG
OS399	5'ATGGTTGGAGTATCGCCTTG
pilW-CD2305	
IMV585	TGGCTTTAAAGATGACCCATT
IMV586	TTCATGACCAGGAGATGACATT
cat1-CD2343	
IMV589	AGAAAATTGTGCCCATCCAG
IMV590	CCATGATAAAGCTTCGTCCAA
cwp84-CD2787	
2787-ql	TGGCAAATGGTGTAAATGGA
2787-qr	TCAGCACCTTGCAACTCACT
cwp66-CD2789	
IMV470	TCAAGCTGGGTGGACAAAGT
IMV471	ATATTGGCGCATCTTTTGCT
CD2831	
OS582	AAAAGAAGGGGAACCTGTGC
OS583	CTCCTGGAGGACTTGAACCA
malY-CD3029	
LS45	AAATTGGTCATGCCAGAAGG
LS46	CAGACCCCGACATAATAGCAA
pilA$_1$-CD3513	
OS580	CAGTAGTGGCAGTTCCAGCTT
OS581	CCAGTTTGACCATCTGGTGT
luxS-CD3598	
3598-ql	GGGGAGATGTAGATGCCAAA
3598-qr	TCCTTTGCCCCAAATAAAGA
dnaF-CD1305	
RT_polIII-F_*Cdiff*	TCCATCTATTGCAGGGTGGT
RT_polIII-R_*Cdiff*	CCCAACTCTTCGCTAAGCAC

References

Heap, J.T., Kuehne, S.A., Ehsaan, M., Cartman, S.T., Cooksley, C.M., Scott, J.C., and Minton, N.P. (2010). The ClosTron: Mutagenesis in *Clostridium* refined and streamlined. *J Microbiol Methods* 80**,** 49-55.

Heap, J.T., Pennington, O.J., Cartman, S.T., Carter, G.P., and Minton, N.P. (2007). The ClosTron: a universal gene knock-out system for the genus *Clostridium*. *J Microbiol Methods* 70**,** 452-464.

Heap, J.T., Pennington, O.J., Cartman, S.T., and Minton, N.P. (2009). A modular system for *Clostridium* shuttle plasmids. *J Microbiol Methods* 78**,** 79-85.

Hussain, H.A., Roberts, A.P., and Mullany, P. (2005). Generation of an erythromycin-sensitive derivative of *Clostridium difficile* strain 630 (630Δ*erm*) and demonstration that the conjugative transposon Tn*916*ΔE enters the genome of this strain at multiple sites. *J Med Microbiol* 54**,** 137-141.

Soutourina, O.A., Monot, M., Boudry, P., Saujet, L., Pichon, C., Sismeiro, O., Semenova, E., Severinov, K., Le Bouguenec, C., Coppee, J.Y., Dupuy, B., and Martin-Verstraete, I. (2013). Genome-wide identification of regulatory RNAs in the human pathogen *Clostridium difficile*. *PLoS Genet* 9**,** e1003493.
