# Supplementary material for: Clostridium difficile Biofilm: Remodeling Metabolism and Cell Surface to Build a Sparse and Heterogeneously Aggregated Architecture
Source: Front Microbiol. 2018 Sep 12;9:2084. doi: 10.3389/fmicb.2018.02084 (PMC6143707; doi:10.3389/fmicb.2018.02084)
Supplement: Supplementary file 2 [file Table_2.PDF]

Table S2. Genes differentially expressed in biofilm compared to planktonic cells

Gene ID	Name	Gene product / Function	Biofilm / planktonic growth Micro-array	qRT-PCR	Comment [1] c-di-GMP riboswitch
**Sugar transport**					
**Uptake systems for sugars**					
**PTS systems**					
PTS_Glucose-maltose_CD3027-3031					
CD3027		PTS system, glucose-like IIA component	17.17		
CD3028		Putative phosphosugar isomerase	16.55		
CD3030		PTS system, glucose-like IIBC component	16.34		
CD3031		Transcription antiterminator, PTS operon regulator	15.06		
PTS_Glucose_CD2666-2667					
CD2666	*ptsG-A*	PTS system, glucose-specific IIA component	16.69		
CD2667	*ptsG-BC*	PTS system, glucose-specific IIBC component	13.32		
PTS_Glucose-like_CD3088-3089					
CD3089		PTS system, glucose-like IIBC component	16.13		
CD3088		Putative cellobiose-phosphate degrading protein	9.06		
PTS_Mannose_CD3012-3016					
CD3013		PTS system, mannose-specfic IIC component	15.26		
CD3012		Putative alpha-mannosidase	14.12		
CD3014		PTS system, mannose-specfic IIB component	10.41		
CD3015		PTS system, mannose-specfic IIA component	10.04		
CD3016		Transcription antiterminator, PTS operon regulator	7.03		
PTS_Beta-glucoside_CD3136-3138					
CD3136	*bglA*	6-phospho-beta-glucosidase	7.31		
CD3137	*bglF*	PTS system, beta-glucoside-specific IIABC component	6.90		
PTS_Beta-glucoside_CD3115-3117					
CD3115	*bglA*	6-phospho-beta-glucosidase	4.45		
CD3116	*bglF*	PTS system, beta-glucoside-specific IIABC component	4.04		
Other PTS components					
CD2756	*ptsH*	PTS system, HPr protein	3.61		
CD2331	*mtlD*	Mannitol-1-phosphate 5-dehydrogenase	3.09		
CD2880	*celC*	PTS system, cellobiose-specific IIA component	2.64		
CD2512		PTS system, glucose-like IIA component	2.39		
CD0209		Putative tagatose 6-phosphate kinase	2.28		
CD0819		Transcription antiterminator, PTS operon regulator	2.03		
CD0491		PTS system, mannose/fructose/sorbose IIA component	0.37		
CD1073		Transcriptional regulator, GntR family	0.27		
CD1077		PTS system, mannose/fructose/sorbose IIC component	0.19		
CD0763	*srlM*	Sorbitol operon activator protein (Glucitol)	0.24		
CD2488		PTS system, fructose-like IIA component	0.15		
**ABC systems for sugar uptake**					
ABC_Sugar_CD2547-2550					
CD2548		ABC-type transport system, sugar-family permease	7.66		
CD2547		Conserved hypothetical protein	7.50		
CD2550		ABC-type transport system, sugar-family extracellular solute-b	7.06		
CD2549		ABC-type transport system, sugar-family permease	5.60		
ABC_CD0873-CD0875					
CD0873		ABC-type transport system, sugar-family extracellular solute-b	6.03		
CD0874		ABC-type transport system, sugar-family ATP-binding protein	4.36		
CD2456		ABC-type transport system, sugar-family ATP-binding protein	2.90		
Ribose_uptake_CD0298-0302					
CD0300	*rbsB*	ABC-type transport system, ribose-specific extracellular solute	0.14		
CD0301	*rbsA*	ABC-type transport system, ribose-specific ATP-binding protei	0.13		
CD0302	*rbsC*	ABC-type transport system, ribose-specific permease	0.12		
**Other sugar uptake systems**					
CD3017		Putative glucose uptake protein	5.77		

Gene ID	Name	Gene product / Function	Biofilm / planktonic growth Micro-array	qRT-PCR	Comment [1] c-di-GMP riboswitch
**Central carbon metabolism**					
**Glycolysis**					
CD3285	*pgi*	Glucose-6-phosphate isomerase (GPI) (Phosphoglucose isome	2.68		
CD0403	*fba*	Fructose-1,6-bisphosphate aldolase	3.18		
CD3174	*gapA*	Glyceraldehyde-3-phosphate dehydrogenase (GAPDH)	3.42		
CD3394	*pyk*	Pyruvate kinase (PK)	3.18		
CD0582		Putative pyruvate phosphate dikinase, PEP/pyruvate-binding	2.79		
**Pentose Phosphate**					
CD2329	*tal*	Transaldolase	3.64		
CD2321	*tkt'*	Transketolase, central and C-terminal (Sedoheptulose-7-phos	3.10		
CD2322	*tkt*	Transketolase, N-terminal (Sedoheptulose-7-phosphate:D-glyc	2.67		
CD3480	*rpiB2*	Ribose-5-phosphate isomerase 2	2.85		
**Glycogenesis**					
Glycogen_biosynthesis_CD0882-0886					
CD0882	*glgC*	Glucose-1-phosphate adenylyltransferase	0.18		
CD0883	*glgD*	Glycogen biosynthesis protein	0.18		
CD0885	*glgP*	Glycogen phosphorylase	0.31		
CD0884	*glgA*	Glycogen synthase (Starch [bacterial glycogen] synthase)	0.27		
**Gluconeogenesis**					
CD2412		Putative negative regulator of gluconeogenesis	0.3		
CD2411		Putative positive regulator of gluconeogenesis putative phosp	0.27		
CD2410	*ppdK*	Pyruvate phosphate dikinase (ATP:pyruvate, phosphate phosp	0.21		
CD1767	*gapB*	Glyceraldehyde-3-phosphate dehydrogenase (GAPDH)	0.19		
**Pyruvate conversion into Formate and Acetyl-CoA**					
CD0758	*pflA*	Pyruvate formate-lyase activating enzyme	4.78		
CD0759	*plfB*	Formate acetyltransferase (Pyruvate formate-lyase)	3.62		
CD3282	*pflD*	Pyruvate formate-lyase	0.26		
CD3283	*pflE*	Pyruvate formate-lyase (activating enzyme)	0.24		
**Fermentations (carbon sources)**					
**Lactate fermentation**					
CD2164	*ldh*	L-lactate dehydrogenase (L-LDH)	0.27		
**Butyrate fermentation**					
CD2429		Putative flavodoxin/ferredoxin oxidoreductase alpha subunit	0.33		
CD2429A		Putative 4Fe-4S ferredoxin, iron-sulfur binding domain protei	0.24		
CD2425	*ptb*	Phosphate butyryltransferase (Phosphotransbutyrylase)	0.24		
CD0715	*ptb*	Phosphate butyryltransferase (Phosphotransbutyrylase)	0.22		
CD1057	*crt2*	3-hydroxybutyryl-CoA dehydratase (Crotonase)	0.09		
CD1055	*etfB*	Electron transfer flavoproteins subunit beta	0.08		
CD1056	*etfA*	Electron transfer flavoprotein subunit alpha	0.08		
CD1059	*thlA1*	Acetoacetyl-CoA thiolase 1	0.08		
CD1054	*bcd2*	Butyryl-CoA dehydrogenase	0.06		
CD1058	*hbd*	3-hydroxybutyryl-CoA dehydrogenase	0.06		
**Ethanol and butanol production**					
CD2966	*adhE*	Aldehyde-alcohol dehydrogenase	0.23		
CD3105		Putative iron-containing alcohol dehydrogenase	0.11		

Gene ID	Name	Gene product / Function	Biofilm / planktonic growth Micro-array	qRT-PCR	Comment [1] c-di-GMP riboswitch
**CO_2 / formate utilization**					
**Formate dehydrogenase**					
CD2179		Putative anaerobic formate dehydrogenase	2.62		
**Wood Ljungdahl pathway**					
Formate_dehydrogenase_CD3313-3317					
CD3313	*hydN1*	Putative oxidoreductase, Fe-S subunit	0.41		
CD3315A		Conserved hypothetical protein	0.33		
CD3314	*hydA*	Iron hydrogenase	0.32		
CD3316	*fdhD*	Formate dehydrogenase formation protein	0.31		
CD3317	*fdhF*	Formate dehydrogenase-H (selenocysteine)	0.30		
CD3315	*hydN2*	Putative oxidoreductase, Fe-S subunit	0.28		
Wood Ljungdahl pathway, folate, CO-dehydrogenase					
CD0718	*fhs*	Formate--tetrahydrofolate ligase (Formyltetrahydrofolate synt	0.25		
CD0719	*fchA*	Methenyltetrahydrofolate cyclohydrolase (5,10-methenyltetra	0.20		
CD0720	*folD*	Bifunctional protein folD [Includes: Methylenetetrahydrofolate	0.19		
CD0721		Conserved hypothetical protein	0.23		
CD0722	*metF*	5,10-methylenetetrahydrofolate reductase	0.20		
CD0723		Bifunctional carbon monoxide dehydrogenase/acetyl-CoA synt	0.21		
CD0724		Bifunctional carbon monoxide dehydrogenase/acetyl-CoA synt	0.21		
CD0725		Bifunctional carbon monoxide dehydrogenase/acetyl-CoA synt	0.22		
CD0726		Bifunctional carbon monoxide dehydrogenase/acetyl-CoA synt	0.19		
CD0728		Bifunctional carbon monoxide dehydrogenase/acetyl-CoA synt	0.33		
CD0727		Bifunctional carbon monoxide dehydrogenase/acetyl-CoA synt	0.26		
CD0716	*cooS*	Bifunctional carbon monoxide dehydrogenase/acetyl-CoA synt	0.14		
CD0717		Bifunctional carbon monoxide dehydrogenase/acetyl-CoA synt	0.13		
**Succinate utilization**					
CD2338	*4hbD*	4-hydroxybutyrate dehydrogenase (4-hydroxybutanoate:NAD-	4.56		
CD2340		Conserved hypothetical protein	4.19		
CD2344		Permease, membrane protein	3.61		
CD2342	*sucD*	Succinate-semialdehyde dehydrogenase (NAD(P)+)	3.47		
CD2339	*cat2*	4-hydroxybutyrate CoA transferase	3.37		
CD2341	*abfD*	Gamma-aminobutyrate metabolism dehydratase/isomerase [i	3.22		
CD2343	*cat1*	Succinyl-CoA:coenzyme A transferase	2.99	4	
**Ethanolamine utilization**					
CD1913	*eutB*	Ethanolamine ammonia lyase large subunit	4.21		
CD1918	*eutM*	Ethanolamine carboxysome structural protein, BMC family	5.14		
CD1920	*eutD*	Putative phosphotransacetylase	2.33		
CD1921		Putative ethanolamine utilization protein	2.56		
**Gene ID**	**Name**	**Gene product / Function**	**Biofilm / planktonic growth Micro-array**	**qRT-PCR**	**Comment [1] c-di-GMP riboswitch**
**Energy generation and conservation**					
**ATP-synthases / ATPases**					
ATP_synthase_CD3467-CD3476					
CD3468	*atpD*	ATP synthase subunit beta (ATPase subunit beta) (ATP synthas	3.38		
CD3467	*atpC*	ATP hydrolase epsilon chain	3.17		
CD3469	*atpG*	ATP synthase gamma chain (ATP synthase F1 sector gamma su	2.90		
CD3470	*atpA*	ATP synthase subunit alpha (ATPase subunit alpha) (ATP synth	2.79		
ATP_synthase_CD2954-2961					
CD2954	*ntpD*	V-type ATP synthase subunit D	0.07		
CD2955	*ntpB*	V-type ATP synthase beta chain (V-type ATPase subunit B)	0.06		
CD2956	*ntpA*	V-type ATP synthase alpha chain (V-type ATPase subunit A)	0.06		
CD2956A	*ntpF*	V-type ATP synthase subunit F	0.04		
CD2957	*ntpC*	V-type ATP synthase subunit C	0.05		
CD2958	*ntpE*	V-type ATP synthase subunit E (V-type ATPase subunit E)	0.05		
CD2959	*ntpK*	V-type ATP synthase subunit K	0.04		
CD2960	*ntpI*	V-type sodium ATP synthase subunit I	0.07		
CD2961		Conserved hypothetical protein	0.09		
**Electron transport**					
CD1142	*rnfB*	Electron transport complex protein	2.67		
CD1171	*etfB*	Electron transfer flavoprotein subunit alpha	0.33		
CD1172	*etfA*	Electron transfer flavoprotein subunit alpha	0.25		
CD1173		Putative FAD-linked oxidase	0.35		
CD1999	*fldX*	Flavodoxin	0.13		

Gene ID	Name	Gene product / Function	Biofilm / planktonic growth Micro-array	qRT-PCR	Comment [1] c-di-GMP riboswitch
**Nitrogen source uptake**					
**Uptake systems for oligo-peptides and amino-acids**					
**ABC systems for oligopeptide uptake**					
ABC_Oligopeptide_CD2670-2674					
CD2672	*appA*	ABC-type transport system, oligopeptide-family solute-binding	13.78		
CD2674	*appC*	ABC-type transport system, oligopeptide-family permease pro	11.28		
CD2673	*appB*	ABC-type transport system, oligopeptide-family permease pro	10.09		
CD2670	*appF*	ABC-type transport system, ATP-binding protein putative oligo	6.53		
CD2671	*appD*	ABC-type transport system, ATP-binding protein putative oligo	6.38		
ABC_Oligopeptide_CD0853-CD0857					
CD0853	*oppB*	ABC-type transport system, oligopeptide-family permease	0.32		
CD0854	*oppC*	ABC-type transport system, oligopeptide-family permease	0.32		
CD0855	*oppA*	ABC-type transport system, oligopeptide-family extracellular s	0.22		
CD0856	*oppD*	ABC-type transport system, ATP-binding component	0.20		
CD0857	*oppF*	Fragment of ABC-type transport system, oligopeptide-family A	0.20		
**Dtp systems for di- or tri-peptide uptake (MFS family)**					
CD3036		Transporter, Major Facilitator Superfamily (MFS)	3.66		
CD2260		Transporter, Major Facilitator Superfamily (MFS)	2.77		
**Other system for peptide transport**					
CD2373		Putative CstA-like carbon starvation protein	0.08		
**ABC system for amino acid uptake**					
CD2176		ABC-type transport system, cystine/aminoacid-family permeas	9.85		
CD2174		ABC-type transport system, cystine/aminoacid-family extracel	9.38		
CD2177		ABC-type transport system, cystine/aminoacid-family extracel	8.81		
CD2175		ABC-type transport system, cystine/aminoacid-family permeas	5.18		
CD2172		ABC-type transport system, cystine/aminoacid-family ATP-bin	3.69		
**Other systems for amino acid uptake**					
CD1555		Putative amino acid permease	2.61		
CD2702	*brnQ*	Branched-chain amino acid transport system II carrier protein	0.37		
CD1260	*brnQ-2*	Branched chain amino acid transport system carrier protein	0.27		
CD1259	*brnQ-1*	Branched chain amino acid transport system carrier protein	0.13		
**Nitrogen metabolism**					
**Peptide degradation, amino acid production**					
CD2173		Putative peptidase, M20D family	5.34		
CD0092	*map1*	Methionine aminopeptidase Map1 (MAP) (Peptidase M)	4.27		
CD2697		Putative peptidase, M20D family	4.87		
CD2613		Putative peptidase, M24 family	3.41		
CD1228		Putative peptidase, U32 family	2.83		
CD0779		Putative amidohydrolase, M20D peptidase family	0.21		
CD0849		Putative glutamate carboxypeptidase	0.45		
CD1646		Putative peptidase, M19 family	0.39		
CD2822		Putative glutamate carboxypeptidase, M20 family	0.41		
CD3183		Putative peptidase, M20 family	0.21		
CD3521		Putative peptidase T, M20B family	0.36		
**Amino acid synthesis**					
**Aspartate biosynthesis**					
CD2516	*ansB*	L-asparaginase	3.34		
CD1339	*aspB*	Aspartate aminotransferase (AspAT) (Transaminase A)	0.29		
**Arginine biosynthesis**					
CD2032	*argB*	Acetylglutamate kinase	0.26		
CD2033	*argJ*	Arginine biosynthesis bifunctional protein ArgJ [Includes: Gluta	0.25		
CD0303	*argE*	Acetylornithine deacetylase ArgE	0.16		
**Histidine biosynthesis**					
CD1549	*hisC*	Histidinol-phosphate aminotransferase (Imidazole acetol-phos	0.33		
CD1550	*hisB*	Imidazoleglycerol-phosphate dehydratase	0.35		
CD1551	*hisH*	Imidazole glycerol phosphate synthase subunit HisH	0.30		
CD1552	*hisA*	1-(5-phosphoribosyl)-5-[(5-phosphoribosylamino)methylidene	0.34		
CD1553	*hisF*	Imidazole glycerol phosphate synthase subunit HisF	0.41		
CD2198		Putative ferredoxin/flavodoxin oxidoreductase, beta subunit	0.39		
CD2200	*hisC*	Histidinol-phosphate aminotransferase	0.31		
**Leucine biosynthesis**					
CD0991	*leuD*	3-isopropylmalate dehydratase small subunit	0.30		
CD0992	*leuB*	3-isopropylmalate dehydrogenase	0.20		
**Isoleucine and Valine biosynthesis**					
CD2014	*ilvD*	Dihydroxy-acid dehydratase	0.25		
**Alanine biosynthesis**					
CD2828		Putative pyridoxal phosphate-dependent transferase	0.16		
**Glycine cleavage system**					
CD0729	*gcvH*	Glycine cleavage system H protein	0.43		

Gene ID	Name	Gene product / Function	Biofilm / planktonic growth		Comment [1]
			Micro-array	qRT-PCR	c-di-GMP riboswitch
**Sulfur source transport**					
**ABC uptake systems for sulfonates**					
ABC_CD2360-CD2365					
CD2362		Fragment of ABC-type transport system, permease (Part 1)	5.57		
CD2361		ABC-type transport system, nitrate/sulfonate/taurine ATP-bin	5.02		
CD2363		Conserved hypothetical protein	5.01		
CD2360		Conserved hypothetical protein	4.34		
CD2365		ABC-type transport system, nitrate/sulfonate/taurine extracel	4.21		
CD2364		Conserved hypothetical protein	3.18		
ABC_Sulfonates_CD1482-1484					
CD1482	*ssuC*	ABC-type transport system, sulfonates-family permease	0.33		
CD1483	*ssuB*	ABC-type transport system, sulfonates-family ATP-binding pro	0.21		
CD1484	*ssuA*	ABC-type transport system, alkanesulfonates-family extracellu	0.21		
**Cysteine / sulfur metabolism**					
**Cysteine metabolism**					
CD3029	*malY*	Bifunctional protein: cystathionine beta-lyase / repressor	20.32	426	
CD1279	*iscS2*	Cysteine desulfurase	0.26		
**Methyl cycle**					
CD3598	*luxS*	S-ribosylhomocysteine lyase / AutoInducer-2 production prote	1	3	
**Fermentations (amino acids)**					
**Stickland reductions**					
**Proline reduction**					
Proline_utilization_CD3236-3244					
CD3241	*prdB*	Proline reductase (selenocysteine)	6.86		
CD3238		Putative component of proline reductase prdE-like	5.37		
CD3240	*prdD*	Proline reductase PrdD	4.63		
CD3239	*prdE*	Proline reductase PrdE	4.42		
CD3243		Conserved hypothetical protein	4.42		
CD3237	*prdF*	Proline racemase	4.09		
CD3236		Putative membrane protein	4.23		
**Glycine reduction**					
Glycine_utilization_CD2348-2358					
CD2348	*grdD*	Glycine reductase complex component C subunit alpha (Protei	0.09		
CD2349	*grdC*	Glycine reductase complex component C subunit beta (Protein	0.08		
CD2351	*grdB*	Glycine reductase complex component B gamma subunit (sele	0.05		
CD2352	*grdA*	Glycine reductase complex selenoprotein A (selenocysteine)	0.04		
CD2354	*grdE*	Glycine reductase complex component B subunits alpha and b	0.05		
CD2355	*trxA2*	Thioredoxin 2 (Trx2)	0.07		
CD2356	*trxB3*	Thioredoxin reductase 3	0.09		
CD2357	*grdX*	Putative glycine reductase complex component	0.32		
**Virulence - Toxin production**					
PaLoc_CD0659-0664					
CD0663	*tcdA*	Toxin A	0.04		

Gene ID	Name	Gene product / Function	Biofilm / planktonic growth Micro-array	qRT-PCR	Comment [1] c-di-GMP riboswitch
**Envelope biogenesis**					
**Membrane biogenesis**					
**Fatty acid biosynthesis**					
CD1184	*fabF*	3-oxoacyl-[acyl-carrier-protein] synthase 2	4.37		
CD1183	*acpP*	Acyl carrier protein (ACP)	3.31		
CD1179	*fabH*	3-oxoacyl-[acyl-carrier-protein] synthase 3	3.10		
CD1180	*fabK*	Enoyl-(Acyl-carrier-protein) reductase II	2.91		
CD1177	*fapR*	Transcriptional regulator, DeoR family (Fatty acid and phosphc	2.88		
CD1181	*fabD*	Malonyl CoA-acyl carrier protein transacylase (MCT)	2.86		
CD1182	*fabG*	3-oxoacyl-[acyl-carrier protein] reductase (3-ketoacyl-acyl carr	2.85		
CD1062	*acpP*	Acyl carrier protein (ACP)	3.24		
**Phospholipid biosynthesis**					
CD1178	*plsX*	Fatty acid/phospholipid synthesis protein PlsX	3.02		
CD2135	*cdsA*	Phosphatidate cytidylyltransferase CdsA	2.66		
**Cell wall biogenesis**					
**Peptidoglycan biosynthesis**					
CD2239	*nanT*	Putative Na+/solute symporter, SSS family	4.52		
CD2240	*nanA*	Acetylneuraminate lyase	5.56		
CD2241	*nanE*	N-acetylmannosamine-6-phosphate 2-epimerase (ManNAc-6-l	3.57		
CD0120	*glmS*	Glucosamine--fructose-6-phosphate aminotransferase [isomer	4.09		
CD0119	*glmM*	Phosphoglucosamine mutase	4.01		
CD2664	*murE*	UDP-N-acetylmuramyl-tripeptide synthetase	5.38		
CD3518	*murC*	UDP-N-acetylmuramate--L-alanine ligase (UDP-N-acetylmuran	2.69		
CD0123	*murA*	UDP-N-acetylglucosamine 1-carboxyvinyltransferase	2.52		
CD1408	*ddl*	D-alanine--D-alanine ligase (D-alanylalanine synthetase) (D-Ala	3.37		
CD3464		Conserved hypothetical protein	0.44		
CD3463	*alr2*	Alanine racemase 2	0.42		
CD3046	*murQ*	N-acetylmuramic acid 6-phosphate etherase	0.21		
CD2130	*dxr*	1-deoxy-D-xylulose 5-phosphate reductoisomerase	0.24		
**Peptidoglycan hydrolysis**					
CD0527		Putative beta-lactamase-like hydrolase	2.15		
CD2498	*dacF*	D-alanyl-D-alanine carboxypeptidase (putative sporulation-spe	2.08		
CD1469	*cwp20*	Putative cell surface protein putative penicillin-binding protei	0.46		
CD1036	*cwp17*	Putative N-acetylmuramoyl-L-alanine amidase, autolysin	0.34		
CD2141		Serine-type D-Ala-D-Ala carboxypeptidase	0.32		
CD1802		Putative hydrolase, metallo-beta-lactamase superfamily	0.31		
CD0784		Putative N-acetylmuramoyl-L-alanine amidase	0.30		
CD1304	*acd*	Mannosyl-glycoprotein endo-beta-N-acetylglucosamidase	0.30		
CD1291	*dacF*	D-alanyl-D-alanine carboxypeptidase	0.24		
CD1389		Putative chloromuconate cycloisomerase	0.14		
**Cell shape**					
CD1145	*mreB2*	Rod shape-determining protein MreB	2.62		
**Surface glycopolymers**					
**Wall polysaccharide biogenesis**					
**PSII and/or PSIII biogenesis** (Anionic polymer locus)					
CD2766	*lcpB*	Ligase anchoring polysaccharide to the cell wall	4.64		
CD2778		Putative glycosyl transferase	2.36		
CD2764		Putative hydrolase, HAD superfamily, IIB subfamily	2.28		
CD2762	*uppS*	Putative undecaprenyl pyrophosphate synthetase	0.30		
**Other polysaccharide biogenesis**					
Cell-surface_protein_CD1028-1029					
CD1029		Putative membrane protein	0.36		
CD1030		Putative glycosyl transferase, family 2	0.24		
CD1319		Putative polysaccharide deacetylase	0.18		
**D-Alanylation of wall polysaccharides**					
Teichoic-acid_biosynthesis_CD2851-2854					
CD2851	*dltC*	D-alanine--poly(phosphoribitol) ligase subunit 2 (D-alanyl carri	3.42		
CD2852	*dltB*	D-alanyl transferase DltB, MBOAT family	5.21		
CD2853	*dltA*	D-alanine--poly(phosphoribitol) ligase subunit 1	2.97		
CD2854	*dltD*	D-alanine transferase DltD	2.38		

Gene ID	Name	Gene product / Function	Biofilm / planktonic growth Micro-array	qRT-PCR	Comment[1] c-di-GMP riboswitch
**Protein export**					
**General Sec translocon**					
CD0059	*secE*	Preprotein translocase SecE subunit	**3.71**		
CD0090	*prlA*	Preprotein translocase SecY subunit	**3.08**		
CD2801	*yajC*	Conserved hypothetical protein	**3.13**		
**Membrane insertion**					
CD3678	*yidC-oxaA*	Sporulation membrane protein SpoIIIJ	**3.34**		
**Dedicated $SecA_2$ protein**					
CD2792	*secA2*	Dedicated translocase SecA2	**3.53**		
**Extra-cellular folding**					
CD2263	*prsA*	Peptidyl-prolyl cis-trans isomerase, PpiC-type	**3.76**		
**Extra-cellular proteins**					
CD0738		Putative exported protein	**2.70**		
CD2831		Putative adhesin	**2.76**	9	Type II
CD2830	*zmp1*	Extracellular Zinc metalloprotease	**0.21**		Type I
**Cell wall proteins (Cwp) of unknown function**					
CD2796	*cwp10*	Cell surface protein	**3.19**		
CD2782	*cwp7*	Cell wall binding protein	**2.60**		
CD2518	*cwp29*	Cell surface protein	**0.22**		
CD2787	*cwp84*	Cell surface protein, protease	1	3	
CD2789	*cwp66*	Cell surface protein	1	2	
**Organelles**					
**Type IV pilus biogenesis**					
**Primary Machinery**					
CD3504		Putative type IV prepilin peptidase, A24A family	**2.69**		
CD3506		Conserved hypothetical protein	**2.63**		
CD3507		Putative type IV pilin	**3.43**		
CD3508		Putative type IV pilin	**3.69**		
CD3509		Putative type IV pilus assembly protein	**3.34**		
CD3510		Putative membrane protein	**3.29**		
CD3511		Putative type IV pilus secretion protein	**3.21**		
**Type IV pilin**					
CD3513	*$pilA_1$*	Putative pilin protein	**2.61**	52	Type II
CD2305	*pilW*	Putative pilin protein	**3.50**	57	
**Flagellum biogenesis**					
CD0234	*csrA*	Carbon storage regulator homolog CsrA	**0.43**		
CD0254	*flgD*	Basal-body rod modification protein FlgD	**0.31**		
CD0255	*flgE*	Flagellar hook protein FlgE (Distal rod protein)	**0.37**		
CD0255A	*flbD*	Flagellar protein FlbD	**0.36**		
CD0263	*flhA*	Flagellar biosynthesis protein FlhA	**0.25**		
CD0245	*flgB*	Flagellar basal-body rod protein FlgB	1	1	

Gene ID	Name	Gene product / Function	Biofilm / planktonic growth Micro-array	qRT-PCR	Comment[1] c-di-GMP riboswitch
**Regulation**					
**HTH Regulators**					
CD3087		Transcriptional regulator, RpiR family	3.92		
CD2234		Transcriptional regulator, Crp family	3.56		
CD0615		Transcriptional regulator, TetR family	2.72		
CD0312		Transcriptional regulator, ArsR family	3.01		
CD1079		Transcriptional regulator, LysR family	2.39		
CD2527		Transcriptional regulator, TetR family	2.38		
CD3583		Transcriptional regulator, MerR family	2.31		
CD2530		Transcriptional regulator, AraC family	2.20		
CD1345		Transcriptional regulator, PadR family	0.42		
CD3037		Transcriptional regulator, CarD family	0.44		
CD0658	*cdu1*	Transcriptional regulator, beta-lactams repressor	0.40		
CD1169		Transcriptional regulator, GntR family	0.39		
CD2308		Transcriptional regulator, HTH-type	0.36		
CD0532		Putative transcriptional regulator	0.35		
CD1278	*iscR*	Transcriptional regulator, Rrf2 family	0.40		
CD1755A		Transcriptional regulator, HTH-type	0.35		
CD0694		Transcriptional regulator, TetR family	0.31		
CD1994A		Transcriptional regulator, ArsR family	0.30		
CD3166		Transcriptional regulator, HxlR family	0.30		
CD0023	*ctsR*	Transcriptional regulator, CtsR family	0.27		
CD2949		Transcriptional regulator, Phage-type	0.25		
CD1606		Transcriptional regulator, GntR family	0.15		
CD1381		Transcriptional regulator, TetR family	0.18		
CD3260	*phoU*	Phosphate uptake regulator, PhoU	0.14		
CD2214		Transcriptional regulator, HTH-type	1	13	
CD2215		Transcriptional regulator, HTH-type	1	10	
**Two component systems**					
CD3267		Two-component response regulator	3.68		Type II
CD3265		Two-component response regulator	2.73		
CD1530		Two-component sensor histidine kinase	2.73		
CD3255	*rgaR*	Two-component response regulator VirR-like	2.65		
CD2113		Two-component sensor histidine kinase	2.59		
CD2114		Two-component response regulator	2.48		
CD3203		Two-component response regulator	0.38		
CD1959		Two-component response regulator	0.29		
CD1957		Two-component response regulator	0.28		
CD0611		Two-component response regulator	0.27		
CD2535		Two-component sensor histidine kinase	0.27		
CD1739		Two-component response regulator, sigma-54 dependent reg	0.24		
**Sigma54 dependent regulators**					
CD2383		Transcriptional regulator, sigma-54-dependent	2.99		
CD0402		Transcriptional regulator, sigma-54-dependent	0.40		
**Antiterminator**					
CD2668		Transcription antiterminator, licT family	3.67		
**Transcription**					
CD0098	*rpoA*	DNA-directed RNA polymerase subunit alpha	3.59	8	
CD1498	*sigA2*	RNA polymerase sigma factor SigA2 (sigma-43)	0.34		
**Signaling proteins**					
CD2384		Putative diguanylate cyclase	4.06		
CD2385		Putative diguanylate cyclase	3.73		
CD1420	*dccA*	Diguanylate cyclase	2.37	9	
CD1421		Putative phosphodiesterase	2.04		
CD2134		Putative phosphodiesterase	2.97		
CD1840		Putative phosphodiesterase	0.43		
CD1185		Putative diguanylate cyclase	0.37		
CD1538		Putative signaling protein	0.40		

Gene ID	Name	Gene product / Function	Biofilm / planktonic growth Micro-array	qRT-PCR	Comment [1] c-di-GMP riboswitch
**Translation**					
**Ribosomal proteins**					
CD0063	*rplJ*	50S ribosomal protein L10	10.5		
CD0064	*rplL*	50S ribosomal protein L7/L12	8.36		
CD0062	*rplA*	50S ribosomal protein L1	7.35		
CD0061	*rplK*	50S ribosomal protein L11	5.30		
CD0058A	*rpmG*	50S ribosomal protein L33	4.57		
CD0060	*nusG*	transcription antitermination protein	4.22		
CD0104	*rplM*	50S ribosomal protein L13	8.43		
CD0105	*rpsI*	30S ribosomal protein S9	4.58		
CD2473	*rpsT*	30S ribosomal protein S20	7.52		
CD1315A		Conserved hypothetical protein	6.63		
CD1316	*rpsO*	30S ribosomal protein S15	6.02		
CD1257	*rplS*	50S ribosomal protein L19	6.37		
CD3661A	*rpsR*	30S ribosomal protein S18	6.30		
CD3663	*rpsF*	30S ribosomal protein S6	5.55		
CD2562A	*rpmB*	50S ribosomal protein L28	6.26		
CD3486A	*rpmE*	50S ribosomal protein L31	6.09		
CD0686	*rpmI*	50S ribosomal protein L35	5.36		
CD0685	*infC*	Translation initiation factor IF-3	4.29		
CD0687	*rplT*	50S ribosomal protein L20	4.02		
CD3680	*rpmH*	50S ribosomal protein L34	5.31		
CD1162		Putative ribosome-associated protein	5.12		
CD1163	*rpmA*	50S ribosomal protein L27	4.81		
CD1161	*rplU*	50S ribosomal protein L21	4.79		
CD0099	*rplQ*	50S ribosomal protein L17	4.70		
CD1176A	*rpmF*	50S ribosomal protein L32	4.48		
CD0068	*rpsL*	30S ribosomal protein S12	4.17		
CD0070	*fusA*	Elongation factor G (EF-G)	4.08		
CD0069	*rpsG*	30S ribosomal protein S7	3.88		
CD0072	*rpsJ*	30S ribosomal protein S10	3.32		
CD0087	*rplR*	50S ribosomal protein L18	3.10		
CD0089	*rplO*	50S ribosomal protein L15	3.04		
CD0080	*rplP*	50S ribosomal protein L16	2.93		
CD0084A	*rpsZ*	30S ribosomal protein S14 type Z	2.85		
CD1253	*rpsP*	30S ribosomal protein S16	4.12		
CD2140	*rpsB*	30S ribosomal protein S2	3.87		
CD2139	*tsf*	Elongation factor Ts (EF-Ts)	3.25		
CD0096	*rpsK*	30S ribosomal protein S11	3.60		
CD0097	*rpsD*	30S ribosomal protein S4	3.31		
CD0095	*rpsM*	30S ribosomal protein S13	3.04		
CD0094	*infA*	Translation initiation factor IF-1	3.02		
CD0093		Ribosomal protein L14E/L6E/L27E-like	2.70		
CD0088	*rpsE*	30S ribosomal protein S5	2.67		
CD0088A	*rpmD*	50S ribosomal protein L30	2.63		
CD0086	*rplF*	50S ribosomal protein L6	2.58		
CD0073	*rplC*	50S ribosomal protein L3	2.57		
CD0085	*rpsH*	30S ribosomal protein S8	2.50		
CD1165		Putative ribosome-associated protein	2.34		
CD2450	*prmA*	Ribosomal protein L11 methyltransferase (L11 Mtase)	0.46		
CD1486		Putative ribosome recycling factor	0.21		
CD0022	*fusA1*	Elongation factor G (EF-G)	0.16		
**tRNA biosynthesis and processing**					
CD3602		tRNA_processing, putative ATPase	3.57		
CD3256	*valS*	valyl-tRNA synthetase	3.15		
CD2446		Putative aspartyl/glutamyl-tRNA amidotransferase subunit B-r	3.07		
CD3552	*lysS*	Lysyl-tRNA synthetase (Lysine--tRNA ligase) (LysRS)	2.70		
CD2618	*ileS*	Isoleucyl-tRNA synthetase	2.60		
CD2610	*trpS*	Tryptophanyl-tRNA synthetase (Tryptophan--tRNA ligase) (Trp	2.31		
CD3502	*pth*	Peptidyl-tRNA hydrolase (PTH)	2.22		
CD3539		Putative deoxyribonuclease	2.20		
CD0051	*gltX*	Glutamyl-tRNA synthetase	2.15		
CD1764		Putative phenylalanyl-tRNA synthetase beta chain	0.36		
**Cytoplasmic protein folding**					
CD1357		Putative peptidyl-prolyl isomerase	3.19		

Gene ID	Name	Gene product / Function	Biofilm / planktonic growth Micro-array	qRT-PCR	Comment [1] c-di-GMP riboswitch
**Stress**					
CD2149		Putative vancomycin resistance protein, vanW family	4.63		
CD0892	*cspA*	Cold shock protein	3.26		
CD0194	*groL*	60 kDa chaperonin (Protein Cpn60) (GroEL protein)	2.98		
CD1355	*cspB*	Cold shock protein CspB	2.97		
CD2978		Putative CRISPR-associated Cas3 family helicase	0.42		
CD2980		Putative CRISPR-associated autoregulator DevR family protein	0.32		
CD2454		Conserved hypothetical protein	0.40		
CD2453		Putative CRISPR-associated negative autoregulator	0.35		
CD3120		Transcriptional regulator, AbrB family	0.38		
CD1652		Putative tellurium resistance protein	0.29		
CD1823		Conserved hypothetical protein, UPF0246 family	0.24		
CD1822	*bcp*	Putative thiol peroxidase	0.21		
CD0488		Putative small multidrug resistance SugE-like protein	0.18		
CD2845	*rbr*	Rubrerythrin	0.16		
**Cell Factors**					
CD3496	*hbs*	Non-specific DNA-binding protein HBsu signal recognition part	4.16		
CD3018		Putative redox-active protein	4.14		
CD2116	*bipA*	GTP-binding protein BipA	4.00		
CD2662		Putative GTP-binding protein	3.12		
CD2467	*lepA*	GTP-binding protein LepA	2.63		
CD2120		Putative 2-nitropropane dioxygenase	2.40		
CD0528		Putative amidohydrolase	3.14		
CD1258		Putative GTPase, MG442 type	2.81		
CD1227		Putative O-methyltransferase	2.39		
CD0673		Putative methyltransferase	2.27		
CD0346		Putative phosphoesterase	2.16		
CD2377		Putative hydrolase, NUDIX family	0.43		
CD2156	*thiH*	Thiamine biosynthesis protein ThiH	0.42		
CD1765		Putative hydrolase, NUDIX family	0.41		
CD1612		Putative amidohydrolase	0.34		
CD1261		Putative ribonucleotide-diphosphate reductase	0.32		
CD1417		Putative ATP-binding protein	0.30		
CD2868		Putative oxidoreductase	0.30		
CD2413		Putative NAD(P)-binding protein	0.29		
CD2983		Putative Helix-Turn-Helix DNA-binding protein	0.27		
CD1403		Putative synthetase	0.25		
CD0732		Putative radical SAM superfamily protein	0.24		
CD0730		Putative iron-sulfur protein	0.20		
CD0731		Putative radical SAM superfamily protein	0.17		
CD0733		Putative biotin/lipoate-protein ligase	0.17		
**Sporulation**					
CD3678A		Conserved hypothetical protein	3.49		
CD3677		Putative SpoIIIJ-associated RNA/ssDNA-binding protein Jag	3.26		
CD3497		Bifunctional protein [Includes: Tetrapyrrole methylase NTP py	3.01		
CD3498	*spoVB*	Stage V sporulation protein B	2.74		
CD1213	*spoIVB*	Stage IV sporulation protein B, peptidase S55 family	0.35		
CD1192	*spoIIIAA*	Stage III sporulation protein AA	0.34		
CD1197	*spoIIIAF*	Stage III sporulation protein AF	0.18		
CD1195	*spoIIIAD*	Stage III sporulation protein AD	0.16		
CD1193	*spoIIIAB*	Stage III sporulation protein AB	0.12		
CD1194	*spoIIIAC*	Stage III sporulation protein AC	0.11		
CD1198	*spoIIIAG*	Stage III sporulation protein AG	0.08		
CD2442	*spoIV*	Stage IV sporulation protein	0.31		
CD2443		Conserved hypothetical protein	0.13		
CD0775	*spoVAE*	Stage V sporulation protein AE	0.25		
CD0774	*spoVAD*	Stage V sporulation protein AD	0.14		
CD2246	*cspC*	Subtilisin-like serine germination related protease	0.31		
CD2247	*cspBA*	Subtilisin-like serine germination related protease	0.21		
CD3499	*spoVT*	Stage V sporulation protein T	0.14		
CD2629	*spoIVA*	Stage IV sporulation protein A	0.11		
CD3490	*spoIIE*	Phosphoprotein phosphatase	0.11		
CD0126	*spoIIID*	Stage III sporulation protein D	0.10		
CD1511	*cotB*	Spore outer coat layer protein CotB	0.08		

Gene ID	Name	Gene product / Function	Biofilm / planktonic growth Micro-array	qRT-PCR	Comment [1] c-di-GMP riboswitch
**Other transport systems**					
**Uptake systems**					
**Other ABC uptake systems**					
CD2997		ABC-type transport system, iron-family ATP-binding protein	2.80		
CD3215		ABC-type transport system, glycine betaine/carnitine/choline /	0.38		
CD2875	*fhuC*	ABC-type transport system, ferrichrome-specific ATP-binding p	0.34		
CD0869	*modA*	ABC-type transport system, molybdenum-specific extracellular	0.27		
**Ferrous iron uptake systems (Feo)**					
CD1518	*feoA*	Ferrous iron transport protein	0.33		
CD1479	*feoB1*	Ferrous iron transport protein B	0.15		
CD1477	*feoA*	Ferrous iron transport protein	0.12		
CD1480		Conserved hypothetical protein	0.12		
CD1478	*feoA*	Ferrous iron transport protein	0.11		
**Efflux systems**					
**ABC efflux systems of the MDR family**					
CD2367		ABC-type transport system, multidrug-family permease	6.84		
CD1473		ABC-type transport system, multidrug-family ATP-binding/per	0.48		
CD1472		ABC-type transport system, multidrug-family ATP-binding prot	0.45		
CD1050		ABC-type transport system, multidrug-family ATP-binding prot	0.39		
CD1607		ABC-type transport system, multidrug-family ATP-binding prot	0.31		
CD0480	*spaG*	ABC-type transport system, lantibiotic/multidrug-family perme	0.19		
**ABC efflux systems**					
CD1528		ABC-type transport system, ATP-binding protein	2.78		
CD1527		ABC-type transport system, permease	2.64		
CD1529		ABC-type transport system, permease	2.40		
CD1532		ABC-type transport system, ATP-binding protein	0.35		
**P-type ATPases**					
CD0313		Putative K/Mg/Cd/Cu/Zn/Na/Ca/Na/H-transporting P-type AT	3.86		
CD3377	*mgtA*	Magnesium-transporting ATPase, P-type 1 Tn916-like, CTn7-O	2.64		
CD2833		Putative calcium-transporting ATPase	0.17		
CD2115		Putative copper-transporting P-type ATPase	0.09		
**ABC components**					
CD0785		ABC-type transport system, ATP-binding protein	2.45		
CD2068		ABC-type transport system, ATP-binding protein	2.22		
CD3216		ABC-type transport system, glycine betaine/carnitine/choline	0.27		
CD1904		ABC-type transport system, permease	0.15		
**Symport systems**					
CD2171		Fragment of putative sodium:dicarboxylate symporter (Part 1)	7.48		
CD3286		Putative sodium:phosphate symporter	3.48		
CD2541		Sodium:glutamate/aspartate symporter family	2.83		
**Other**					
CD0012		Putative small-molecule-binding protein	4.72		
CD0013		Putative mechanosensitive ion channel protein	3.38		
CD3019		Putative transporter	4.10		
CD0745		Putative OmpA/MotB proton channel	2.82		
CD0744		Putative MotA/TolQ/ExbB proton channel	2.68		
CD2594	*uraA*	Putative uracil permease	2.49		
CD1506		Putative drug/sodium antiporter, MATE family	2.38		
CD3169		Putative malate transporter	0.47		
CD3168		Conserved hypothetical protein	0.32		
CD2107		Xanthine/uracil/thiamine/ascorbate permease family protein	0.44		
CD3299		Transporter, Major Facilitator Superfamily (MFS)	0.44		
CD0147		Putative transporter	0.28		
CD2737		Putative nitrilase/cyanide hydratase and apolipoprotein N-acy	0.28		
CD2738		Putative cytosine permease	0.26		
CD2091		Putative xanthine/uracil permease	0.19		

Gene ID	Name	Gene product / Function	Biofilm / planktonic growth Micro-array	qRT-PCR	Comment [1] c-di-GMP riboswitch
**Nucleic Acid Metabolism**					
CD1683		Putative membrane protein	5.88		
CD1684		Putative radical SAM superfamily protein	5.14		
CD3662	*ssb*	Single-stranded DNA-binding protein (Helix-destabilizing prote	5.74		
CD1254		Putative RNA-binding protein	4.48		
CD1682	*iunH*	Putative nucleoside hydrolase, IUNH family	4.09		
CD2994	*nrdF*	Ribonucleoside-diphosphate reductase subunit beta (Ribonucl	4.50		
CD3496	*hbs*	Non-specific DNA-binding protein HBsu signal recognition part	4.16		
CD0198	*guaA*	Glutamine amidotransferase	3.47		
CD0091	*adk*	Adenylate kinase	3.34		
CD1143		Putative Maf-like protein	3.23		
CD3679	*rnpA*	Ribonuclease P protein component (RNaseP protein) (RNase P	3.17		
CD2704		Putative permease	3.14		
CD3495		Putative RNA-binding S4 domain-containing protein	3.10		
CD3393	*rumA*	23S rRNA (uracil-5-)-methyltransferase RumA (23S rRNA(M-5-	3.08		
CD3479	*upp*	Uracil phosphoribosyltransferase (UMP pyrophosphorylase) (U	3.04		
CD3481		Protein-tyrosine phosphatase reductase	2.88		
CD2705		Putative amidohydrolase	3.01		
CD2691	*hpt*	Hypoxanthine phosphoribosyltransferase	2.87		
CD1144	*radC*	DNA repair protein RadC homolog	2.82		
CD2802	*tgt*	Queuine tRNA-ribosyltransferase (tRNA-guanine transglycosyl	2.70		
CD0015	*tadA*	tRNA specific adenosine deaminase	2.57		
CD0109	*nrdG*	Anaerobic ribonucleoside-triphosphate reductase-activating p	2.57		
CD3514	*prs*	Ribose-phosphate pyrophosphokinase	2.30		
CD0001	*dnaA*	Chromosomal replication initiator protein	2.14		
CD0345		Putative GTP pyrophosphokinase	2.11		
CD0002	*dnaN*	DNA polymerase III subunit beta	2.07		
CD3523	*ksgA*	Dimethyladenosine transferase (S-adenosylmethionine-6-N', N	2.28		
CD2330	*xpt*	Xanthine phosphoribosyltransferase (XPRTase)	2.04		
CD0560	*nfo*	Endonuclease IV	0.49		
CD2083		D-hydantoinase (Dihydropyrimidinase)	0.46		
CD2435	*recO*	DNA repair protein RecO (Recombination protein O)	0.45		
CD2434		Conserved hypothetical protein	0.41		
CD1899		Putative dCMP deaminase	0.40		
CD1893		Putative oligonucleotide binding regulator	0.37		
CDP07		Putative enzyme, helicase family	0.35		
CD0689		Putative nucleotide phosphodiesterase	0.34		
CD2074		Putative FAD-binding subunit of xanthine dehydrogenase	0.34		
CD0840		Putative isomerase/hydrolase	0.30		
CD1328	*recA*	Protein RecA (Recombinase A)	0.29		
CD0489		Putative phosphoribosylaminoimidazole-succinocarboxamide	0.24		
CD0690		Putative nucleotide phosphodiesterase	0.24		
CDP02		Conserved hypothetical protein putative HNH endonuclease	0.23		
CD3235	*ssb*	Single-stranded DNA-binding protein	0.22		
CD2080		Putative FAD-binding subunit of xanthine dehydrogenase	0.20		

Gene ID	Name	Gene product / Function	Biofilm / planktonic growth Micro-array	qRT-PCR	Comment [1] c-di-GMP riboswitch
**Mobile elements**					
CD3334		Putative transcriptional regulator Tn916-like, CTn6-Orf11	3.01		
CD3333A		Putative conjugative transposon protein Tn916-like, CTn6-Orf1	2.47		
CD1232		Putative lipoprotein	0.40		
CDP09		Conserved hypothetical protein	0.40		
CD0932		Putative phage protein	0.38		
CDP06		Hypothetical protein	0.36		
CD0413		Putative single-strand DNA-binding protein Tn1549-like, CTn2-	0.35		
CDP10		Hypothetical protein	0.35		
CD0919		Putative phage protein	0.34		
CD3337		Putative membrane protein Tn916-like, CTn6-Orf14	0.33		
CD1110		Putative hydrolase Tn1549-like, CTn4-Orf9	0.27		
CD1847		Putative conjugative transposon protein Tn1549-like, CTn5-Or	0.26		
CD1847		Putative conjugative transposon protein Tn1549-like, CTn5-Or	0.26		
CD1376		Putative phage protein	0.24		
CD0934		Putative phage protein	0.22		
CD2947		Putative phage protein	0.22		
CDP04		Putative phage capsid protein	0.22		
CD0410		Putative conjugative transposon protein Tn1549-like, CTn2-Or	0.21		
CD3339		Putative conjugative transposon protein Tn916-like, CTn6-Orf1	0.20		
CDP03		Conserved hypothetical protein	0.19		
CD1368		Putative phage cell wall XkdQ-like hydrolase	0.18		
CD0923		Putative phage protein	0.15		
CD3345		Putative conjugative transposon protein DUF961 family Tn539	0.14		
CD1234		Putative phage protein	0.12		
CD1107		Putative cell surface protein Tn1549-like, CTn4-Orf13	0.12		
CD1849		Putative conjugative transfer protein Tn1549-like, CTn2-Orf5	0.12		
CD1117		Putative conjugative transposon protein Tn1549-like, CTn4-Or	0.09		
CD3328		Putative conjugative transposon protein Tn916-like, CTn6-Orf4	0.09		
CD3342A		Putative conjugative transposon protein Tn916-like, CTn1-Orf2	0.09		
CD1116		Putative conjugative transposon protein Tn1549-like, CTn4-Or	0.08		
CD1099A		Conserved hypothetical protein	0.07		
CD1101		Putative mobilization protein Tn1549-like, CTn4-Orf23	0.06		

Gene ID	Name	Gene product / Function	Biofilm / planktonic growth Micro-array	qRT-PCR	Comment [1] c-di-GMP riboswitch
**Cofactor metabolism**					
**CoA metabolim**					
CD1750		Putative CoA enzyme activase	6.83		
CD1749		Putative 2-hydroxyacyl-CoA dehydratase	6.68		
CD1190		Putative acyl-CoA N-acyltransferase	2.01		
CD1796		Putative nitrite and sulfite reductase subunit	0.37		
CD1797		Coenzyme A disulfide reductase (CoA-disulfide reductase) (Co	0.32		
CD1512	*panC*	Pantothenate synthetase	0.35		
CD1513	*panB*	Ketopantoate hydroxymethyltransferase	0.25		
**Ferredoxins, Iron-Sulfur proteins**					
CD0172A	*fdxA*	Ferredoxin (4Fe-4S cluster-containing protein) (fdx-like)	5.82		
CD3026		Conserved hypothetical protein	3.48		
CD3025		Putative ferredoxin, iron-sulphur domain-containing protein	2.40		
CD0474		Putative iron-sulfur protein	2.68		
CD1315	*ribC*	Riboflavin biosynthesis protein	2.61		
CD1414		Putative oxidoreductase, Fe-S subunit	2.21		
CD0850		Putative NifU-like protein	0.40		
CD2382		Putative pyridoxal phosphate-dependent transferase	0.34		
CD2381	*iorA*	Indole pyruvate ferredoxin/flavodoxin oxidoreductase	0.31		
CD1508		Putative iron-sulfur binding protein	0.26		
CD2168	*hcp*	Hydroxylamine reductase	0.15		

Gene ID	Name	Gene product / Function	Biofilm / planktonic growth Micro-array	qRT-PCR	Comment [1] c-di-GMP riboswitch
**Unknown**					
**Putative exported proteins of unknown function**					
**Putative membrane proteins of unknown function**					
CD3073		Putative membrane protein	6.99		
CD1471		Putative membrane protein	4.31		
CD2151		Putative membrane protein, DUF819 family	3.98		
CD2699		Putative membrane protein	3.59		
CD2531		Putative membrane protein	3.33		
CD2520		Putative conjugative transposon antibiotic resistance protein	2.84		
CD1989		Putative membrane protein	2.83		
CD2698		Putative membrane protein	2.63		
CD0529		Putative membrane protein	2.57		
CD0786		Putative membrane protein	2.30		
CD0530		Putative membrane protein	2.25		
CD2821		Putative membrane protein	0.44		
CD3228		Putative membrane protein	0.44		
CD2295		Putative membrane protein	0.36		
CD2129		Putative membrane-associated peptidase, M50 family	0.33		
CD1012		Putative membrane protein	0.29		
CD0830		Putative membrane protein	0.28		
CD1022		Putative membrane protein	0.28		
CD1645		Putative membrane protein	0.27		
CD0777		Putative membrane protein	0.25		
CD2820		Putative membrane protein	0.24		
CD2800		Putative membrane protein	0.20		
CD3458		Putative membrane protein	0.15		
CD3636		Putative membrane protein	0.14		
CD1928		Putative membrane protein	0.12		
CD2625		Putative membrane protein	0.09		
**Putative lipoproteins of unknown function**					
CD1992		Putative lipoprotein	0.42		
CD0173		Putative lipoprotein	0.39		
**Putative proteins of unknown function**					
CD2369		Conserved hypothetical protein	10.93		
CD1990		Putative protein	6.82		
CD2993		Conserved hypothetical protein	6.32		
CD1768		Conserved hypothetical protein	5.78		
CD2368		Conserved hypothetical protein	5.19		
CD2238		Conserved hypothetical protein	5.13		
CD2366		Conserved hypothetical protein	4.75		
CD2386		Conserved hypothetical protein	4.61		
CD1176		Conserved hypothetical protein, DUF177 family	4.56		
CD3072		Conserved hypothetical protein	3.74		
CD3252		Conserved hypothetical protein	3.60		
CD0614		Conserved hypothetical protein	3.27		
CD3290		Conserved hypothetical protein	3.17		
CD3561		Conserved hypothetical protein	3.24		
CD0020		Conserved hypothetical protein	3.13		
CD3665		Conserved hypothetical protein, DUF951 family	2.70		
CD3289		Conserved hypothetical protein	2.67		
CD1542		Conserved hypothetical protein	2.52		
CD3667		Conserved hypothetical protein, SirA family	2.43		
CD2522		Conserved hypothetical protein	2.38		
CD3571		Conserved hypothetical protein	2.22		
CD2136A		Conserved hypothetical protein	2.18		
CD0795		Conserved hypothetical protein, UPF0082 family	1.88		
CD1568		Conserved hypothetical protein	0.53		
CD2627		Conserved hypothetical protein	0.48		
CD2751		Conserved hypothetical protein	0.46		
CD0279A		Conserved hypothetical protein	0.41		
CD0586		Conserved hypothetical protein	0.39		
CD3614		Conserved hypothetical protein, DUF1130 family	0.39		
CD1779		Conserved hypothetical protein	0.38		
CD2157		Conserved hypothetical protein	0.36		
CD1063		Conserved hypothetical protein	0.34		
CD3579		Conserved hypothetical protein	0.34		
CD1992A		Conserved hypothetical protein	0.33		
CD0439		Conserved hypothetical protein	0.32		
CD0590		Conserved hypothetical protein	0.32		
CD2996		Conserved hypothetical protein	0.31		
CD3188		Conserved hypothetical protein	0.31		
CD2046		Conserved hypothetical protein	0.30		

Gene ID	Name	Gene product / Function	Biofilm / planktonic growth		Comment [1]
			Micro-array	qRT-PCR	c-di-GMP riboswitch
**Unknown**					
**Putative proteins of unknown function**					
CDP08		Hypothetical protein	0.30		
CD0632		Conserved hypothetical protein	0.28		
CD3040		Conserved hypothetical protein	0.28		
CD3610		Conserved hypothetical protein	0.28		
CD0172		Conserved hypothetical protein	0.27		
CD0589		Conserved hypothetical protein	0.27		
CD1778		Conserved hypothetical protein	0.26		
CD1880		Conserved hypothetical protein	0.23		
CD2499		Conserved hypothetical protein	0.23		
CD0640		Conserved hypothetical protein	0.22		
CD1622		Conserved hypothetical protein	0.22		
CD0577		Conserved hypothetical protein	0.21		
CD2808		Conserved hypothetical protein	0.20		
CD0778		Conserved hypothetical protein	0.19		
CD0587		Conserved hypothetical protein	0.18		
CD2420		Conserved hypothetical protein	0.18		
CD2409		Conserved hypothetical protein	0.17		
CD0304		Conserved hypothetical protein, DUF1355 family	0.16		
CD0588		Conserved hypothetical protein	0.16		
CD1463		Conserved hypothetical protein	0.16		
CD1543A		Conserved hypothetical protein	0.16		
CD1136		Conserved hypothetical protein	0.15		
CD1575		Conserved hypothetical protein	0.15		
CD0311		Conserved hypothetical protein	0.14		
CD2245A		Conserved hypothetical protein	0.14		
CD3297		Conserved hypothetical protein	0.14		
CD0279		Conserved hypothetical protein	0.13		
CD2962		Conserved hypothetical protein	0.13		
CD3522		Conserved hypothetical protein	0.12		
CD1187		Conserved hypothetical protein	0.10		
CD2752		Conserved hypothetical protein	0.07		

Gene identification number (ID), names and functions correspond to those indicated in the MaGe database Clostriscope (https://www.genoscope.cns.fr).
A gene was considered as differentially expressed when the p-value was < 0.05 (see Material and Methods).
The cut-off for biologically significant change was set at 2-fold and allowed identifying 749 genes. Three additional genes displaying a fold-change > 1.5-fold were also included.

Reference

[1] Soutourina, O.A., Monot, M., Boudry, P., Saujet, L., Pichon, C., Sismeiro, O., Semenova, E., Severinov, K., Le Bouguenec, C., Coppee, J.Y., Dupuy, B., and Martin-Verstraete, I. (2013) Genome-wide identification of regulatory RNAs in the human pathogen *Clostridium difficile. PLoS Genet* 9, e1003493
