# Supplementary material for: Clostridium difficile Biofilm: Remodeling Metabolism and Cell Surface to Build a Sparse and Heterogeneously Aggregated Architecture
Source: Front Microbiol. 2018 Sep 12;9:2084. doi: 10.3389/fmicb.2018.02084 (PMC6143707; doi:10.3389/fmicb.2018.02084)
Supplement: Supplementary file 3 [file Table_3.PDF]

Table S3. Export machinery components and exported proteins whose genes are differentially expressed during biofilm/planktonic growth

Gene		Expresion	Protein name / function	Export signal		Presence in
Short ID	Name	biofilm/plank.		Primary	Secondary	Secretome
**Sec/Yid machinery for protein export-secretion**						
CD0059	*secE*	3.7	Preprotein translocase SecE subunit	TM		
CD0090	*secY*	3.1	Preprotein translocase SecY subunit (PrlA)	TM		
CD2792	$secA_2$	3.5	Dedicated $SecA_2$ subunit (cytoplasmic, associated to SecYEG transmembrane complex)	none		
CD2801	*yajC*	3.1	Preprotein translocase YajC subunit	TM		
CD3678	$oxaA_1$*-yidC*	3.3	Membrane protein insertion in the cytoplasmic membrane	TM		
**Folding factor**						
CD2263	*prsA*	3.8	Exported folding factor, Peptidyl Prolyl Isomerase	TM		
**Extra-cellular/Secreted proteins**						
Sec-independent secretion						
CD0663	*tcdA*	0.04	Toxin A, secreted by a holin-like pathway	NA		
Sec-dependent secretion						
CD0738		2.7	Putative exported protein	SP		
CD2830	*zmp1*	0.2	Extracellular Zinc metalloprotease	SP		+ 1
**Surface or wall proteins**						
Sorting (covalent anchoring)						
CD2831		2.8	Putative adhesin, covalently anchored to the cell wall	SP	LPXTG	+ 1,2
Non covalent anchoring						
PSII binding						
CD2796	*cwp10*	3.2	Cell wall binding protein	SP	CWB2	+ 1
CD2782	*cwp7*	2.6	Cell wall binding protein	SP	CWB2	
CD1469	*cwp20*	0.5	Cell wall binding protein, putative penicillin-binding protein	SP	CWB2	+ 1,2
CD1036	*cwp17*	0.3	Cell wall binding protein, Putative N-acetylmuramoyl-L-alanine amidase, autolysin	SP	CWB2	
CD2518	*cwp29*	0.2	Cell wall binding protein	SP	CWB2	+ 1
Other wall binding						
CD1304	*acd*	0.3	Mannosyl-glycoprotein endo-beta-N-acetylglucosamidase	SP	SH3	+ 1,2
**Lipoproteins**						
CD2672	*appA*	13.8	ABC transport system, oligopeptide-family solute-binding protein	LSP		+ 1,2
CD2174		9.4	ABC transport system, cystine/aminoacid-family solute-binding protein	LSP		
CD2177		8.8	ABC transport system, cystine/aminoacid-family solute-binding protein	LSP		+ 1,2
CD2550		7.1	ABC transport system, sugar-family solute-binding protein	LSP		
CD0873		6.0	ABC transport system sugar-family solute-binding lipoprotein and adhesin	LSP		+ 1,2
CD2365		4.2	ABC transport system, nitrate/sulfonate/taurine solute-binding protein	LSP		+ 2
CD1992		0.4	Putative lipoprotein	LSP		
CD1232		0.4	Putative lipoprotein	LSP		
CD0173		0.4	Putative lipoprotein	LSP		
CD0689		0.3	Putative nucleotide phosphodiesterase	LSP		
CD0869	*modA*	0.3	ABC transport system, molybdenum-specific solute-binding protein	LSP		
CD0855	*oppA*	0.2	ABC transport system, oligopeptide-family solute-binding protein	LSP		+ 1
CD1622		0.2	Putative lipoprotein	LSP		
CD1484	*ssuA*	0.2	ABC transport system, alkanesulfonates-family solute-binding protein	LSP		
CD0300	*rbsB*	0.1	ABC transport system, ribose-specific solute-binding protein	LSP		
**Surface organelles**						
**Type IV pili**						
CD2305	*pilW*	3.5	Type IV pilin, major pilin PilW	SP TIVP		
CD3513	$pilA_1$	2.6	Type IV pilin, major pilin $PilA_1$	SP TIVP		+ 2
CD3506	*pilK*	2.6	Type IV pilus, minor pilin	SP TIVP		
CD3507	*pilU*	3.4	Type IV pilus, minor pilin	SP TIVP		
CD3508	*pilV*	3.7	Type IV pilin, minor pilin	SP TIVP		
CD3509	*pilO*	3.3	Type IV pilus biogenesis, membrane accessory protein PilO	TM		
CD3510	*pilMN*	3.3	Type IV pilus biogenesis, membrane accessory protein, hybrid PilM/PilN protein	TM		
CD3511	*pilC*	3.2	Type IV pilus biogenesis, membrane core protein PilC	TM		
CD3504	*pilD*	2.7	Type IV prepilin peptidase, A24A family	TM		
**Flagella**						
CD0263	*flhA*	0.3	Flagellar biosynthesis protein FlhA	TM		
Flagellar type III secretion						
CD0254	*flgD*	0.3	Basal-body rod modification protein FlgD	T3SS		+ 2
CD0255	*flgE*	0.4	Flagellar hook protein FlgE (Distal rod protein)	T3SS		+ 2
CD0255A	*flbD*	0.4	Flagellar protein FlbD	T3SS		

Gene names and functions correspond to those indicated in the MaGe database Clostriscope (https://www.genoscope.cns.fr). A gene was considered as differentially expressed when the p-value was < 0.05.

Primary export signals

TM Transmembrane domain (for other transmembrane proteins, see Table S2)

SP Signal Peptide

LSP Lipoprotein Signal Peptide

SP TIVP Signal Peptide specific for Type IV pilin

T3SS Type 3 Secretion System, specific for flagellar proteins

NA Not applicable: secretion by a specific holin-like system (TcdE) and release by cell lysis

Secondary export signals

LPXTG LPXTG sorting signal

CWB2 Cell-Wall Binding domain 2

SH3 Putative peptidoglycan binding domain

References

[1] Hensbergen, P.J., Klychnikov, O.I., Bakker, D., van Winden, V.J., Ras, N., Kemp, A.C., Cordfunke, R.A., Dragan, I., Deelder, A.M., Kuijper, E.J., Corver, J., Drijfhout, J.W., and van Leeuwen, H.C. (2014) A novel secreted metalloprotease (CD2830) from *Clostridium difficile* cleaves specific proline sequences in LPXTG cell surface proteins. *Mol Cell Proteomics* 13, 1231-1244.

[2] Cafardi, V., Biagini, M., Martinelli, M., Leuzzi, R., Rubino, J.T., Cantini, F., Norais, N., Scarselli, M., Serruto, D., and Unnikrishnan, M. (2013) Identification of a novel zinc metalloprotease through a global analysis of *Clostridium difficile* extracellular proteins. *PloS One* 8, e81306.
