# Supplementary material for: Clostridium difficile Biofilm: Remodeling Metabolism and Cell Surface to Build a Sparse and Heterogeneously Aggregated Architecture
Source: Front Microbiol. 2018 Sep 12;9:2084. doi: 10.3389/fmicb.2018.02084 (PMC6143707; doi:10.3389/fmicb.2018.02084)
Supplement: Supplementary file 4 [file Table_4.PDF]

Table S4. CD2214-CD2215 regulon compared to the set of genes differentially expressed during biofilm/planktonic growth

Gene-ID	Name	Function	630Δ*erm*/ *CD2214-CD2215* mutant Micro-array	630Δ*erm*/ *CD2214-CD2215* mutant qRT-PCR	Biofilm/ planktonic growth Micro-array	c-di-GMP riboswitch [1]
**Sugar transport**						
**PTS systems for sugar uptake**						
CD3027		PTS system. glucose-like IIA component	**12.50**		**17.17**	
CD3028		Putative phosphosugar isomerase	**25.00**		**16.55**	
CD3030		PTS system. glucose-like IIBC component	**20.00**		**16.34**	
CD3031		Transcription antiterminator. PTS operon regulator	**9.09**		**15.06**	
CD2666	*ptsG-A*	PTS system. glucose-specific IIA component	**3.13**		**16.69**	
CD2667	*ptsG-BC*	PTS system. glucose-specific IIBC component	**2.86**		**13.32**	
CD3013		PTS system. mannose-specfic IIC component	**2.27**		**15.26**	
CD3014		PTS system. mannose-specfic IIB component	**2.17**		**10.41**	
CD3015		PTS system. mannose-specfic IIA component	**2.04**		**10.04**	
CD3115	*bglA*	6-phospho-beta-glucosidase	**3.13**		**4.45**	
CD3116	*bglF*	PTS system. beta-glucoside-specific IIABC component	**2.13**		**4.04**	
CD3136	*bglA*	6-phospho-beta-glucosidase	**3.03**		**7.31**	
CD3137	*bglF*	PTS system. beta-glucoside-specific IIABC component	**2.94**		**6.90**	
CD3138	*bglG*	Transcription antiterminator. PTS operon regulator	**2.22**			
CD2755	*ptsI*	PTS system. EI component	**2.27**			
CD2756	*ptsH*	PTS system. HPr protein	**2.38**		**3.61**	
CD0490		Putative sugar-phosphate dehydrogenase	**1.85**			
CD0763	*srlM*	Sorbitol operon activator protein (Glucitol)	**2.00**			
CD0765	*srlEa*	PTS system. sorbitol-specific IIB N-terminal (Glucitol)	**3.03**			
CD0766	*srlEb*	PTS system. sorbitol-specific IIB C-terminal (Glucitol)	**3.03**			
CD0767	*srlB*	PTS system. sorbitol-specific IIA component (Glucitol)	**3.85**			
CD0768	*srlD*	Sorbitol 6-phosphate 2-dehydrogenase (Glucitol)	**2.86**			
CD0491		PTS system. mannose/fructose/sorbose IIA component	1.64			
CD0492		PTS system. mannose/fructose/sorbose IIB component	1.64			
CD0493		PTS system. mannose/fructose/sorbose IIC component	1.49			
**ABC uptake system**						
CD0873		ABC-type transport system. sugar-family extracellular SBP	**2.63**		**6.03**	
CD0874		ABC-type transport system. sugar-family ATP-binding protein	**2.38**		**4.36**	
**Other transport system**						
CD3169		Putative malate transporter	0.66		**0.47**	

Gene-ID	Name	Function	630Δ*erm*/ *CD2214-CD2215* mutant Micro-array	qRT-PCR	Biofilm/ planktonic growth Micro-array	c-di-GMP riboswitch [1]
**Carbohydrate metabolism**						
**Glycolysis**						
CD3285	*pgi*	Glucose-6-phosphate isomerase (GPI) (Phosphoglucose isomera	1.43		**2.68**	
CD0403	*fba*	Fructose-1.6-bisphosphate aldolase			**3.18**	
CD3174	*gapA*	Glyceraldehyde-3-phosphate dehydrogenase (GAPDH)	**7.14**		**3.42**	
CD3173	*pgk*	Phosphoglycerate kinase	**3.70**			
CD3172	*tpi*	Triosephosphate isomerase	**3.85**			
CD3171	*gpmI*	2.3-bisphosphoglycerate-independent phosphoglycerate mutase	**3.57**			
CD3170	*eno*	Enolase	**2.94**			
CD3394	*pyk*	Pyruvate kinase (PK)	**2.63**		**3.18**	
CD3395	*pfkA*	6-phosphofructokinase	**2.27**			
CD0582		Putative pyruvate phosphate dikinase. PEP/pyruvate-binding	**7.14**		**2.79**	
CD3448		Putative ketose-bisphophate aldolase class II putative tagatose-b	**2.33**			
**Pyruvate conversion into acetyl-CoA**						
CD0758	*pflA*	Pyruvate formate-lyase activating enzyme	**2.04**		**4.78**	
CD0759	*plfB*	Formate acetyltransferase (Pyruvate formate-lyase)	**2.44**		**3.62**	
**Lactate fermentation**						
CD2966	*adhE*	Aldehyde-alcohol dehydrogenase	**5.00**		**0.23**	
**Succinate uptake and utilization**						
CD2338	*4hbD*	4-hydroxybutyrate dehydrogenase	**7.14**		**4.56**	
CD2339	*cat2*	4-hydroxybutyrate CoA transferase	**6.25**		**3.37**	
CD2340		Conserved hypothetical protein	**6.67**		**4.19**	
CD2341	*abfD*	g-aminobutyrate metabolism dehydratase/isomerase	**7.69**		**3.22**	
CD2342	*sucD*	Succinate-semialdehyde dehydrogenase (NAD(P)+)	**10.00**		**3.47**	
CD2343	*cat1*	Succinyl-CoA:coenzyme A transferase	**11.11**	**7.69**	**2.99**	
CD2344		Succinate permease. putative membrane protein	**3.70**		**3.61**	
**Wood Ljungdahl pathway**						
CD0718	*fhs*	Formate--tetrahydrofolate ligase	0.68		**0.25**	
CD0719	*fchA*	Methenyltetrahydrofolate cyclohydrolase (5.10-methenyltetrahydr	0.66		**0.2**	
CD0721		Conserved hypothetical protein	0.61		**0.23**	
CD0723		Bifunctional CO dehydrogenase/acetyl-CoA synthase. dihydrolipo	0.65		**0.21**	
CD0724		Bifunctional CO dehydrogenase/acetyl-CoA synthase.nickel-inse	0.63		**0.21**	
CD0725		Bifunctional CO dehydrogenase/acetyl-CoA synthase. subunit de	0.61		**0.19**	
CD0726		Bifunctional CO dehydrogenase/acetyl-CoA synthase. delta subu	0.61		**0.22**	
CD0727		Bifunctional CO dehydrogenase/acetyl-CoA synthase. methyltran	0.64		**0.26**	
CD0728		Bifunctional CO dehydrogenase/acetyl-CoA synthase. subunit alp	0.67		**0.33**	
CD0716	*cooS*	Bifunctional carbon monoxide dehydrogenase/acetyl-CoA synthase			**0.14**	
CD0717		Bifunctional CO dehydrogenase/acetyl-CoA synthase. accessory	0.58		**0.13**	
**Other**						
CD3091	*treA*	Trehalose-6-phosphate hydrolase	1.89		**4.14**	
CD0582		Putative pyruvate phosphate dikinase. PEP/pyruvate-binding	**7.14**		**2.79**	
**Energy**						
**ATPsynthase/ATPase (V-type)**						
CD2954	*ntpD*	V-type ATP synthase subunit D	0.58		**0.07**	
CD2955	*ntpB*	V-type ATP synthase beta chain (V-type ATPase subunit B)	0.62		**0.06**	
CD2956	*ntpA*	V-type ATP synthase alpha chain (V-type ATPase subunit A)	0.62		**0.06**	
CD2956A	*ntpF*	V-type ATP synthase subunit F	0.56		**0.01**	
CD2957	*ntpC*	V-type ATP synthase subunit C	0.616		**0.05**	
CD2958	*ntpE*	V-type ATP synthase subunit E (V-type ATPase subunit E)	0.666		**0.05**	
CD2959	*ntpK*	V-type ATP synthase subunit K	0.586		**0.04**	
CD2960	*ntpI*	V-type sodium ATP synthase subunit I	0.65		**0.07**	
**Rnf complex**						
CD1137	*rnfC*	Electron transport complex protein	0.58			
CD1138	*rnfD*	Electron transport complex protein	0.56			
CD1139	*rnfG*	Electron transport complex protein	0.53			
CD1140	*rnfE*	Electron transport complex protein	0.56			
CD1141	*rnfA*	Electron transport complex protein	0.55			
CD1142	*rnfB*	Electron transport complex protein	0.59		**2.67**	

Gene-ID	Name	Function	630Δ*erm*/ *CD2214-CD2215* mutant Micro-array	qRT-PCR	Biofilm/ planktonic growth Micro-array	c-di-GMP riboswitch [1]
**Nitrogen source metabolism**						
**Uptake systems for oligo-peptides and amino-acids**						
CD2670	*appF*	ABC-type transport system. ATP-binding protein putative oligope	1.64		**6.53**	
CD2671	*appD*	ABC-type transport system. ATP-binding protein putative oligope	1.92		**6.38**	
CD2672	*appA*	ABC-type transport system. oligopeptide-family solute-binding protein			**13.78**	
CD2673	*appB*	ABC-type transport system. oligopeptide-family permease protein			**10.09**	
CD2674	*appC*	ABC-type transport system. oligopeptide-family permease protein			**11.28**	
**Dtp systems for di- or tri-peptide uptake**						
CD3036		Transporter. Major Facilitator Superfamily (MFS)	**5.26**		**3.66**	
**Other**						
CD2373		Putative CstA-like carbon starvation protein	0.52		**0.08**	
**Peptidases**						
CD2697		Putative peptidase. M20D family	1.85		**4.87**	
CD2698		Putative membrane protein	**2.22**		**2.63**	
CD2699		Putative membrane protein	**2.08**		**3.59**	
CD0779		Putative amidohydrolase. M20D peptidase family	0.56		**0.21**	
CD0777		Putative membrane protein	0.57		**0.25**	
CD0778		Conserved hypothetical protein	0.57		**0.19**	
CD0780		Conserved hypothetical protein. DUF1177 family	0.53			
CD3269		Putative oligoendopeptidase F. M3B family	1.96			
CD0528		Putative amidohydrolase	1.92		**3.14**	
CD0529		Putative membrane protein	**2.08**		**2.57**	
CD3458		Putative membrane protein	0.66		**0.15**	
**Histidine biosynthesis**						
CD1549	*hisC*	Histidinol-phosphate aminotransferase (Imidazole acetol-phosphate transaminase)			**0.33**	
CD1550	*hisB*	Imidazoleglycerol-phosphate dehydratase			**0.35**	
CD1551	*hisH*	Imidazole glycerol phosphate synthase subunit HisH	0.67		**0.3**	
CD1552	*hisA*	1-(5-phosphoribosyl)-5-[(5-phosphoribosylamino)methylideneami	0.63		**0.34**	
CD1553	*hisF*	Imidazole glycerol phosphate synthase subunit HisF			**0.41**	
**Leucine biosynthesis**						
CD0991	*leuD*	3-isopropylmalate dehydratase small subunit			**0.30**	
CD0992	*leuB*	3-isopropylmalate dehydrogenase	1.82		**0.20**	
**Sulfur source transport**						
CD1482	*ssuC*	ABC-type transport system. sulfonates-family permease			**0.33**	
CD1483	*ssuB*	ABC-type transport system. sulfonates-family ATP-binding protein			**0.21**	
CD1484	*ssuA*	ABC-type transport system. alkanesulfonates-family extracellular	0.69		**0.21**	
**Cysteine / sulfur metabolism**						
CD3029	*malY*	Bifunctional protein: cystathionine beta-lyase / repressor	**14.29**	**100,00**	**20.32**	
CD3598	*luxS*	S-ribosylhomocysteine lyase (AI-2 synthesis protein)	1.69			
CD1279	*iscS2*	Cysteine desulfurase			**0.26**	
CD1278	*iscR*	Transcriptional regulator. Rrf2 family	1.75		**0.4**	
**Fermentations (amino acids) - Stickland reactions**						
**Glycine reduction**						
CD2348	*grdD*	Glycine reductase complex component C subunit alpha (Protein PC alpha)			**0.09**	
CD2349	*grdC*	Glycine reductase complex component C subunit beta (Protein P	1.52		**0.08**	
CD2351	*grdB*	Glycine reductase complex component B gamma subunit (selenc	1.69		**0.05**	
CD2352	*grdA*	Glycine reductase complex selenoprotein A (selenocysteine)	1.69		**0.04**	
CD2354	*grdE*	Glycine reductase complex component B subunits alpha and beta (Selenoprotein PB alpha/bet			**0.05**	
CD2355	*trxA2*	Thioredoxin 2 (Trx2)			**0.07**	
CD2356	*trxB3*	Thioredoxin reductase 3			**0.09**	
CD2357	*grdX*	Putative glycine reductase complex component			**0.32**	

Gene-ID	Name	Function	630Δ*erm*/ *CD2214-CD2215* mutant Micro-array	qRT-PCR	Biofilm/ planktonic growth Micro-array	c-di-GMP riboswitch [1]
**Envelope biogenesis**						
**Fatty acid biosynthesis**						
CD1062	*acpP*	Acyl carrier protein (ACP)	1.69		3.24	
**Cell wall**						
CD0119	*glmM*	Phosphoglucosamine mutase	1.52		4.01	
CD0120	*glmS*	Glucosamine-fructose-6-phosphate aminotransferase	1.612		4.09	
CD2239		Putative Na^+/solute symporter, SSS family	2.78		4.52	
CD2240	*nanA*	Acetylneuraminate lyase			5.56	
CD2241	*nanE*	N-acetylmannosamine-6-phosphate 2-epimerase (ManNAc-6-P epimerase)			3.57	
CD1010	*nagA*	N-acetylglucosamine-6-phosphate deacetylase	2.70			
CD1011	*nagB*	Glucosamine-6-phosphate deaminase	2.44			
CD2136	*uppS*	Undecaprenyl pyrophosphate synthetase	0.66		0.3	
**D-Alanylation of wall polysaccharides**						
CD2851	*dltC*	D-alanine--poly(phosphoribitol) ligase subunit 2 (D-alanyl carrier protein) (DCP)			3.42	
CD2852	*dltB*	D-alanyl transferase DltB. MBOAT family	1.45		5.21	
CD2853	*dltA*	D-alanine--poly(phosphoribitol) ligase subunit 1			2.97	
CD2854	*dltD*	D-alanine transferase DltD			2.38	
**Exported proteins**						
CD2831		Putative adhesin	2.17	3,13	2.76	Type II
CD3513		Putative pilin protein	2.63	3,45	2.61	Type II
CD2305		Putative pilin protein	3.33	3,13	3.5	
CD0873		Adhesin and Sugar-binding lipoprotein (ABC transport system)	2.63		6.03	
CD3246		Putative surface protein	2.04			Type II
CD2830	*zmp1*	Extracellular Zinc metalloprotease			0.21	Type I
**Gene-ID**	**Name**	**Function**	**630Δ*erm*/ *CD2214-CD2215* mutant Micro-array**	**qRT-PCR**	**Biofilm/ planktonic growth Micro-array**	**c-di-GMP riboswitch [1]**
**Transcriptional regulator**						
CD0312		Transcriptional regulator. ArsR family	1.49		3.01	
CD1755A		Transcriptional regulator. HTH-type	1.82		0.35	
CD2214		Transcriptional regulator. HTH-type. homologous to B. subtilis Sir	3.702			
CD2215		Transcriptional regulator. HTH-type	4.127			
CD0581		Transcriptional regulator. TetR family	3.13	6,25		
CD2463	*hrcA*	Transcriptional regulator. Heat-inducible repressor HrcA	2.00			
CD2564		Transcriptional regulator. MerR family	2.94			
CD3175	*cggR*	Transcriptional regulator. SorC family	5.88			
CD1998		Transcriptional regulator. TetR family	2.04			
CD2004	*effR*	Transcriptional regulator. MarR family	3.03			
CD1009		Transcriptional regulator. GntR family	3.03			
CD2665		Transcriptional regulator. AraC family	3.23			
CD2345		Transcriptional regulator. LysR family	1.89			
CD0470	*blaR*	Beta-lactamase-inducing penicillin-binding protein	2.27			
CD0471	*blaI*	Transcriptional regulator. Penicillinase repressor	2.38			
CD3158		Transcriptional regulator. TRAP family	3.85			
**Two component system family**						
CD3265		Two-component response regulator	2.27		2.73	
CD3267		Two-component response regulator	2.33		3.68	Type II
**Signaling proteins**						
CD1420	*dccA*	diguanylate cyclase	2.13		2.37	
CD1421		Putative phosphodiesterase	2.44		2.04	
CD2384		Putative diguanylate cyclase	0.68		4.06	
CD1515		Putative phosphodiesterase	0.68			
CD1616		Putative phosphodiesterase	2.70	4,55		
**Other**						
CD1893		Putative oligonucleotide binding regulator	0.62		0.37	
CD1498	*sigA2*	RNA polymerase sigma factor SigA2 (sigma-43)	0.65		0.34	

Gene-ID	Name	Function	630Δ*erm*/ *CD2214-CD2215* mutant Micro-array	qRT-PCR	Biofilm/ planktonic growth Micro-array	c-di-GMP riboswitch [1]
**Other metabolism**						
CD1512	*panC*	Pantothenate synthetase	**0.43**		**0.35**	
CD1513	*panB*	Ketopantoate hydroxymethyltransferase	**0.47**		**0.25**	
CD2737		Putative nitrilase/cyanide hydratase and apolipoprotein N-acyltra	0.59		**0.28**	
CD1508		Putative iron-sulfur binding protein	0.63		**0.26**	
CD1507A		Conserved hypothetical protein	0.59			
CD0730		Putative iron-sulfur protein	0.67		**0.20**	
CD0731		Putative radical SAM superfamily protein	0.58		**0.17**	
CD0732		Putative radical SAM superfamily protein			**0.24**	
CD0733		Putative biotin/lipoate-protein ligase	0.65		**0.17**	
CD0184	*pyrB*	Aspartate carbamoyltransferase)	1.69			
CD0185	*pyrK*	Dihydroorotate dehydrogenase electron transfer subunit	1.96			
CD0186	*pyrD*	Dihydroorotate dehydrogenase catalytic subunit	**2.17**			
CD0594		Conserved hypothetical protein	1.75			
CD0595		Putative nuclease. ParB-like	**2.04**			
CD0994		Putative serine-pyruvate aminotransferase	**2.44**			
CD0995	*serA*	Putative D-3-phosphoglycerate dehydrogenase	**4.17**	**3,57**		
CD0996		Conserved hypothetical protein	**3.13**			
CD2479		Putative molybdenum cofactor biosynthesis	**2.17**			
CD3555	*coaX*	Type III pantothenate kinase	**2.50**			
CD3556		Putative membrane protein	**2.38**			
CD3248		Polysaccharide deacetylase	**3.85**			
**Other transport**						
CD0313		Putative K/Mg/Cd/Cu/Zn/Na/Ca/Na/H-transporting P-type ATPas	1.92		**3.86**	
CD3036		Transporter. Major Facilitator Superfamily (MFS)	**5.26**		**3.66**	
CD2594	*uraA*	ABC-type transport system. uracil-specific permease	**2.33**		**2.49**	
CD2541		Sodium:glutamate/aspartate symporter family	**2.22**		**2.83**	
CD2738		Putative cytosine permease	0.60		**0.26**	
CD1518	*feoA*	Ferrous iron transport protein	0.56		**0.33**	
CD3273	*feoA*	Ferrous iron transport protein	1.72			
CD3274	*feoB*	Ferrous iron transport protein B	1.85			
CD2276		Sodium:alanine symporter	**3.23**			
**Stress**						
CD0193	*groS*	10 kDa chaperonin. GroES protein	1.59			
CD0194	*groL*	60 kDa chaperonin. GroEL protein	1.89		**2.98**	
CD2149		Putative vancomycin resistance protein. vanW family	1.79		**4.63**	

Gene-ID	Name	Function	630Δ*erm*/ *CD2214-CD2215* mutant Micro-array	qRT-PCR	Biofilm/ planktonic growth Micro-array	c-di-GMP riboswitch [1]
**Sporulation**						
CD1492		Two-component sensor histidine kinase. sporulation-associated s	0.56			
CD1613	*cotA*	Spore outer coat layer protein CotA	0.51			
CD2400	*cotJB2*	Spore coat peptide assembly protein CotJB 2	0.63			
CD3349	*bclA3*	Exosporium glycoprotein BclA3	**0.40**			
CD0596		Conserved hypothetical protein	**0.46**			
CD0597	*cotJB1*	Spore coat peptide assembly protein	**0.45**			
CD0598	*cotCB*	Spore-coat protein CotCB manganese catalase	**0.42**			
CD1067		Conserved hypothetical protein	0.52			
CD1063B		Conserved hypothetical protein	0.54			
CD1063C		Conserved hypothetical protein	0.58			
CD1581		Conserved hypothetical protein	**0.33**			
CD1845		Putative membrane protein Tn1549-like. CTn5-Orf1	0.63			
**Other**						
CD3149		DNA helicase	0.67			
**Unknown**						
CD1768		Conserved hypothetical protein	**2.38**		**5.78**	
CD2366		Conserved hypothetical protein	0.65		**4.75**	
CD3252		Conserved hypothetical protein	1.52		**3.6**	
CD0172		Conserved hypothetical protein	0.69		**0.27**	
CD0279		Conserved hypothetical protein	0.61		**0.13**	
CD1568		Conserved hypothetical protein	0.68		**0.53**	
CD1622		Conserved hypothetical protein	0.57		**0.22**	
CD2962		Conserved hypothetical protein	0.63		**0.13**	
CD3148		Hypothetical protein	0.64			
CD3271		Conserved hypothetical protein	1.75			
CD3622		Conserved hypothetical protein. PhnA family	**2.17**			
CDP10		Hypothetical protein	**2.78**			

Gene identification number (ID), names and functions correspond to those indicated in the MaGe database Clostriscope (https://www.genoscope.cns
A gene was considered as differentially expressed when the p-value was < 0.05 (see Material and Methods).

Reference

[1] Soutourina, O.A., Monot, M., Boudry, P., Saujet, L., Pichon, C., Sismeiro, O., Semenova, E., Severinov, K., Le Bouguenec, C., Coppee, J.Y., Dupuy, B., and Martin-Verstraete, I. (2013) Genome-wide identification of regulatory RNAs in the human pathogen *Clostridium difficile. PLoS Genet* 9, e1003493
