# Supplementary material for: Clostridium difficile Biofilm: Remodeling Metabolism and Cell Surface to Build a Sparse and Heterogeneously Aggregated Architecture
Source: Front Microbiol. 2018 Sep 12;9:2084. doi: 10.3389/fmicb.2018.02084 (PMC6143707; doi:10.3389/fmicb.2018.02084)
Supplement: Supplementary file 5 [file Table_5.PDF]

Table S5. CD2214-CD2215 regulon compared among strains

Gene-ID or Name Strain 630	Biofilm/ planktonic growth Micro-array	630Δ*erm*/ *CD2214-CD2215* Micro-array	JIR8094/ *sinRR'* Micro-array*	R20291/ *sinRR'* Micro-array*	Function
**Sugar transport**					
**PTS systems**					
CD3030-malX	UP	UP		DOWN one *malX* gen[e]<br>UP other *malX* gene	PTS system. glucose-like IIBC component
ptsG-A	UP	UP	DOWN	DOWN	PTS system. glucose-specific IIA component
ptsG-BC	UP	UP	DOWN		PTS system. glucose-specific IIBC component
CD3089	UP		UP		PTS system, glucose-like IIBC component
bglA	UP	UP	DOWN both	DOWN *bglA* $_2$	6-phospho-beta-glucosidase
bglA	UP	UP	*bglA* genes		6-phospho-beta-glucosidase
bglF	UP	UP	DOWN both		PTS system. beta-glucoside-specific IIABC component
bglF	UP	UP	*bglF* genes		PTS system. beta-glucoside-specific IIABC component
**Sugar metabolism**					
**Glycolysis**					
gapA	UP	UP	DOWN		Glyceraldehyde-3-phosphate dehydrogenase (GAPDH
pgk		UP	DOWN		Phosphoglycerate kinase
tpi		UP	DOWN		Triosephosphate isomerase
pyk-pykF	UP	UP	DOWN	DOWN	Pyruvate kinase (PK)
**Pentose Phosphate**					
tkt	UP		DOWN		Transketolase
**Glycogenesis**					
glgC	DOWN		DOWN		Glucose-1-phosphate adenylyltransferase
glgD	DOWN		DOWN		Glycogen biosynthesis protein
glgP	DOWN				Glycogen phosphorylase
glgA	DOWN		DOWN		Glycogen synthase (Starch [bacterial glycogen] syntha
**Pyruvate conversion**					
pflA	UP	UP		DOWN	Pyruvate formate-lyase activating enzyme
plfB	UP	UP		DOWN	Formate acetyltransferase (Pyruvate formate-lyase)
pflD	DOWN			UP	Pyruvate formate-lyase activating enzyme
pflE	DOWN			UP	Formate acetyltransferase (Pyruvate formate-lyase)
**Lactate fermentation**					
ldh	DOWN			UP	L-lactate dehydrogenase (L-LDH)
**Ethanol/butanol production**					
adhE	DOWN	UP	DOWN	DOWN	Aldehyde-alcohol dehydrogenase
**Butyrate fermentation**					
thlA1	DOWN		UP	UP	Acetoacetyl-CoA thiolase 1
bcd2	DOWN		UP		Butyryl-CoA dehydrogenase
hbd	DOWN		UP	UP	3-hydroxybutyryl-CoA dehydrogenase
**Succinate utilization**					
abfH-4hbD	UP	UP		UP	4-hydroxybutyrate dehydrogenase
abfT-cat2	UP	UP		UP	4-hydroxybutyrate CoA transferase
CD2340	UP	UP			Conserved hypothetical protein
abfD	UP	UP	UP	UP	γ-aminobutyrate metabolism dehydratase/isomerase
sucD	UP	UP	UP	UP	Succinate-semialdehyde dehydrogenase (NAD(P)+)
cat1	UP	UP	UP	UP	Succinyl-CoA:coenzyme A transferase
CD2344	UP	UP			Succinate permease. putative membrane protein
**Ethanolamine utilization**					
eutB	UP		DOWN		Ethanolamine ammonia lyase large subunit
eutM	UP		DOWN		Ethanolamine carboxysome structural protein, BMC fan
	UP				Putative phosphotransacetylase
eutCTAEKLGQHSVPW			DOWN		
**Wood Ljungdahl pathway**					
CD0718-fhs	DOWN	DOWN			Formate--tetrahydrofolate ligase
CD0719-fchA	DOWN	DOWN			Methenyltetrahydrofolate cyclohydrolase (5.10-metheny
CD0721	DOWN	DOWN			Conserved hypothetical protein
CD0723	DOWN	DOWN			Bifunctional CO dehydrogenase/acetyl-CoA synthase.
CD0724	DOWN	DOWN			Bifunctional CO dehydrogenase/acetyl-CoA synthase.n
CD0725	DOWN	DOWN			Bifunctional CO dehydrogenase/acetyl-CoA synthase.
CD0726	DOWN	DOWN			Bifunctional CO dehydrogenase/acetyl-CoA synthase.
CD0727	DOWN	DOWN			Bifunctional CO dehydrogenase/acetyl-CoA synthase.
CD0728	DOWN	DOWN			Bifunctional CO dehydrogenase/acetyl-CoA synthase.
cooS	DOWN		DOWN		Bifunctional carbon monoxide dehydrogenase/acetyl-C
CD0717	DOWN	DOWN			Bifunctional CO dehydrogenase/acetyl-CoA synthase.

Gene-ID or Name Strain 630	Biofilm/ planktonic growth Micro-array	630Δ*erm*/ *CD2214-CD2215* Micro-array	JIR8094/ *sinRR'* Micro-array	R20291/ *sinRR'* Micro-array	Function
**Energy**					
**ATPsynthase/ATPase (F-type)**					
atpD	UP		DOWN	DOWN	ATP synthase subunit beta (ATPase subunit beta) (AT
atpC	UP		DOWN	DOWN	ATP hydrolase epsilon chain
atpG	UP		DOWN	DOWN	ATP synthase gamma chain (ATP synthase F1 sector (
atpA	UP		DOWN	DOWN	ATP synthase subunit alpha (ATPase subunit alpha) (A
atpF			DOWN	DOWN	Other ATP synthase subunits
atpHBZEI				DOWN	
**ATPsynthase/ATPase (V-type)**					
ntpD	DOWN	DOWN	UP	DOWN	V-type ATP synthase subunit D
ntpB	DOWN	DOWN	UP		V-type ATP synthase beta chain (V-type ATPase subu
ntpA	DOWN	DOWN	UP		V-type ATP synthase alpha chain (V-type ATPase sub
ntpF	DOWN	DOWN	UP		V-type ATP synthase subunit F
ntpC	DOWN	DOWN	UP		V-type ATP synthase subunit C
ntpE	DOWN	DOWN	UP		V-type ATP synthase subunit E (V-type ATPase subun
ntpK	DOWN	DOWN	UP		V-type ATP synthase subunit K
ntpI	DOWN	DOWN	UP		V-type sodium ATP synthase subunit I
**Nitrogen source metabolism**					
**Uptake systems for oligo-peptides and amino-acids**					
appF	UP	UP		DOWN	ABC-type transport system. ATP-binding protein putati
appD	UP	UP			ABC-type transport system. ATP-binding protein putati
appA	UP			DOWN	ABC-type transport system. oligopeptide-family solute-
appB	UP				ABC-type transport system. oligopeptide-family permea
appC	UP				ABC-type transport system. oligopeptide-family permea
oppB	DOWN				ABC-type transport system, oligopeptide-family permea
oppC	DOWN				ABC-type transport system, oligopeptide-family permea
oppA	DOWN			UP	ABC-type transport system, oligopeptide-family extrace
oppD	DOWN		UP		ABC-type transport system, ATP-binding component
oppF	DOWN		UP	UP	Fragment of ABC-type transport system, oligopeptide-f
**Histidine biosynthesis**					
hisG			UP	UP	ATP phosphoribosyltransferase (ATP-PRTase) (ATP-P
CD1549-hisC	DOWN		UP ?	UP ?	Histidinol-phosphate aminotransferase (Imidazole acet
hisB	DOWN				Imidazoleglycerol-phosphate dehydratase
hisH	DOWN				Imidazole glycerol phosphate synthase subunit HisH
hisA	DOWN				1-(5-phosphoribosyl)-5-[(5-phosphoribosylamino)methy
hisF	DOWN				Imidazole glycerol phosphate synthase subunit HisF
CD2198	DOWN				Putative ferredoxin/flavodoxin oxidoreductase, beta su
CD2200-hisC	DOWN		UP ?	UP ?	Histidinol-phosphate aminotransferase
hisZK			UP		
**Sulfur source transport**					
ssuC	DOWN				ABC-type transport system, sulfonates-family permeas
ssuB	DOWN			UP	ABC-type transport system, sulfonates-family ATP-bind
ssuA	DOWN			UP	ABC-type transport system, alkanesulfonates-family ex
**Fermentations (amino acids) - Stickland reactions**					
**Proline reduction**					
prdF	DOWN		DOWN		Proline racemase
CD3238	DOWN				Putative component of proline reductase prdE-like
prdE	DOWN		DOWN		Proline reductase PrdE
prdD	DOWN		DOWN		Proline reductase PrdD
prdB	DOWN		DOWN		Proline reductase (selenocysteine)
CD3243	DOWN				Conserved hypothetical protein
prdA			DOWN		
prdC			DOWN		
**Glycine reduction**					
grdD	DOWN		DOWN	DOWN	Glycine reductase complex component C subunit alpha
grdC	DOWN	UP		DOWN	Glycine reductase complex component C subunit beta
grdB	DOWN	UP	DOWN	DOWN	Glycine reductase complex component B gamma subu
grdA	DOWN	UP	DOWN	DOWN	Glycine reductase complex selenoprotein A (selenocys
grdE	DOWN		DOWN	DOWN	Glycine reductase complex component B subunits alph
trxA2	DOWN				Thioredoxin 2 (Trx2)
trxB3	DOWN		DOWN		Thioredoxin reductase 3
grdX	DOWN				Putative glycine reductase complex component

Gene-ID or Name 630	Biofilm/ planktonic growth Micro-array	630Δ*erm*/ *CD2214-CD2215* Micro-array	JIR8094/ *sinRR'* Micro-array	R20291/ *sinRR'* Micro-array	Function
**Envelope biogenesis**					
**Fatty acid biosynthesis**					
CD1062-acpP	UP	UP			Acyl carrier protein (ACP)
**Cell wall**					
glmM	UP	UP			Phosphoglucosamine mutase
glmS	UP	UP			Glucosamine-fructose-6-phosphate aminotransferase
CD2239	UP	UP			Putative Na^+/solute symporter, SSS family
nanA	UP				Acetylneuraminate lyase
nanE	UP				N-acetylmannosamine-6-phosphate 2-epimerase (Man
nagA		UP			N-acetylglucosamine-6-phosphate deacetylase
nagB		UP			Glucosamine-6-phosphate deaminase
uppS	DOWN	DOWN		UP	Undecaprenyl pyrophosphate synthetase
**D-Alanylation of wall polysaccharides**					
dltC	UP				D-alanine--poly(phosphoribitol) ligase subunit 2 (D-alan
dltB	UP	UP			D-alanyl transferase DltB. MBOAT family
dltA	UP			DOWN	D-alanine--poly(phosphoribitol) ligase subunit 1
dltD	UP				D-alanine transferase DltD
**Protein export**					
**General Sec translocon**					
secE	UP			DOWN	Preprotein translocase SecE subunit
secG	NOT REG		UP		Preprotein translocase SecG subunit
oxaA1-yidC	UP			DOWN	OxaA1-YidC-Sporulation membrane protein SpoIIIJ
**Exported proteins**					
CD2831	UP	UP	DOWN		Putative adhesin
CD2830-zmp1	DOWN		DOWN	DOWN	Extracellular Zinc metalloprotease
CD0873	UP	UP			Adhesin and Sugar-binding lipoprotein (ABC transport s
CD3246	NOT REG	UP	DOWN	NA	Putative surface protein
**Organelles**					
**Type IV pilus biogenesis**					
CD3512	NOT REG		DOWN		Putative Type IV pilus transporter system
Type IV pilin					
pilA$_1$-CD3513	UP	UP		UP *CDR20291_3350*	Putative pilin protein $PilA_1$
pilW-CD2305	UP	UP	DOWN	DOWN *CDR20291_2191*	Putative pilin protein PilW
**Flagellum biogenesis**					
csrA	DOWN			UP	Carbon storage regulator homolog CsrA
flgD	DOWN		DOWN		Basal-body rod modification protein FlgD
flgE	DOWN		DOWN		Flagellar hook protein FlgE (Distal rod protein)
flbD	DOWN				Flagellar protein FlbD
flhA	DOWN				Flagellar biosynthesis protein FlhA
fliCDFGHK			DOWN	UP	
flgBCKL				UP	
numerous *fli*				UP	
numerous *flg*				UP	
**Regulation**					
**Two component system family**					
CD3265	UP	UP			Two-component response regulator
CD3267	UP	UP			Two-component response regulator
**Signaling proteins**					
dccA	UP	UP		DOWN	diguanylate cyclase
CD1421	UP	UP			Putative phosphodiesterase
CD2384	UP	DOWN			Putative diguanylate cyclase
CD1515		DOWN			Putative phosphodiesterase
CD1616		UP			Putative phosphodiesterase

Gene-ID or Name 630	Biofilm/ planktonic growth Micro-array	630Δ*erm*/ *CD2214-CD2215* Micro-array	JIR8094/ *sinRR'* Micro-array	R20291/ *sinRR'* Micro-array	Function
**Sporulation**					
CD1492		DOWN	**UP**	**UP**	Two-component sensor histidine kinase. sporulation-as
cotA		DOWN			Spore outer coat layer protein CotA
cotJB2		DOWN			Spore coat peptide assembly protein CotJB 2
bclA3		DOWN		**UP**	Exosporium glycoprotein BclA3
CD0596		DOWN			Conserved hypothetical protein
cotJB1		DOWN			Spore coat peptide assembly protein
cotCB		DOWN			Spore-coat protein CotCB manganese catalase
CD1067-cdeC		DOWN		**UP**	Conserved hypothetical protein
CD1063B		DOWN		**UP**	Conserved hypothetical protein
CD1063C		DOWN		**UP**	Conserved hypothetical protein
CD1581		DOWN			Conserved hypothetical protein
CD1845		DOWN		**UP**	Putative membrane protein Tn1549-like. CTn5-Orf1

Gene identification number (ID), names and functions correspond to those indicated in the MaGe database Clostriscope (https://www.genoscope.cns.fr
A gene was considered as differentially expressed when the p-value was < 0.05 (see Material and Methods).

*** Reference**

Girinathan, B.P., Ou, J., Dupuy, B., and Govind, R. (2018). Pleiotropic roles of *Clostridium difficile sin* locus. PLoS Pathog 14, e1006940.

Gene-ID 630	Name 630
CD3030	
CD2666	ptsG-A
CD2667	ptsG-BC
CD3089	
CD3115	bglA
CD3136	bglA
CD3116	bglF
CD3137	bglF
CD3174	gapA
CD3173	pgk
CD3172	tpi
CD3394	pyk
	tkt
CD0882	glgC
CD0883	glgD
CD0885	glgP
CD0884	glgA
CD0758	pflA
CD0759	plfB
CD3282	pflD
CD3283	pflE
CD2164	ldh
CD2966	adhE
CD1059	thlA1
CD1054	bcd2
CD1058	hbd
CD2338	4hbD
CD2339	cat2
CD2340	
CD2341	abfD
CD2342	sucD
CD2343	cat1
CD2344	
nily	
CD1920	eutD
CD0718	fhs
CD0719	fchA
CD0721	
CD0723	
CD0724	
CD0725	
CD0726	
CD0727	
CD0728	
CD0716	cooS
CD0717	

Gene-ID 630	Name 630
CD3468	*atpD*
CD3467	*atpC*
CD3469	*atpG*
CD3470	*atpA*
CD2954	*ntpD*
CD2955	*ntpB*
CD2956	*ntpA*
CD2956A	*ntpF*
CD2957	*ntpC*
CD2958	*ntpE*
CD2959	*ntpK*
CD2960	*ntpI*
CD2670	*appF*
CD2671	*appD*
CD2672	*appA*
CD2673	*appB*
CD2674	*appC*
CD0853	*oppB*
CD0854	*oppC*
CD0855	*oppA*
CD0856	*oppD*
CD0857	*oppF*
CD1548	*hisG*
CD1549	*hisC*
CD1550	*hisB*
CD1551	*hisH*
CD1552	*hisA*
CD1553	*hisF*
CD2198	
CD2200	*hisC*
CD1482	*ssuC*
CD1483	*ssuB*
CD1484	*ssuA*
CD3237	*prdF*
CD3238	
CD3239	*prdE*
CD3240	*prdD*
CD3241	*prdB*
CD3243	
CD2348	*grdD*
CD2349	*grdC*
CD2351	*grdB*
CD2352	*grdA*
CD2354	*grdE*
CD2355	*trxA2*
CD2356	*trxB3*
CD2357	*grdX*

Gene-ID 630	Name 630
CD1062	*acpP*
CD0119	*glmM*
CD0120	*glmS*
CD2239	
CD2240	*nanA*
CD2241	*nanE*
CD1010	*nagA*
CD1011	*nagB*
CD2136	*uppS*
CD2851	*dltC*
CD2852	*dltB*
CD2853	*dltA*
CD2854	*dltD*
CD0059	*secE*
CD3678	*yidC-oxaA1*
CD2831	
CD2830	*zmp1*
CD0873	
CD3512	
CD3513	*pilA* $_1$
CD2305	*pilW*
CD0234	*csrA*
CD0254	*flgD*
CD0255	*flgE*
CD0255A	*flbD*
CD0263	*flhA*
CD3265	
CD3267	
CD1420	*dccA*
CD1421	
CD2384	
CD1515	
CD1616	

Gene-ID<br><br>630	Name<br><br>630
CD1492	
CD1613	*cotA*
CD2400	*cotJB2*
CD3349	*bclA3*
CD0596	
CD0597	*cotJB1*
CD0598	*cotCB*
CD1067	
CD1063B	
CD1063C	
CD1581	
CD1845	

r).
