# Supplementary material for: Clostridium difficile Biofilm: Remodeling Metabolism and Cell Surface to Build a Sparse and Heterogeneously Aggregated Architecture
Source: Front Microbiol. 2018 Sep 12;9:2084. doi: 10.3389/fmicb.2018.02084 (PMC6143707; doi:10.3389/fmicb.2018.02084)
Supplement: Supplementary file 6 [file Image_1.pdf]

Figure S1

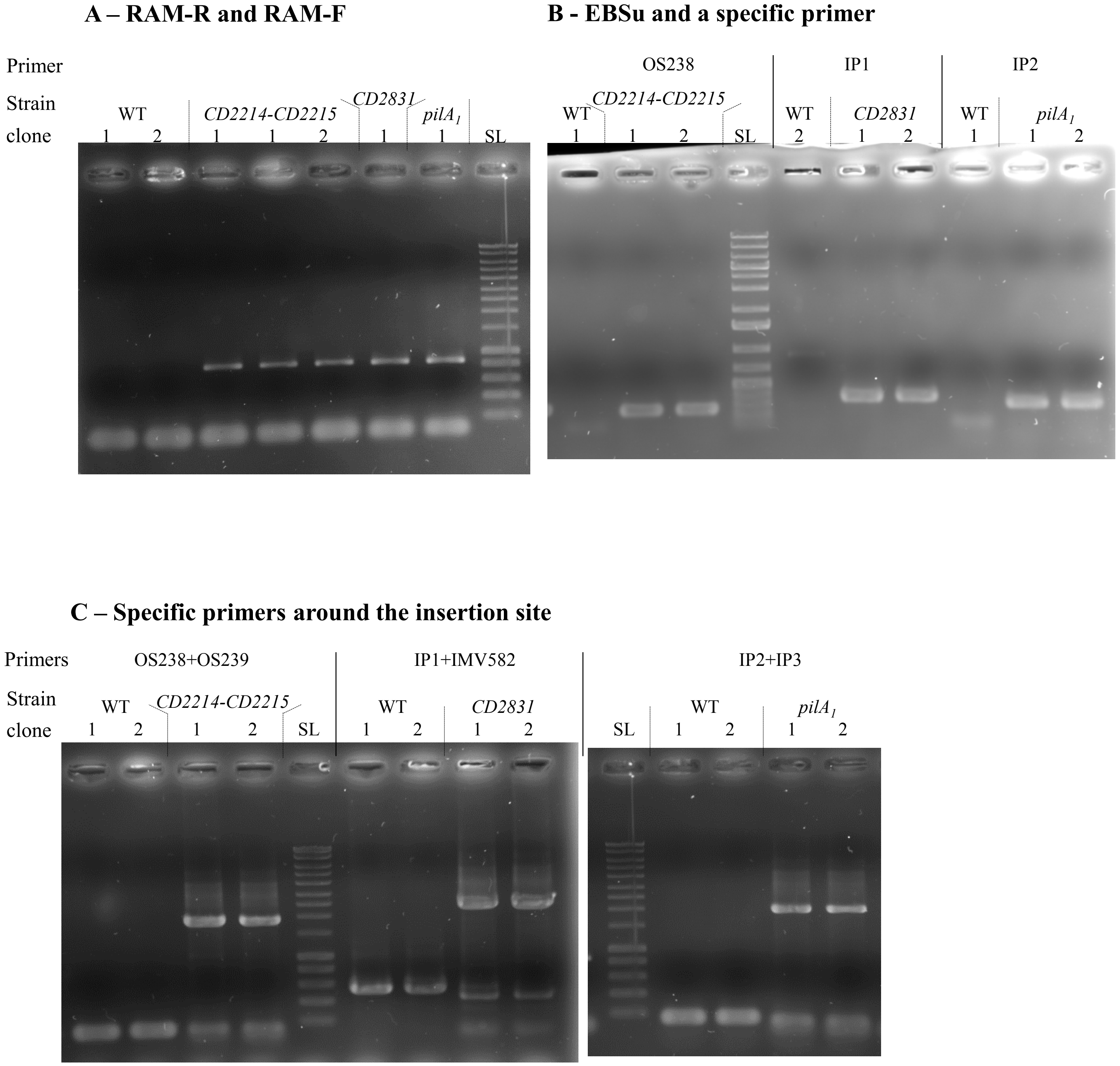

Figure S1. ClosTron mutants: verification of Intron insertion

630Δ*erm* (WT) and its three ClosTron mutants: *CD2214-CD2215*, *CD2831* and *pilA*$_1$, were grown, like in Figure S4, in continuous-flow micro-fermentors full of TYt medium for 72 h in the absence of erythromycin selection. After recovery of biofilm cells and DNA extraction, the stability of Intron insertion into each gene of interest was verified by PCR using the indicated primers (see Table S1 C). The correct splicing of the group I intron was verified using RAM-R and RAM-F primers (A). The correct insertion of the retargeted group II Intron into the gene of interest was verified using either i) EBSu primer (Intron) and a primer specific to the inactivated gene (B) or ii) two specific primers located from either side of the Intron insertion site (C).

SL, Smart Ladder
