# Supplementary material for: Clostridium difficile Biofilm: Remodeling Metabolism and Cell Surface to Build a Sparse and Heterogeneously Aggregated Architecture
Source: Front Microbiol. 2018 Sep 12;9:2084. doi: 10.3389/fmicb.2018.02084 (PMC6143707; doi:10.3389/fmicb.2018.02084)
Supplement: Supplementary file 7 [file Image_2.PDF]

Figure S2

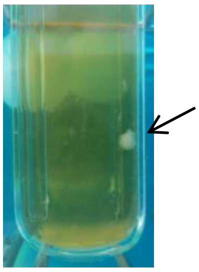

Figure S2. Early biofilm growth of strain 630Δ*erm* in a continuous-flow micro-fermentor Strain 630Δ*erm* was grown as a biofilm in a continuous-flow micro-fermentor like in Figure 1, but the picture was taken after 48h of growth rather than 72h. The bottom of a micro-fermentor still in the incubation bath is shown, with a macro-colony indicated by an arrow.
