# Supplementary material for: Clostridium difficile Biofilm: Remodeling Metabolism and Cell Surface to Build a Sparse and Heterogeneously Aggregated Architecture
Source: Front Microbiol. 2018 Sep 12;9:2084. doi: 10.3389/fmicb.2018.02084 (PMC6143707; doi:10.3389/fmicb.2018.02084)
Supplement: Supplementary file 8 [file Image_3.PDF]

Figure S3

A.

1.

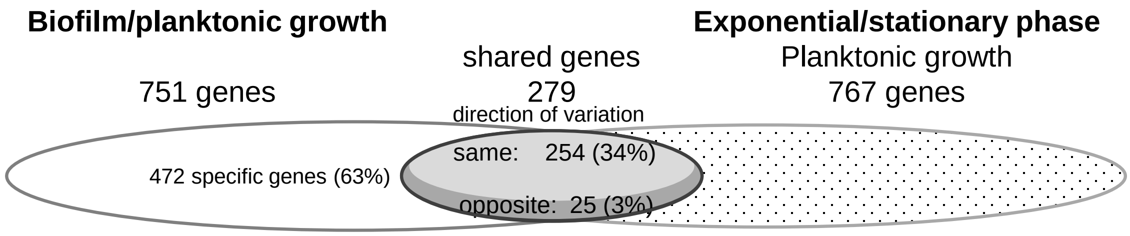

2.

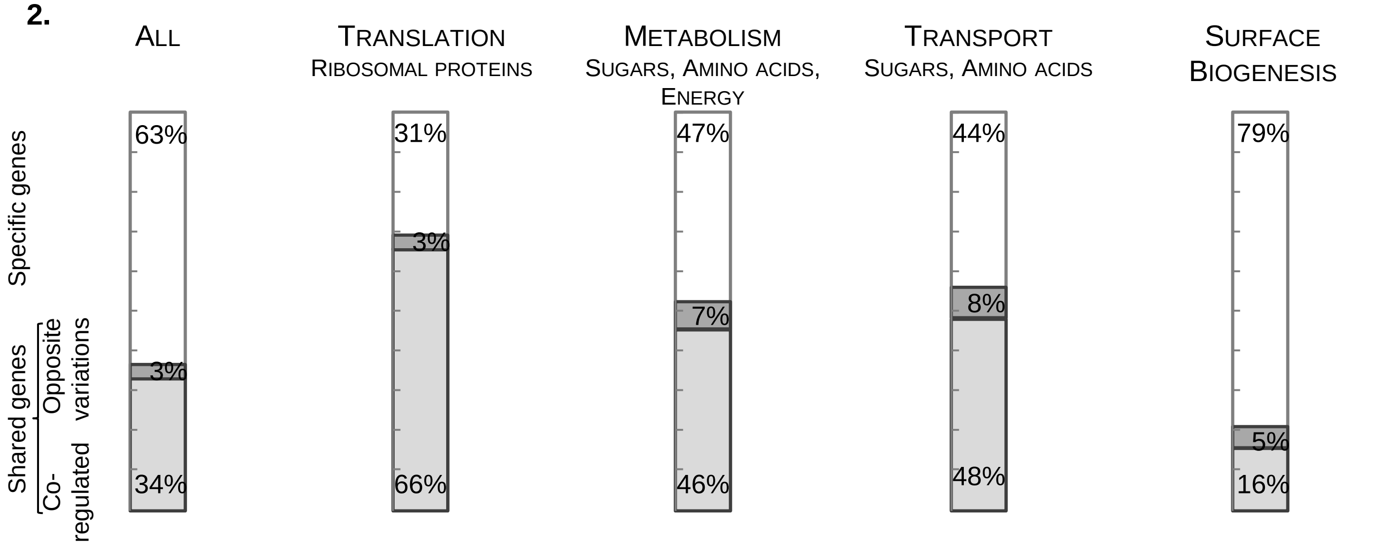

3.

Co-regulated	Translation	Metabolism	Transport	Surface biogenesis
**Up-regulated**	**Ribosomal proteins (25)**<br>**Rpl**<br>**Rpm**<br>**Rps**	**Glycolysis Pgi**<br>**Pyruvate Formate Lyase PflAB**<br>**Pentose Phosphate Pathway Tkt'**<br>**Succinate utilization**<br>**CD2338-43**<br>**Cysteine metabolism**<br>**MalY**	**PTS systems**<br>**CD3027-30**<br>**CD3013-15**<br>**Sugar uptake**<br>**CD2548-50 CD3017**<br>**Succinate uptake**<br>**CD2344**<br>**Oligopeptide uptake**<br>**AppC**<br>**Di- Tri-peptide uptake**<br>**CD3036 CD2260**<br>**Sulfonate ABC uptake**<br>**CD2360-62-65**	**Envelope biogenesis**<br>**AcpP FabF**<br>**GlmMS**<br>**Surface polysaccharide**<br>**LcpB DltB DltC**<br>**Protein export**<br>**YajC PrsA**
**Down-regulated**	*None*	**Glycogen synthesis**<br>**GlgCDAP**<br>**Wood Ljungdahl Pathway**<br>**CD0716-28**<br>**CD3313-14**<br>**Pyruvate Formate Lyase**<br>**PflDE**<br>**Butyrate fermentation**<br>**CD1054-59**<br>**Ptb-CD0715**<br>**Peptidase CD3521**<br>**Glycine reduction GrdC**<br>**ATP Synthase**<br>**NtpABCDE NtpIK**	**Oligopeptide uptake**<br>**OppBCADF**<br>**Branched chain amino acid uptake**<br>**BrnQ1 BrnQ2**<br>**Sulfonate ABC uptake**<br>**SsuA**	***Envelope biogenesis***<br>***UppS Alr2***<br>***Cell wall protein***<br>***Cwp29***

B.

1.

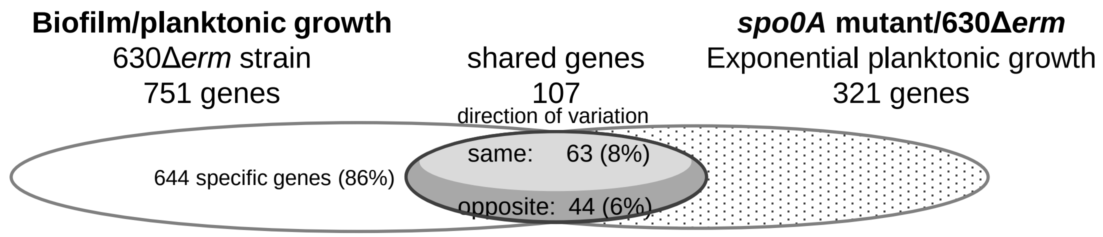

2.

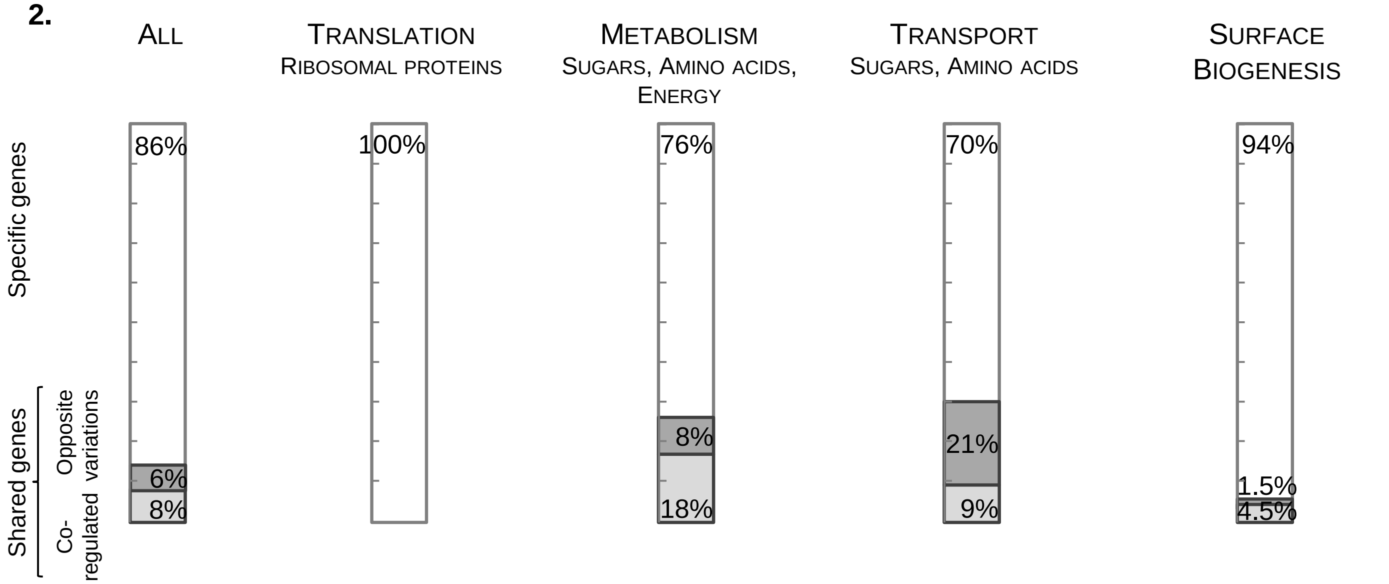

3.

Co-regulated — Up-regulated

Translation	Metabolism	Transport	Surface biogenesis
None	**Pyruvate Formate Lyase PflAB ***	**Succinate uptake CD2344 ***	**Surface polysaccharide DltA DltB ***
	**Succinate utilization CD2338-43 ***	**Di- Tri-peptide uptake CD3036 ***	**Cell wall Protein Cwp10**
		**Amino acid ABC uptake CD2174-75-77**	**Type IV pilin PilW**

Co-regulated — Down-regulated

Translation	Metabolism	Transport	Surface biogenesis
None	**Glycogen synthesis GlgDP ***	**Sulfonate ABC uptake SsuA ***	**Envelope biogenesis UppS ***
	**Wood Ljungdahl Pathway CD3314 ***		**Cell wall protein Cwp29 ***
	**Butyrate fermentation CD1054-59 ***		
	**Peptidase CD3521 ***		
	**Glycine reduction GrdB**		
	**ATP Synthase NtpA NtpI ***		

Figure S3. Comparison of the biofilm/planktonic transcriptome with two other ones

A. Comparison with the exponential/stationary phase transcriptome

The biofilm/planktonic transcriptome (set of genes differentially expressed during biofilm/planktonic growth; Table S2) and the exponential/stationary phase transcriptome (set of genes differentially expressed in the exponential/stationary phase of planktonic growth in TY medium*;* Saujet et al., 2011) are compared.

1. Overlap

The biofilm/planktonic and exponential/stationary phase transcriptomes are represented as elipses, respectively on the left and on the right. The number of shared genes (whose expression varies in the two transcriptomes) is indicated above the overlap region. The expression of shared genes can vary in the same direction in the two transcriptomes (co-regulated genes; light grey) or in opposite directions (dark grey part), and the number of each class of shared genes is indicated in the overlap region. The number of specific genes (whose expression specifically varies in the biofilm/planktonic transcriptome) is indicated in the left elipse.

2. Relative importance of shared genes in the biofilm/planktonic transcriptome per functional category

Four main functional categories (including almost 40% of the genes of the biofilm/planktonic transcriptome) are in capitals. The relative importance of specific and shared genes in the biofilm/planktonic transcriptome is drawn per category as a stacked bar chart. For each category, the relative proportions of specific genes (white box), co-regulated genes (light grey box) and genes regulated in opposite directions (dark grey box), with respect to all genes of the category in the biofilm/planktonic transcriptome, are indicated.

3. Functions of co-regulated genes

The products of co-regulated genes are indicated by their names (or short identification numbers), in red or green characters depending on whether the gene is up- or down-regulated in both transcriptomes. Underlined characters indicate that the gene expression is controlled by CD2214-CD2215 proteins (Figure 6, Table S4) in a direction consistent with its variation in both transcriptomes, i. e. that CD2214-CD2215 proteins could be responsible for its regulation in both transcriptomes.

B. Comparison with the *spo0A* mutant/630Δ*erm* transcriptome

Like in A except that the biofilm/planktonic transcriptome and the *spo0A* mutant/630Δ*erm* transcriptome (genes differentially expressed in the *spo0A* mutant/630Δ*erm* after planktonic growth till the exponential phase in Wilson's broth*;* Pettit et al., 2014) are compared.

1. Overlap

The *spo0A* mutant/630Δ*erm* transcriptome is on the right.

2. Relative importance of shared genes in the biofilm/planktonic transcriptome per functional category

3. Functions of co-regulated genes

***** labels a co-regulated gene whose expression also varies in the same direction not only in the biofilm/planktonic and the *spo0A* mutant/630Δ*erm* transcriptomes, but also in the exponential/stationary phase transcriptome.

References

Pettit, L.J., Browne, H.P., Yu, L., Smits, W.K., Fagan, R.P., Barquist, L., Martin, M.J., Goulding, D., Duncan, S.H., Flint, H.J., Dougan, G., Choudhary, J.S., and Lawley, T.D. (2014). Functional genomics reveals that *Clostridium difficile* Spo0A coordinates sporulation, virulence and metabolism. *BMC Genomics* 15**,** 160.

Saujet, L., Monot, M., Dupuy, B., Soutourina, O., and Martin-Verstraete, I. (2011). The key sigma factor of transition phase, SigH, controls sporulation, metabolism, and virulence factor expression in *Clostridium difficile*. J Bacteriol 193, 3186-3196.
