# Supplementary material for: Clostridium difficile Biofilm: Remodeling Metabolism and Cell Surface to Build a Sparse and Heterogeneously Aggregated Architecture
Source: Front Microbiol. 2018 Sep 12;9:2084. doi: 10.3389/fmicb.2018.02084 (PMC6143707; doi:10.3389/fmicb.2018.02084)
Supplement: Supplementary file 9 [file Image_4.PDF]

Figure S4

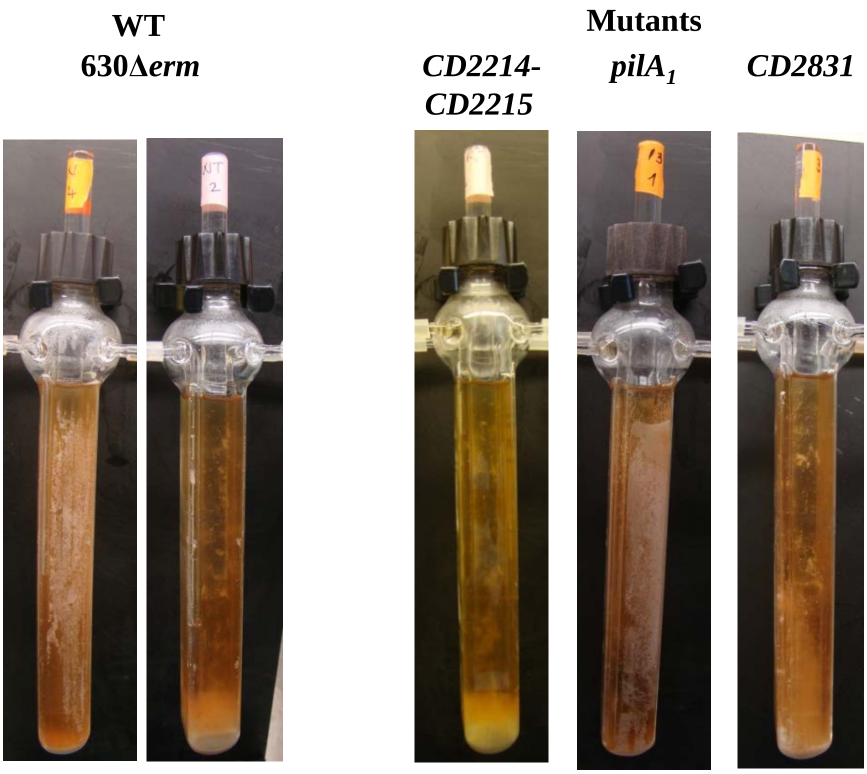

Figure S4. Biofilm formation by the parental and mutant strains in continuous-flow micro-fermentors

630Δ*erm* and three inactivation mutants: *CD2214-CD2215*, *pilA*$_1$ *and CD2831* were grown in continuous-flow micro-fermentors full of TYt medium for 72 h like in Figure 1, in two independent experiments and using at least two independent clones of each strain. Representative pictures are shown.
