# Supplementary material for: Clostridium difficile Biofilm: Remodeling Metabolism and Cell Surface to Build a Sparse and Heterogeneously Aggregated Architecture
Source: Front Microbiol. 2018 Sep 12;9:2084. doi: 10.3389/fmicb.2018.02084 (PMC6143707; doi:10.3389/fmicb.2018.02084)
Supplement: Supplementary file 10 [file Image_5.PDF]

Figure S5

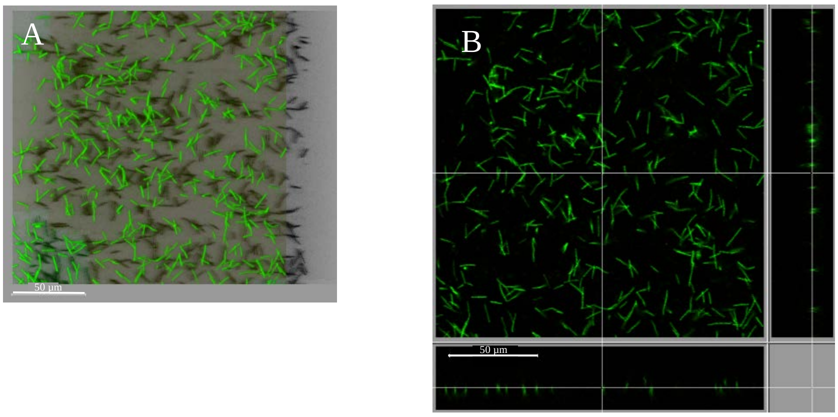

Figure S5. Adhesion of strain 630Δ*erm* cells to polystyrene in a micro-titer plate

An over-night pre-culture of strain 630Δ*erm* grown in TYt medium was diluted in the same medium in a 96-well polystyrene micro-titer plate and incubated for 2h at 37°C to allow cell adhesion as in Figure 8. After removal of non-adhesive cells and addition of fresh TYt medium, adhesive cells were fixed or not in the presence of acetic acid 50%, further incubated for 2 days, stained using a Live dead kit and finally observed by CSLM (all procedures except fixation were as in Figure 8). Biofilm growth was, as previously, started from unfixed adhesive cells (positive control) and the resulting 48h-old biofilm was found to display the same 3D architecture as in Figure 8 A and B, as expected. In parallel, fixed cells representing initially adhesive cells were also observed. Pictures of a representative experiment are shown: a 3D projection upside view, with its shadow on the right (A) and a section view close to the surface (B), with the white bar indicating the scale (50 µm). Immediately after adhesion, cells are present in a discrete number in each field and form a discontinuous mono-layer at the polystyrene surface.
