# Supplementary material for: Clostridium difficile Biofilm: Remodeling Metabolism and Cell Surface to Build a Sparse and Heterogeneously Aggregated Architecture
Source: Front Microbiol. 2018 Sep 12;9:2084. doi: 10.3389/fmicb.2018.02084 (PMC6143707; doi:10.3389/fmicb.2018.02084)
Supplement: Supplementary file 11 [file Image_6.PDF]

Figure S6

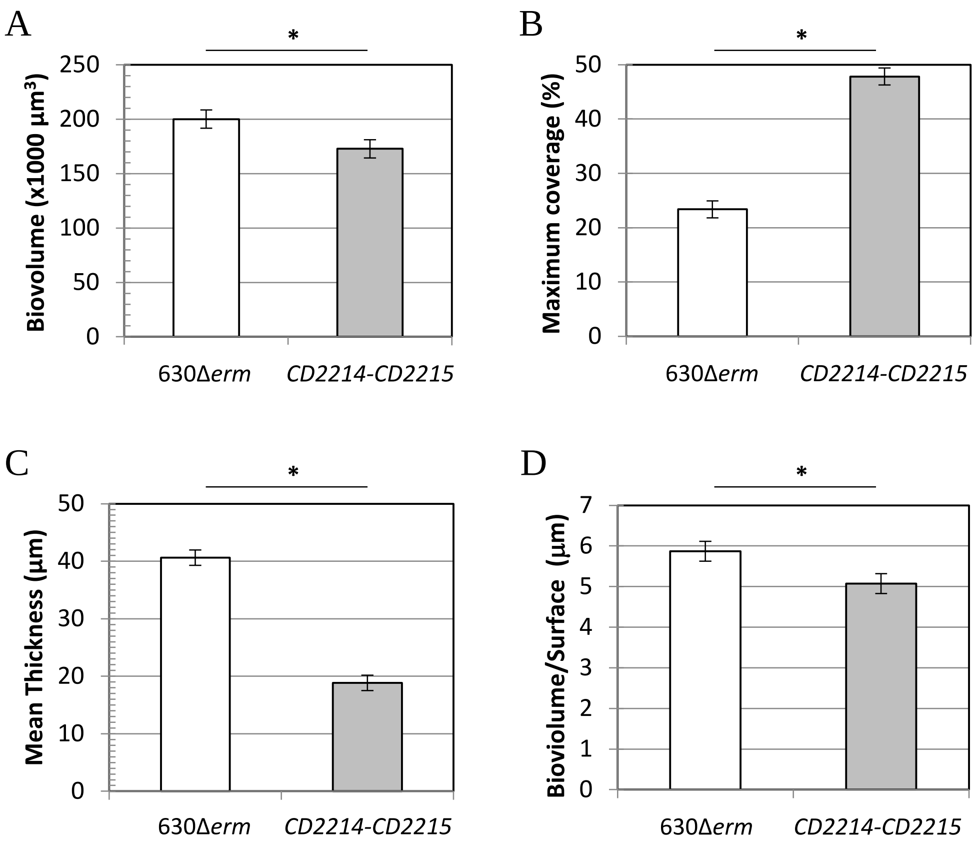

Figure S6. Comparison of the biofilms formed by strain 630Δ*erm* and its *CD2214-CD2215* mutant

After growth in 96-well micro-titer plates, intact biofilms of either the parental strain 630Δ*erm* (in white) or its *CD2214-CD2215* mutant (in grey) were observed by CLMS as described in Figure 8. Data were recovered and four parameters characterizing the 3D architecture of each biofilm: biovolume (A), maximum coverage (B), mean thickness (C) and biovolume/surface ratio (D) were quantified using ICY software. The results of independent experiments using independent clones of each strain are shown as histograms. A statistical analysis was performed and stars (*) indicate a significant difference between the biofilms of the parental and mutant strains.
