# Supplementary material for: Clostridium difficile Biofilm: Remodeling Metabolism and Cell Surface to Build a Sparse and Heterogeneously Aggregated Architecture
Source: Front Microbiol. 2018 Sep 12;9:2084. doi: 10.3389/fmicb.2018.02084 (PMC6143707; doi:10.3389/fmicb.2018.02084)
Supplement: Supplementary file 12 [file Image_7.PDF]

Figure S7

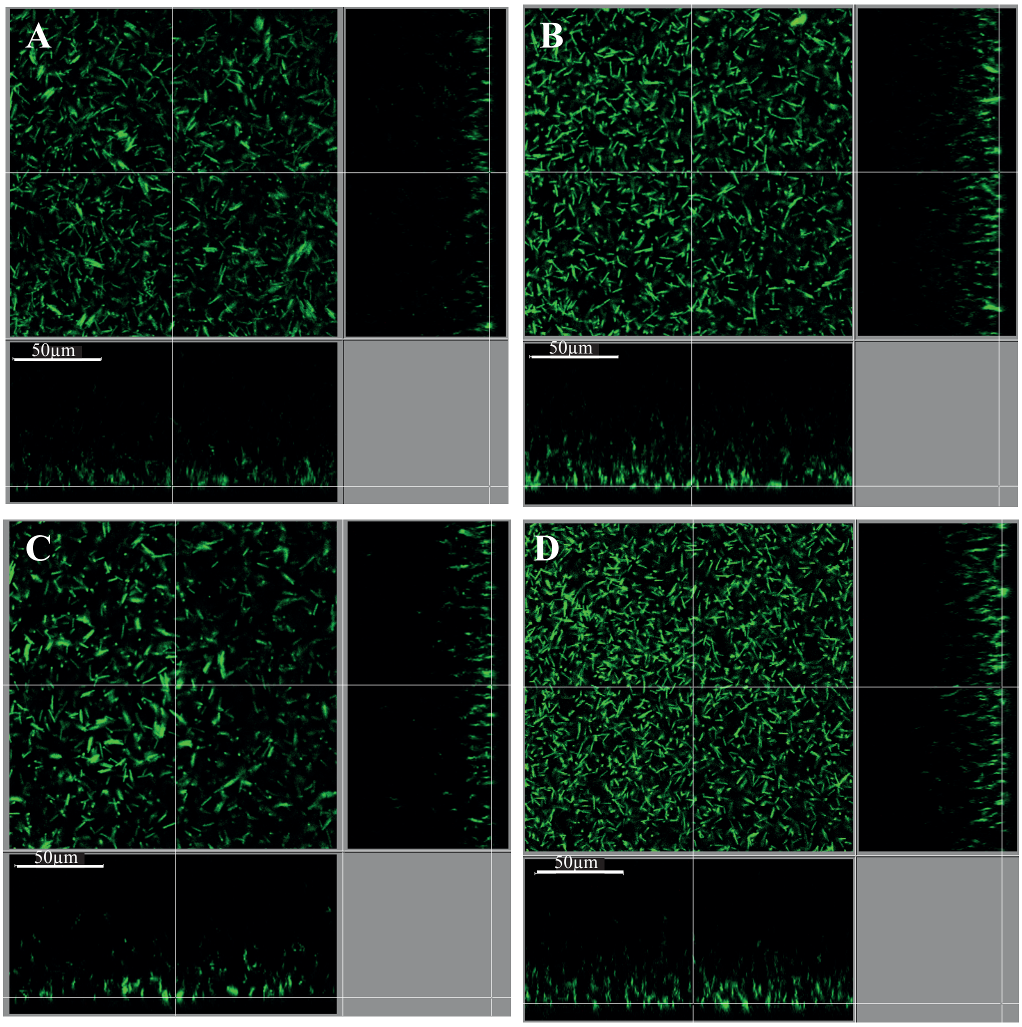

Figure S7. Intact biofilm architecture of *CD2214-CD2215* mutant and complemented strain

Biofilm growth, staining and intact biofilm observation by CLSM were as described in Figure 8. A section view close to the surface is shown for the biofilms of the following strains: the parental strain 630Δ*erm* bearing the empty pMTL84121 vector (A) and the *CD2214-CD2215* mutant bearing the same empty vector (B) or derivative plasmids where either the entire *CD2214-CD2215* operon (C) or *CD2214* gene alone are cloned (D). The white bar indicates the scale (50 µm). Data were recovered, quantified and analysed as in Figure S6, but differences between strains were found not to be statistically significant (not shown).
