# Supplementary material for: Clostridium difficile Biofilm: Remodeling Metabolism and Cell Surface to Build a Sparse and Heterogeneously Aggregated Architecture
Source: Front Microbiol. 2018 Sep 12;9:2084. doi: 10.3389/fmicb.2018.02084 (PMC6143707; doi:10.3389/fmicb.2018.02084)
Supplement: Supplementary file 13 [file Image_8.pdf]

Figure S8

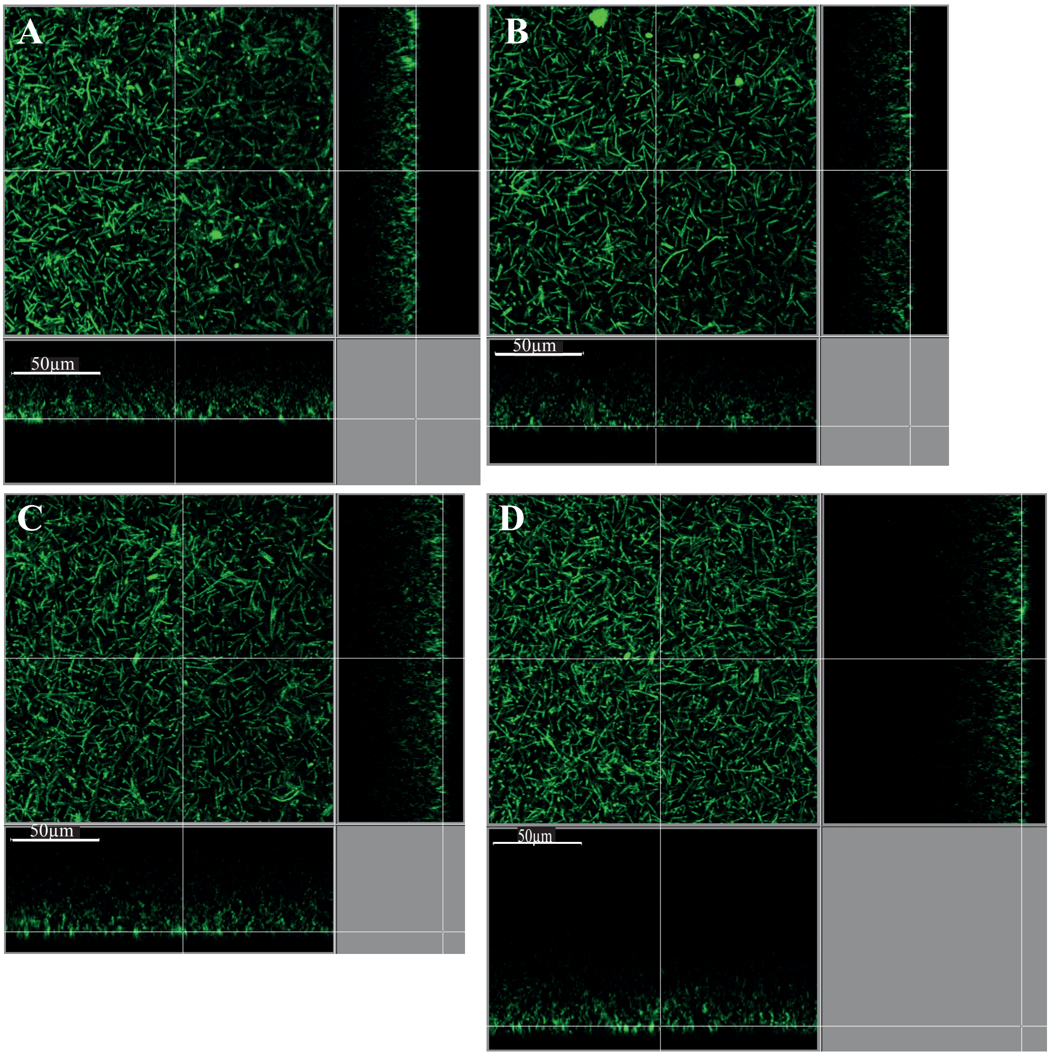

Figure S8. Intact biofilm architecture of *pilA*$_1$ and *CD2831* mutant strains
Biofilm were grown from adhesive starter cells in TYt medium for 24h and in the presence of anhydro-tetracycline inducer as described in Figure 9. Intact biofilm staining and observation by CLMS were as described in Figure 8. A section view close to the surface is shown for the biofilm of each strain, with a white bar indicating the scale (50 µm). The following strains bearing the same empty vector (p) are shown: the parental strain (630Δ*erm* p; in A and C), *pilA*$_1$ (*pilA*$_1$ p; in B) and *CD2831* mutants (*CD2831* p; in D). After data recovery and quantification of biofilm parameters, a statistical analysis revealed no significant difference between the parental and mutant strains (not shown).
