# Supplementary material for: Clostridium difficile Biofilm: Remodeling Metabolism and Cell Surface to Build a Sparse and Heterogeneously Aggregated Architecture
Source: Front Microbiol. 2018 Sep 12;9:2084. doi: 10.3389/fmicb.2018.02084 (PMC6143707; doi:10.3389/fmicb.2018.02084)
Supplement: Supplementary file 14 [file Image_9.pdf]

Figure S9

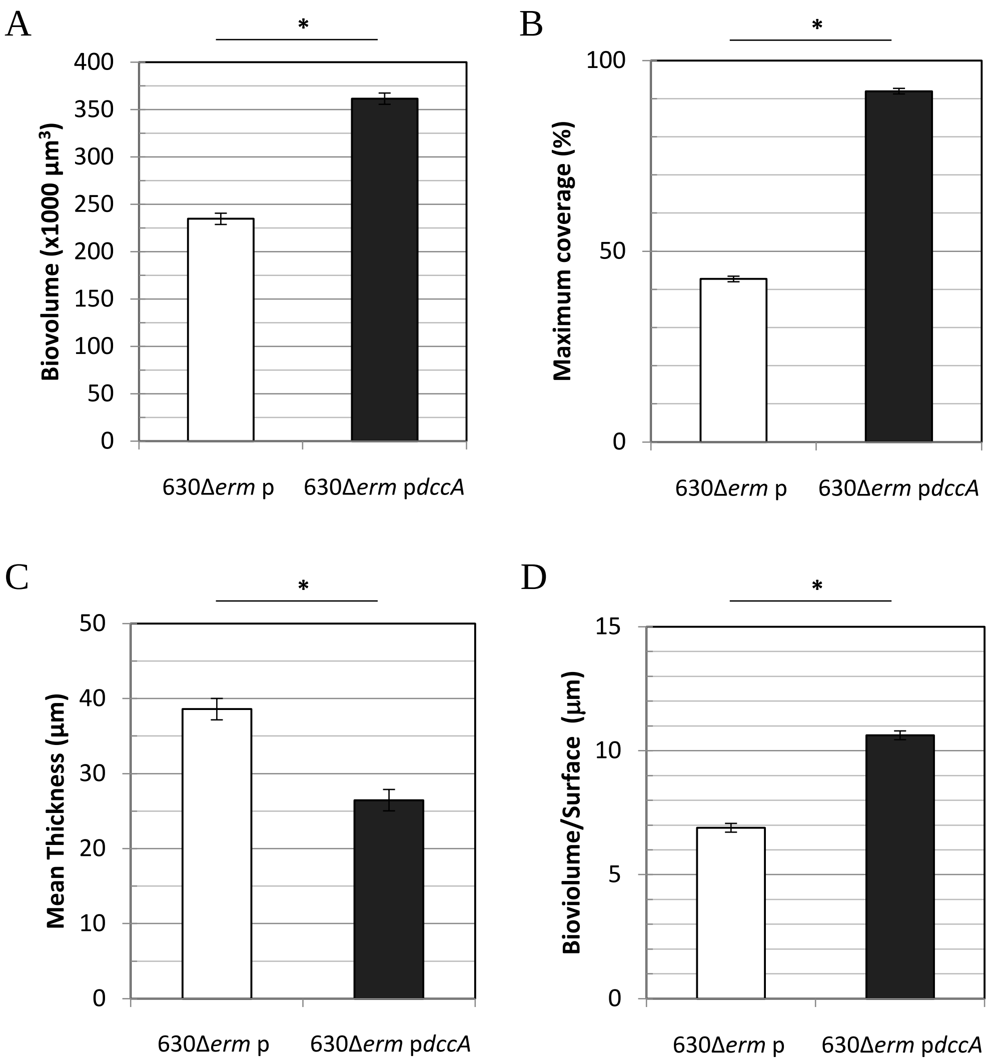

Figure S9. Comparison of the biofilms formed by strain 630Δ*erm* over-expressing or not *dccA*

Biofilms were grown from adhesive cells in TYt medium in the presence of inducer as described in Figure 9. Intact biofilms were stained and observed by CLMS as described in Figure 8. Data were recovered and four parameters characterizing the 3D architecture of each biofilm: biovolume (A), maximum coverage (B), mean thickness (C) and biovolume/surface ratio (D) were quantified using ICY software as in Figure S6. The results of two experiments using independent clones for each strain are shown as histograms, with 630Δ*erm* p*dccA*, the strain over-expressing *dccA*, in dark grey, and 630Δ*erm* p, the control strain bearing the empty vector, in white. A statistical analysis was performed and stars (*) indicate a significant difference between the two strains.
