# Supplementary material for: Clostridium difficile Biofilm: Remodeling Metabolism and Cell Surface to Build a Sparse and Heterogeneously Aggregated Architecture
Source: Front Microbiol. 2018 Sep 12;9:2084. doi: 10.3389/fmicb.2018.02084 (PMC6143707; doi:10.3389/fmicb.2018.02084)
Supplement: Supplementary file 15 [file Image_10.pdf]

Figure S10

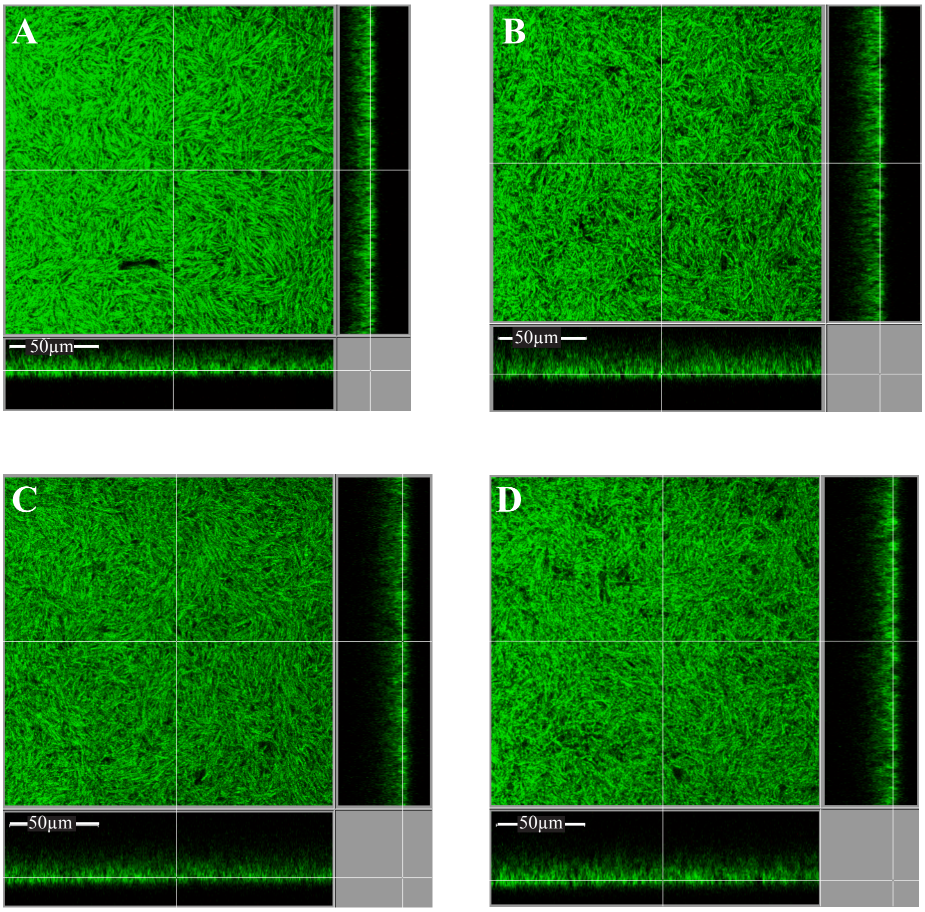

Figure S10. Intact biofilm architecture of *pilA*$_1$ and *CD2831* mutant strains over-expressing *dccA*

Biofilms were grown from adhesive cells in TYt medium for 24h in the presence of anhydro-tetracycline inducer as described in Figure 9. At the end of growth, intact biofilms were stained and observed as described in Figure 8. For each strain, a representative section view close to the surface is shown, with the white bar indicating the scale (50 µm). The parental (630Δ*erm* p*dccA* in A and C) and mutant strains over-expressing *dccA* (*pilA*$_1$ p*dccA* in B and *CD2831* p*dccA* in D) are shown.
